# Elemental concentration and spatial distribution of wild edible fruits and implications for dietary mineral intake in Ethiopia

**DOI:** 10.1038/s41598-025-26400-7

**Published:** 2025-11-27

**Authors:** Diriba B. Kumssa, Tristan Dew, Zerihun Kebebew, Dawd Gashu, Obssi D. Hora, Feyera Senbeta, Tamiru K. Ayante, Musa A. Hamido, Kassaye Tolessa, Martin R. Broadley

**Affiliations:** 1 School of Biosciences, University of Nottingham, Sutton Bonington, UK; 2 College of Agriculture and Veterinary Medicine, Jimma University, Jimma, Ethiopia; 3 Centre for Food Science and Nutrition, Addis Ababa University, Addis Ababa, Ethiopia; 4 National Agricultural Biotechnology Research Centre, Ethiopian Institute of Agricultural Research, Holeta, Ethiopia; 5 Department of Biological Sciences, Faculty of Sciences, Botswana University of Agriculture and Natural Resources, Gaborone, Botswana; 6 College of Agriculture and Natural Resources, Madda Walabu University, Robe, Ethiopia; 7 Fedis Agricultural Research Centre, Oromia Agricultural Research Institute, Harar, Ethiopia; 8 Food Science and Nutrition Research Directorate, Ethiopian Institute of Agricultural Research, Addis Ababa, Ethiopia; 9 Rothamsted Research, Harpenden, Hertfordshire UK

**Keywords:** Biodiversity, Calcium, Eastern Afromontane Biodiversity hotspot, Iron, Lianas, Magnesium, Selenium, Shrubs, Trees, Zinc, Biochemistry, Ecology, Natural variation in plants, Nutrition

## Abstract

Ethiopia faces challenges of food insecurity, malnutrition, and biodiversity loss. The predominantly rural population relies on staple cereals, leading to deficiencies in essential micronutrients. Ethiopia’s Eastern Afromontane biodiversity hotspot hosts a variety of wild edible fruits (WEFs) that could help alleviate these deficiencies. However, WEFs are stigmatised as “food-for-the-poor” and remain underutilised partly due to limited data on their nutritional value. This study is the first to systematically assess the elemental composition of 23 wild and four cultivated fruit species from Oromia and Southern Nations Nationalities and Peoples (SNNP) regions of Ethiopia. Using inductively coupled plasma mass spectrometry, we found several WEFs to be rich in Ca, Fe, Mg, and Se, often surpassing levels in cultivated fruits. A 100 g serving of these fresh fruits could provide up to 40% of recommended nutrient intakes for adolescent boys. Analysis of soil samples collected from fruit harvesting sites revealed significant correlations between soil and fruit elemental concentrations for several minerals, highlighting the importance of soil properties in determining the nutritional quality of WEFs. Species distribution modelling for 11 selected WEF species identified suitable habitats across southern Ethiopia, with significant spatial variation, suggesting opportunities for targeted promotion and conservation. Integrating WEFs into diets and agroforestry systems could enhance nutrition and biodiversity. Further research on bioavailability, domestication, and policy engagement is recommended.

**Supplementary Information:**

The online version contains supplementary material available at 10.1038/s41598-025-26400-7.

## Introduction

Ethiopia, home to over 120 million people^1^, is facing critical challenges in terms of food and nutritional security as well as biodiversity conservation. The country’s predominantly rural population depends on small-scale agriculture for their livelihoods^2–4^, which has implications for both dietary diversity and environmental sustainability. The reliance on staple crops^5,6^, due to limited land and agricultural resources^2–4^, contributes to dietary deficiencies in essential minerals^7^, exacerbating the burden of disease^8^.

Ethiopia harbours a significant portion of the Eastern Afromontane biodiversity hotspot, including thousands of plant species^9^. However, the pressure of an increasing population has led to deforestation and biodiversity loss^10,11^, with implications for both the environment^11,12^ and food security. Despite the global recommendation for a diet rich in fruits and vegetables^13^ to ensure nutritional adequacy, access to and affordability of such nutritious foods remain a global challenge in general and in Ethiopia in particular^14^, where diets are predominantly cereal-based and often lack diversity^7^.

Ethiopia’s forests and woodlands are sources of wild edible fruits^15,16^, which could play a crucial role in alleviating dietary micronutrient (mineral and vitamin) deficiencies^17,18^. However, WEFs are frequently stigmatised as “food-for-the-poor” and are generally consumed by the broader community only during periods of food scarcity. Consequently, these fruits have been underutilised and overlooked in agricultural, environmental, and health policies, partly due to insufficient data on their nutritional value.

The nutritional value of fruits, particularly their mineral element composition, is strongly influenced by the soil in which the plants grow. Soil properties such as pH, organic matter content, and mineral availability directly affect plant uptake of essential elements^19,20^. This soil–plant relationship is particularly important in the diverse agroecological zones of Ethiopia, where soil types and properties vary considerably^6^. Understanding these relationships is crucial for identifying areas where WEFs might naturally accumulate higher concentrations of nutritionally important elements, as well as for developing potential cultivation strategies for these species.

The distribution of wild fruit species across Ethiopia’s landscape is determined by complex interactions between biotic and abiotic factors, including climate and soil conditions^21,22^. Climate change and land-use modifications are altering these distributions^11,23–25^, potentially threatening the availability of these nutritional resources^26^. Species distribution modelling can identify suitable habitats for these fruit species^27,28^, which is essential for conservation planning and for identifying potential areas for domestication or in-situ management. Furthermore, understanding the relationship between species distribution, soil conditions, and fruit nutritional quality can help identify priority areas where conservation efforts might yield the greatest nutritional benefits for local communities.

Addressing these interconnected challenges, our study presents the first systematic assessment of the elemental composition of 23 wild, and four cultivated edible fruit species found across Ethiopia’s diverse agroecosystems. We specifically aimed to:

1. Determine the mineral element composition of selected wild and cultivated fruit species to assess their potential contribution to addressing nutritional deficiencies,
2. Investigate the relationship between soil mineral content and fruit elemental concentrations to understand how environmental factors influence the nutritional quality of these fruits, and
3. Model the potential distribution of selected wild fruit species across southern Ethiopia to identify areas suitable for conservation, sustainable harvesting, or potential domestication.

By documenting the mineral nutritional content of these edible fruits and relating it to soil conditions, and predicting their potential spatial distribution, this research aims to highlight their dietary value, encourage their domestication and conservation, and support their inclusion in dietary recommendations. This integrated approach not only aims to improve dietary diversity and nutritional status among Ethiopians but also to foster a greater appreciation for the country’s rich plant biodiversity. Engaging local communities and policy makers in the sustainable use and management of these resources could ensure their availability and nutritional benefits for future generations, contributing to the dual goals of improving food security and as non-timber forest products which maintains the vegetation for conserving biodiversity in Ethiopia.

## Materials and methods

**Fig. 1:**
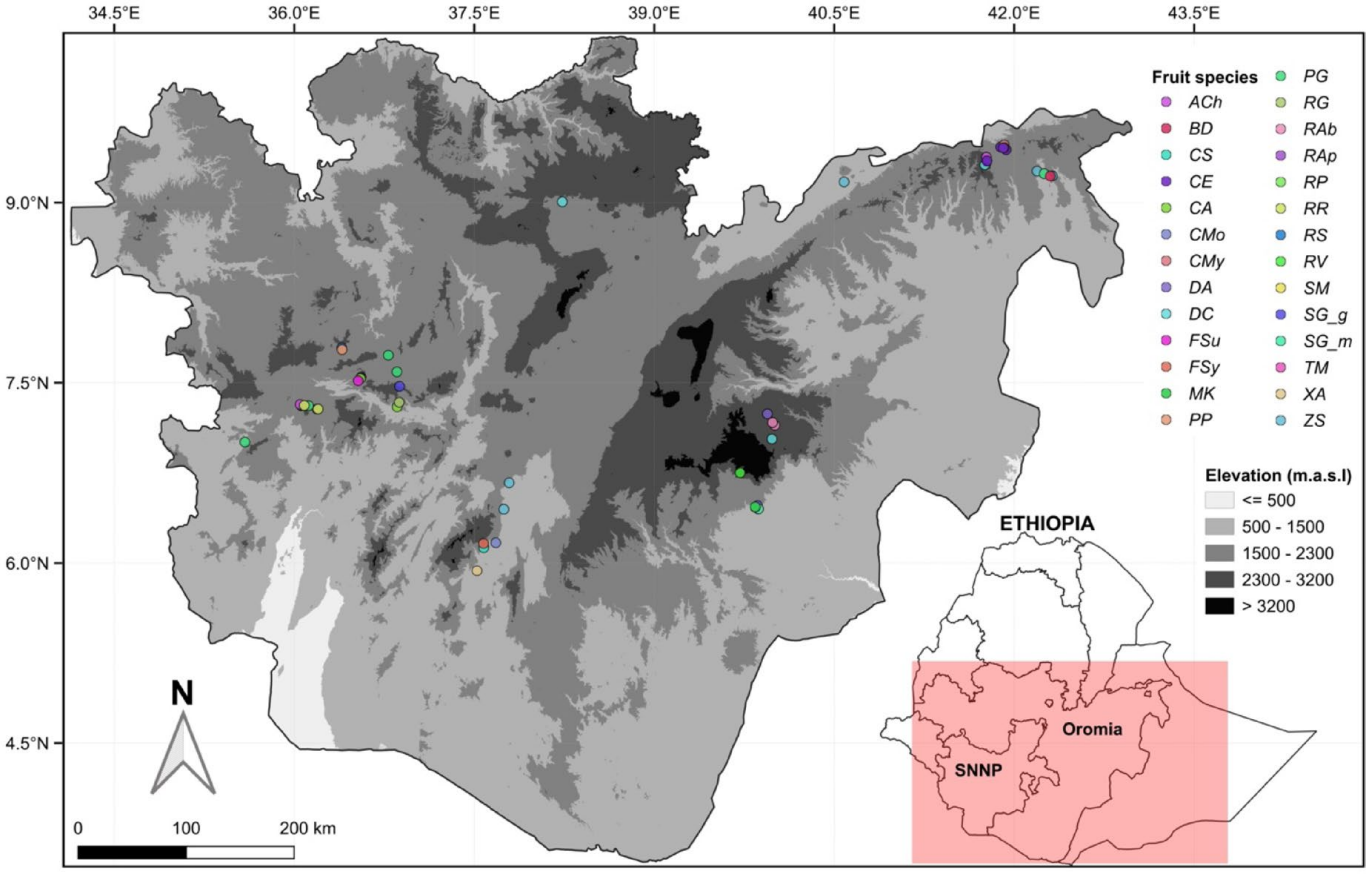
Sampling sites of edible fruits (coloured circles) across the Oromia and SNNP regions of Ethiopia. Elevation (meters above sea level; m.a.s.l.) is shown in greyscale. The elevation data was obtained from the digital elevation model of the Shuttle Radar Topography Mission^53^. The shape files were acquired from the Global Administrative District Map (GADM) Version 4.1^54^. Refer to Table 1 for the scientific names corresponding to the species’ acronyms.

The research was carried out in selected areas of the Oromia and Southern Nations Nationalities and Peoples (SNNP) regions of Ethiopia (Fig. 1) where WEFs grow. These two regions have a combined total population of around 58 million people^29^. They are renowned for their extensive forest cover, practicing various agroforestry systems, and have diverse agroclimatic zones ranging from low-lying arid lands to high mountain peaks.

### Edible fruit species selection

Important wild edible fruits (WEFs) growing in Ethiopia were identified and selected through ethnobotanical literature reviews^16–18,30–34^. From these reviews, about 200 indigenous woody plant species that bear edible fruits were identified and out of which 138 were selected as priority and used to guide fruit sampling. After determining the priority edible fruit-bearing plant species and the fruiting season, subsequent field sampling was determined by analysing literature data^35^ and personal communication with forestry and agriculture development workers, and researchers living and working in the areas where these plants grow.

### Field sampling of fruits

**Fig. 2:**
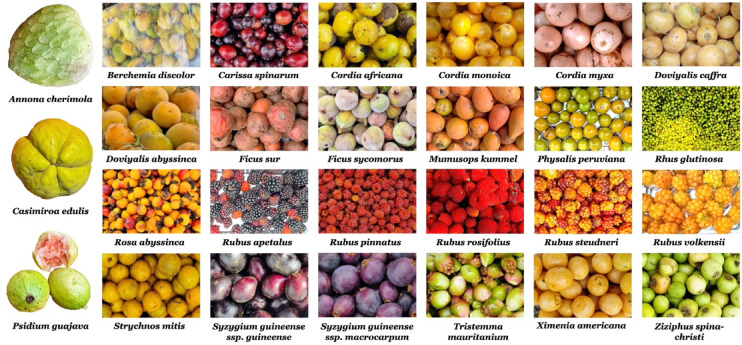
Photographs of the sampled fresh edible fruits (Photo: Diriba B Kumssa). *Annona cherimola*, *Casimiroa edulis*, *Doviyalis caffra* and *Psidium guajava* are cultivated edible fruits (CEF) species. The remaining 23 species are wild edible fruits (WEFs).

The planning of field sampling involved using the Global Biodiversity Information Facility (GBIF) data, which serves as a repository for species taxonomic and occurrence data^36^. To create a comprehensive plan, we combined the occurrence geographic coordinates of native plants in Ethiopia that bear edible fruits, obtained from GBIF, with the zonal thematic geolocation information provided in a study by Teketay, et al.^35^ that focused on wild edible plants. Teketay, et al.^35^ had compiled the zonal WEF occurrence and fruiting phenology data using an outdated administrative map of Ethiopia, which was converted into a digital format using QGIS^37^. By overlaying this digitized map with the WEF point occurrence geolocation data from GBIF, we were able to generate a fruiting calendar for the various WEFs. This calendar was instrumental in planning field sampling, allowing us to target a time when numerous species were fruiting simultaneously, thereby reducing the need for several field trips to Ethiopia. In total, fruits from 23 wild, and four cultivated plant species representing 11 orders and 15 families (see Table 1 and Fig. 2) and growing in various parts of Ethiopia (Fig. 1) were sampled during October–November 2022 and March–April 2023. Fruit samples from cultivated species were obtained from farmers’ agricultural fields, whereas wild species fruit samples were gathered from their native environments. The identification of the plants providing the edible fruits was conducted using digital images of the plant parts by Mr. Melaku Wondafrash from the National Herbarium at Addis Ababa University, and Dr. Feyera Senbeta, a coauthor of this paper. No voucher specimens of the plants bearing the edible fruits were preserved.

**Table 1:** Taxonomic classification^55^, species acronyms and growth forms of the sampled edible fruit bearing plants. All plants are Angiosperms.

Order	Family	Species	Species acronym	Growth form
Magnoliales	Annonaceae	*Annona cherimola* Mill	*ACh*	Shrub
Rosales	Rhamnaceae	*Berchemia discolor* (Klotzsch) Hemsl	*BD*	Tree
Gentianales	Apocynaceae	*Carissa spinarum* L	*CS*	Shrub
Amborellales	Amborellaceae	*Casimiroa edulis* La Llave & Lex	*CE*	Tree
Boraginales	Cordiaceae	*Cordia africana* Lam	*CA*	Tree
Boraginales	Cordiaceae	*Cordia monoica* Roxb	*CMo*	Shrub
Boraginales	Cordiaceae	*Cordia myxa* L	*CMy*	Tree
Malpighiales	Salicaceae	*Dovyalis abyssinica* (Rich.) Warb	*DA*	Tree
Malpighiales	Salicaceae	*Dovyalis caffra* (Hook.fil. ex Harv. & Sond.) Warb	*DC*	Shrub/Tree
Rosales	Moraceae	*Ficus sur* Forssk	*FSu*	Tree
Rosales	Moraceae	*Ficus sycomorus* L	*FSy*	Tree
Ericales	Sapotaceae	*Mimusops kummel* Bruce ex A.DC	*MK*	Tree
Solanales	Solanaceae	*Physalis peruviana* L	*PP*	Liana/Herb
Myrtales	Myrtaceae	*Psidium guajava* L	*PG*	Shrub/Tree
Sapindales	Anacardiaceae	*Rhus glutinosa* Hochst. ex A.Rich	*RG*	Shrub
Rosales	Rosaceae	*Rosa abyssinica* R.Br	*RAb*	Shrub
Rosales	Rosaceae	*Rubus apetalus* Poir	*RAp*	Liana/Shrub
Rosales	Rosaceae	*Rubus pinnatus* Willd	*RP*	Liana/Shrub
Rosales	Rosaceae	*Rubus rosifolius* Sm	*RR*	Liana/Shrub
Rosales	Rosaceae	*Rubus steudneri* Schweinf	*RS*	Liana
Rosales	Rosaceae	*Rubus volkensii* Engl	*RV*	Liana/Shrub
Gentianales	Loganiaceae	*Strychnos mitis* S.Moore	*SM*	Tree
Myrtales	Myrtaceae	*Syzygium guineense ssp. Guineense* (Willd.) DC	*SG_g*	Tree
Myrtales	Myrtaceae	*Syzygium guineense ssp. Macrocarpum* (Engl.) F.White	*SG_m*	Tree
Myrtales	Melastomataceae	*Tristemma mauritianum* J.F.Gmel	*TM*	Herb
Santalales	Ximeniaceae	*Ximenia americana* L	*XA*	Shrub
Rosales	Rhamnaceae	*Ziziphus spina-christi* (L.) Desf	*ZS*	Shrub /Tree

The research complied with relevant institutional, national, and international guidelines and legislation. Before conducting surveys and field sampling of edible fruits, pre-informed consent was obtained from the Ethiopian Biodiversity Institute in May 2022. Following the completion of field sampling, a material transfer agreement was signed among the lead researcher (Dr. Diriba B. Kumssa), the University of Nottingham, and the Ethiopian Biodiversity Institute, in accordance with the Nagoya Protocol on Access and Benefit Sharing of biodiversity resources. This agreement covered the export of two rounds of freeze-dried and milled fruit samples, as well as air-dried soil samples. Prior to exporting these samples from Ethiopia to the United Kingdom for biochemical analysis, a phytosanitary certificate was secured from the Ethiopian Agriculture Authority and the Ethiopian National Soil Laboratory.

### Wild edible fruits ethnobotanical data and metadata

Ethnobotanical information regarding the use of the wild fruits for human consumption, including who usually consumes them, their taste and colour when ripe, and modes of fruit consumption (see Table 2) were collected using a structured KoBoToolbox electronic questionnaire (see Appendix 1). Prior to conducting the survey with local adults, the study received an ethical approval from the University of Nottingham, School of Biosciences Research Ethics Committee (SBREC), approval number SBREC202122024FEO and the research was performed in accordance with the relevant guidelines and regulations. Survey participants were provided with information about the study and their informed consent was sought prior to interview. Furthermore, fruit and soil georeferenced metadata were collected using an electronic questionnaire implemented in KoBoToolbox (Appendix 2).

**Table 2:** Acronyms of the plant species that produce edible fruits, alongside their local names, the languages of those names, the taste characteristics of the fruits when ripe, who consumes these fruits, and how they are typically consumed.

Species acronyms	Local name	Local name language	Taste of ripe fruits	Consumers	Mode of consumption
*ACh*	Gishxaa	Afaan Oromoo	Sweet	Everybody	Fresh-deseeded
*BD*	Jajjaba	Afaan Oromoo	Sweet	Everybody	Fresh-peeled-deseeded
*CS*	Agamsa/Laadee	Afaan Oromoo/Gamogna	Sweet	Everybody	Fresh-whole
*CE*	Hambaadhadhaa	Afaan Oromoo	Sour, sweet	Everybody	Fresh-deseeded
*CA*	Waddeessa	Afaan Oromoo	Sweet	Everybody	Fresh-peeled-deseeded
*CMo*	Waddeessa	Afaan Oromoo	Sweet	Everybody	Fresh-peeled-deseeded
*CMy*	Waddeessa	Afaan Oromoo	Sweet	Everybody	Fresh-peeled-deseeded
*DA*	Keeshummoo/Koomshoo	Afaan Oromoo	Sweet	Everybody	Fresh-peeled
*DC*	Koshommii/Koomshoo	Afaan Oromoo	Sour	Children	Fresh-peeled
*FSu*	Harbuu	Afaan Oromoo	Astringent, sweet	Everybody	Fresh-peeled-deseeded
*FSy*	Boobboo/Odaa	Gamogna/Afaan Oromoo	Sweet	Children	Fresh-peeled-deseeded
*MK*	Qolaatii	Afaan Oromoo	Sweet	Everybody	Fresh-deseeded
*PP*	Hawuxii	Afaan Oromoo	Astringent	Everybody	Fresh-peeled
*PG*	Zayituunaa	Afaan Oromoo and Kafinoonoo	Astringent	Everybody	Fresh-whole
*RG*	Xaaxessaa	Afaan Oromoo	Astringent	Everybody	Fresh-whole
*RAb*	Goraa/qaqawwii	Afaan Oromoo	Sweet	Everybody	Fresh-deseeded
*RAp*	Baddeessaa	Afaan Oromoo	Sweet	Everybody	Fresh-whole
*RP*	Goraa	Afaan Oromoo	Sweet	Everybody	Fresh-whole
*RR*	Enjory	Amharic	Sweet	Everybody	Fresh-whole
*RS*	Goraa	Afaan Oromoo	Sweet	Everybody	Fresh-whole
*RV*	Goraa	Afaan Oromoo	Sweet	Everybody	Fresh-whole
*SM*	Mulqaa	Afaan Oromoo	Sweet	Everybody	Fresh-peeled-deseeded
*SG_g*	Baddeessaa	Afaan Oromoo	Sweet	Everybody	Fresh-deseeded
*SG_m*	Gootuu	Afaan Oromoo	Sweet	Everybody	Fresh-deseeded
*TM*	Gaashganoo	Kafinoonoo	Sweet	Everybody	Fresh-peeled
*XA*	Hudhaa/Milloo	Afaan Oromoo/Gamogna	Astringent	Everybody	Fresh-peeled-deseeded
*ZS*	Qurquraa	Afaan Oromoo	Astringent, sweet	Everybody	Fresh-deseeded

### Fruit sample pre-processing for biochemical analyses

Representative, ripe fruits in good health, suitable for human consumption, were gathered from various locations on the plants bearing edible fruits. Upon collection, the fruit samples were washed using bottled potable water. The edible portions for each fruit were extracted and placed in one or more 600 mL aluminium foil food containers with lids (Venture Team Ltd, Dunstable, UK). To maintain their freshness while in the field, these containers carrying the edible parts of the fruits were stored in a portable car battery operated Alpicool T60 freezer (Foshan Alpicool Electric Appliance Co. Ltd, Foshan, Guangdong, China), which maintained a temperature of − 10 to − 20 °C.

In order to render samples stable at ambient temperature, frozen fruit samples were blended, their fresh weights recorded and stored at − 40 °C. These samples were then subjected to freeze-drying (FreeZone 12 Litre − 84 °C Console Freeze Dryer with Stoppering Tray Dryer, Labconco Corporation, Kansas City, USA) in a set of 18 containers over a seven-day period until a constant weight was achieved. After the freeze-drying process, the weights of the dried fruit samples were recorded, and the dried fruits were vacuum-sealed in bisphenol-A free food bags using the Homeasy vacuum sealer (Homeasy Ltd, Chester, UK). Finally, the sealed, freeze-dried fruit samples were exported to the University of Nottingham, United Kingdom, for biochemical analyses. Prior to biochemical analyses, the freeze-dried fruit samples were milled using an ultra-centrifugal mill (ZM 200, Retsch GmbH, Haan, Germany) until they could pass through a 2 mm screen.

### Elemental analyses

The analyses of the elemental concentrations in the fruits and corresponding soils sampled from beneath the canopy of the fruit species was performed through the application of Inductively Coupled Plasma Mass Spectrometry (ICPMS) using a Thermo Fisher Scientific iCAP Q instrument (Thermo Fisher Scientific, Bremen, Germany). Detailed descriptions of the methodological approaches, including the analytical procedures, are provided in the subsequent sections briefly and used the standard procedures described by^6,38^.

#### Fruit samples elemental analysis

Fruit samples were prepared for ionomic analysis via a microwave digestion process, utilizing a Multiwave 5000 platform equipped with a 41HVT56 rotor with 41 vessels (Anton Paar Gmbh, Graz, Austria). Perfluoroalkoxy (PFA) digestion vessels were employed for this purpose. Initially, a finely ground fruit sample weighing 0.2 g was introduced into each PFA digestion vessel. Subsequently, 3 mL of > 68% Trace Analysis Grade (TAG) nitric acid (HNO_3_), 3 mL of Milli-Q water (18.2 MΩ cm; Fisher Scientific UK Ltd, Loughborough, UK), and 2 mL of Primar TAG hydrogen peroxide (H_2_O_2_) (Fisher Scientific UK Ltd, Loughborough, UK) were pipetted into the vessels containing the fruit samples. The digestion process was conducted under the following microwave conditions: power = 1400 W, temperature = 150 °C, pressure = 2 MPa, and a total duration of 45 min. As part of each digestion run, three operational blanks were included to account for any background contamination. The limit of detection (LOD) was determined as three times the standard deviation of analyte concentrations measured in blank samples. Elemental concentrations in the fruit samples that fell below the established LOD were excluded. Moreover, duplicate samples of a certified reference material (CRM: Wheat flour SRM 1567b, National Institute of Standards and Technology, Gaithersburg, MD, USA) were incorporated in every digestion run to ensure analytical accuracy, hence enabling determination of the percentage recovery (see Supplementary Table 2).

After completion of the digestion process, each digestion vessel was adjusted to a final volume of 20 mL by adding 12 mL of Milli-Q water. Subsequently, the contents were transferred to 25 mL universal tubes (Sarstedt Ltd., Nümbrecht, Germany) and stored at room temperature. Prior to analysis, the samples were further diluted at a 1:10 ratio with Milli-Q water in 13 mL tubes (Sarstedt Ltd., Nümbrecht, Germany). Elemental concentrations, including Ag, Al, As, B, Ba, Be, Ca, Cd, Cr, Co, Cs, Cu, Fe, K, Li, Mg, Mn, Na, Ni, P, Pb, Rb, S, Se, Sr, Ti, Tl, U, and V, were quantified using Inductively Coupled Plasma Mass Spectrometry (ICP-MS) (Thermo Fisher Scientific iCAPQ, Thermo Fisher Scientific, Bremen, Germany).

#### Soil samples elemental analysis

Soil samples, collected from beneath the canopies of fruit-bearing plants and reaching a depth of 50 cm, were composited. In Ethiopia, these soil samples underwent an initial process of air-drying and sieving to pass through a 2 mm mesh screen. Upon their arrival in the UK, the samples were further desiccated by placing them in an oven dryer at a temperature of 40 °C for a duration of three days to ensure complete dryness. Subsequently, 0.4 g subsamples of soil were placed into aqua regia digestion tubes (Sarstedt Ltd., Nümbrecht, Germany), into which 3 mL of Trace Analysis Grade (TAG) nitric acid (HNO_3_) and 2 mL of hydrogen peroxide (H_2_O_2_) (Fisher Scientific UK Ltd, Loughborough, UK) were pipetted. Glass watches were positioned on top of the digestion tubes, allowing the soil samples to soak overnight.

The following morning, an additional 9 mL of TAG hydrochloric acid (HCl) (Fisher Scientific UK Ltd, Loughborough, UK) was introduced to the solution, and the tubes were heated to a temperature of 108 °C for a duration of two hours on hot plates. Subsequently, the samples were allowed to cool for one hour. After cooling, the digestion tubes were adjusted to a final volume of 50 mL by adding 36 mL of Milli-Q water, and they were stored at room temperature. Prior to analysis, the samples were further diluted at a ratio of 1:10 with Milli-Q water in 13 mL tubes (Sarstedt Ltd., Nümbrecht, Germany). Quantification of elemental concentrations, encompassing Al, B, Ba, Ca, Cd, Co, Cr, Cs, Cu, Fe, K, Li, Mg, Mn, Na, Ni, P, Pb, Rb, S, Se, Sr, Ti, Tl, V and Zn, was conducted using Inductively Coupled Plasma Mass Spectrometry (ICP-MS) employing the Thermo Fisher Scientific iCAPQ (Thermo Fisher Scientific, Bremen, Germany).

### Assessing elemental concentration against reference nutrient intake

For essential minerals with established recommended nutrient intake (RNI), that is for calcium (Ca), iron (Fe), selenium (Se), magnesium (Mg) and zinc (Zn) we assessed the proportion of the RNI fulfilled by consuming the fruits. We used the RNI data from the Food and Agriculture Organization of the United Nations (FAO) and the World Health Organization (WHO)^39^. The RNI is a daily nutrient intake that meets the nutrient requirements of 97.5% of apparently healthy individuals within a given age and sex group^39^. Our assessment focused on determining the percentage of a specific element’s RNI met by consuming 100 g of edible parts of fresh fruits of a particular species. This analysis was based on a reference group of healthy adolescent male children, aged 10–18 years. This choice reflects the common scenario in rural Ethiopia, where male adolescents, often cattle herders, are the primary consumers of wild edible fruits. The RNIs (mg day^−1^) for this demographic are 1300 for Ca, 14.6 for Fe, 230 for Mg, 0.032 for Se, and 17.1 for Zn. We used these RNI values considering a bioavailability of 10% for Fe and the lowest bioavailability for Zn^39^.

### Statistical analysis and visualisation

To analyse the differences in elemental concentrations across different fruit species, Welch’s analysis of variance (ANOVA) test was employed. This test was used when there were three or more replicate samples for a given fruit species. The Python pingouin package was used to run Welch’s ANOVA as well as the Game-Howell post-hoc test, which allows for multiple pairwise comparisons while controlling for unequal variances and sample sizes between groups^40^.

Furthermore, to investigate the relationship between the elemental levels found in the fruits and those present in the corresponding soil samples, Pearson’s correlation analysis was conducted. The scipy package in Python was used to compute these correlation coefficients, which measure the strength and direction of the linear association between the two variables (fruit and soil elemental concentrations) across the samples. Additionally, various graphical visualizations, including bar charts, violin plots, box plots, and scatter plots, were created to represent the data using the matplotlib and seaborn packages in the Python programming language.

### Species distribution modelling

Species occurrence data sourced from the Global Biodiversity Information Facility (GBIF)^36^ and the data collected by the project “*Wild Edible Fruits (WEF) for Food and Environmental Security in Ethiopia*” were employed for modelling the distribution of 11 selected WEF across the study area based on sufficient occurrence data availability. Predictive variables for species distribution encompassed raster environmental variables obtained from Google Earth Engine (GEE)^41^ , climatic data from WorldClim^42^, and soil property data provided by the International Soil Reference and Information Centre (ISRIC)^43^.

#### Point presence data of wild edible fruits

Data on the occurrence of Wild Edible Fruit (WEF) species in Ethiopia was acquired via the GBIF Occurrences plugin in Quantum GIS (QGIS)^37^. Searches for each WEF species were conducted individually, and data were extracted specifically within the study regions of Oromia and SNNP. The downloaded data was then refined to eliminate duplicated records at identical geographic points using QGIS’s “delete duplicate geometries” feature. Field sampling records from the WEF project were also merged with the curated GBIF WEF data. In cases where merged occurrence points were less than 1500 m apart, they were displaced by 3000 m to avoid clustering. Eleven species with over 40 unique occurrence records were selected for subsequent species distribution modelling (see Fig. Sup. 1).

#### Environmental variables

For the modelling of species presence, a range of predictor variables were used, which included:

- Tree cover percentage data were obtained from the Moderate Resolution Imaging Spectroradiometer (MODIS) Terra’s Vegetation Continuous Fields (VCF) product, providing annual global coverage at 250 m resolution for 2019/20.
- Data on the predominant land cover type (LC) were sourced from the MODIS annual global land cover type product at 500 m resolution for 2021/22.
- The Enhanced Vegetation Index (EVI) was extracted from MODIS’s Combined 16-day global product at 500 m resolution for October 2022.
- Gross primary productivity (GPP) data was derived from the MODIS Aqua’s net primary productivity gap-filled annual global dataset at 500 m resolution for 2022/23.
- Climatic variables including mean annual temperature (Temp) and precipitation (Ptn), seasonality of precipitation (Ptn_SN), and precipitation during the driest (Ptn_Dry) and warmest quarters (Ptn_Warm), along with a digital elevation model (Elev), were all at a 1 km resolution. The climate data averages were from 1970 to 2000.
- Soil organic carbon (SoC) and nitrogen (N) content at various depths (15–30, 30–60, 60–100, and 100–200 cm) with a spatial resolution of 1 km.

To maintain consistency in spatial resolution, all environmental raster variables were reprojected to 1 km resolution. The resampling employed the nearest neighbour method for categorical data and the cubic spline interpolation for continuous data, using the rasterio package in Python.

#### Wild edible fruits species distribution model

The Maxent software version 3.4.4^44^ was used to model^45,46^ the distribution of the 11 WEFs. All geographic data were projected to the EPSG:32637 coordinate reference system. The point occurrence data were organised to include the geographic coordinates, and the species name so that the modelling was conducted for all species in one run. Eighty percent of the occurrence data was used for training while 20% was used for testing the model. Raster environmental predictor variables were converted from GeoTIFF to Environmental Systems Research Institute’s (ESRI) American Standard Code for Information Interchange (ASCII) grid which was the format supported by Maxent.

One of the outcomes from using the Maxent model for species distribution was a raster map indicating the presence probabilities for each of the 11 species studied, considering current environmental variables. To synthesize this information, a composite map was created that showed the areas with the highest presence probability for any of the 11 species. This was done by overlaying the individual raster maps of presence probability for each species and assigning the highest value from these layers to the consolidated map. The rasterio package in Python was employed to manage and merge the raster data for this purpose. The summary area statistics with high likelihood of presence, i.e., > = 0.8, for both individual WEF species and the 11 merged WEF species raster was calculated using the rasterio package. All map visualisations were produced using QGIS version 3.34.

## Results

Data on ethnobotany and elemental composition, along with the implications on dietary nutrition from fruits collected from 23 wild, and four cultivated plant species spanning 11 orders and 15 families, are presented in the subsequent sections. Additionally, the modelled likelihood of the 11 wild edible fruit species presence across the study area is presented. These species, detailed in Table 1, thrive across diverse Ethiopian landscapes, with altitudinal range from 1120 to 2750 m above sea level (Fig. 1 and Supplementary Table 1). Regarding their growth forms, the investigated edible fruit species comprised trees (41%), shrubs or trees (11%), shrubs (22%), lianas or shrubs (15%), and lianas or herbs (11%) as indicated in Table 1.

### Ethnobotanical data

Within the scope of this paper, the term ‘fruits’ denotes the edible, fleshy tissues derived from mature ovaries gathered from the 27 plant species included in this study. Consumption demographics revealed that the fruits were universally eaten by all age groups (93%), while 7% were being particularly favoured by children. The flavour profiles of the ripe fruits varied, with 70% described as sweet, 15% as astringent, and the remaining 15% possessing combinations of sweet, astringent, or sour tastes. The consumption methods of the fruits included eating whole and fresh (30%), whole and fresh but deseeded (30%), peeled fresh (14%), and consuming fresh after deseeding (26%), as specified in Table 2.

### Elemental concentration in edible fruits

The top five edible fruits for Ca content (mean ± std, mg per 100 g fresh weight) were *R. abyssinica* (228 ± 64), *R. glutinosa* (146 ± 54), *Z. spina-christi* (128 ± 48), *R. pinnatus* (116 ± 2), and *M. kummel* (107 ± 6). For Fe, the top five were *R. glutinosa* (3.57 ± 1.49)*, S. guineense ssp. guineense* (2.41 ± 3.31)*, R. steudneri* (1.95 ± 1.32)*, C. africana* (1.68 ± 1.15)*,* and *R. volkensii* (1.65 ± 0.88). Magnesium concentration was highest in *R*. *pinnatus* (76 ± 2), *T*. *mauritianum* (72 ± 0), *R*. *steudneri* (64 ± 8), *R*. *abyssinica* (60 ± 7), and *R*. *apetalus* (57 ± 7). Selenium concentration peaked with *S*. *mitis* (0.014 ± 0), with *C*. *monoica* and *C*. *myxa* (both 0.006 ± 0), *M*. *kummel* (0.004 ± 0.004) and *P*. *guajava* (0.003 ± 0.004) followed closely. While *T*. *mauritianum* (1.132 ± 0), *R*. *pinnatus* (0.489 ± 0.01), *R*. *rosifolius* (0.453 ± 0.105), *C*. *myxa* (0.449 ± 0.004), and *C*. *spinarum* (0.433 ± 0.236) were the top five for zinc content. More detailed statistics for these and other species can be found in Supplementary Table 3. Fig. Sup. 2 presents box plots of the individual elemental concentrations for the 27 edible fruit species, with comparisons to the concentrations in strawberries where data is available.

**Fig. 3:**
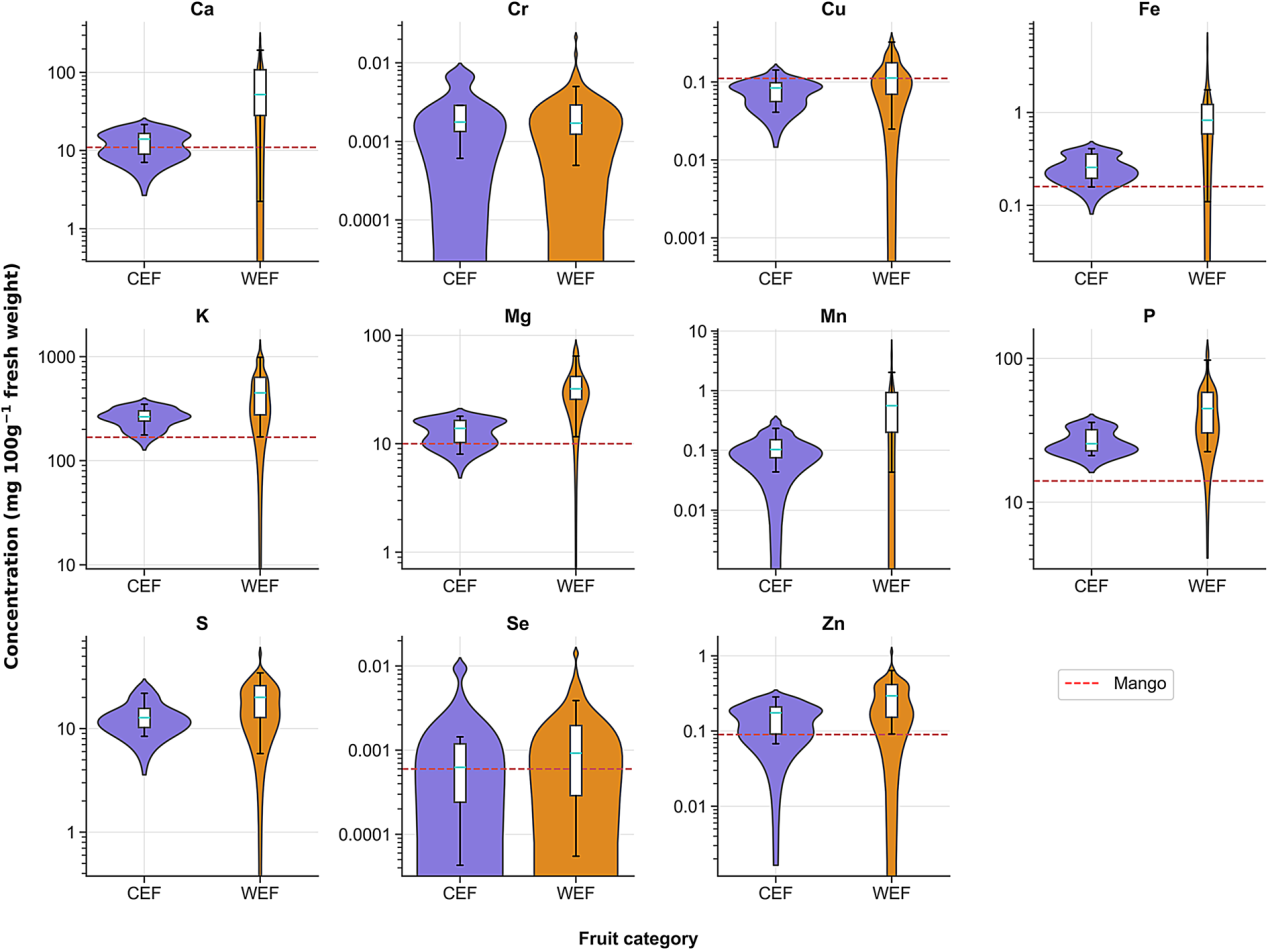
Elemental concentrations (log_10_ scale y-axis) across 27 edible fruit bearing species categorised as cultivated (CEF, n = 36), and wild (WEF, n = 64). Each panel depicts the elements. Medians (turquoise lines in the overlaid box plots) for each category are benchmarked against mango elemental concentration values (red dashed lines^47^).

Edible fruits were categorized into two groups for comparison of their essential mineral concentrations: cultivated (CEF) and wild (WEF). The CEF was comprised of *A*. *cherimola*,* C*. *edulis*, *D*. *caffra* and *P*. *guajava*, while WEF consisted of 23 species (refer to Table 1 for the lists). It is important to highlight that while *D. caffra* is primarily cultivated for use as a live fence or hedge, the fruits are sometimes consumed on an occasional basis by certain individuals, despite not being the main purpose for growing this plant species. Results demonstrated that the median concentrations of essential minerals were greater in WEFs versus CEFs for all elements analysed except Cr (Fig. 3). Furthermore, the mineral content was larger among WEFs compared to concentrations reported for commercially grown mango (Fig. 3). The comparison of mean elemental contents of the different fruit species for selected essential elements is presented in Supplementary Table 4.

### Association between fruit and soil elemental concentrations

**Fig. 4:**
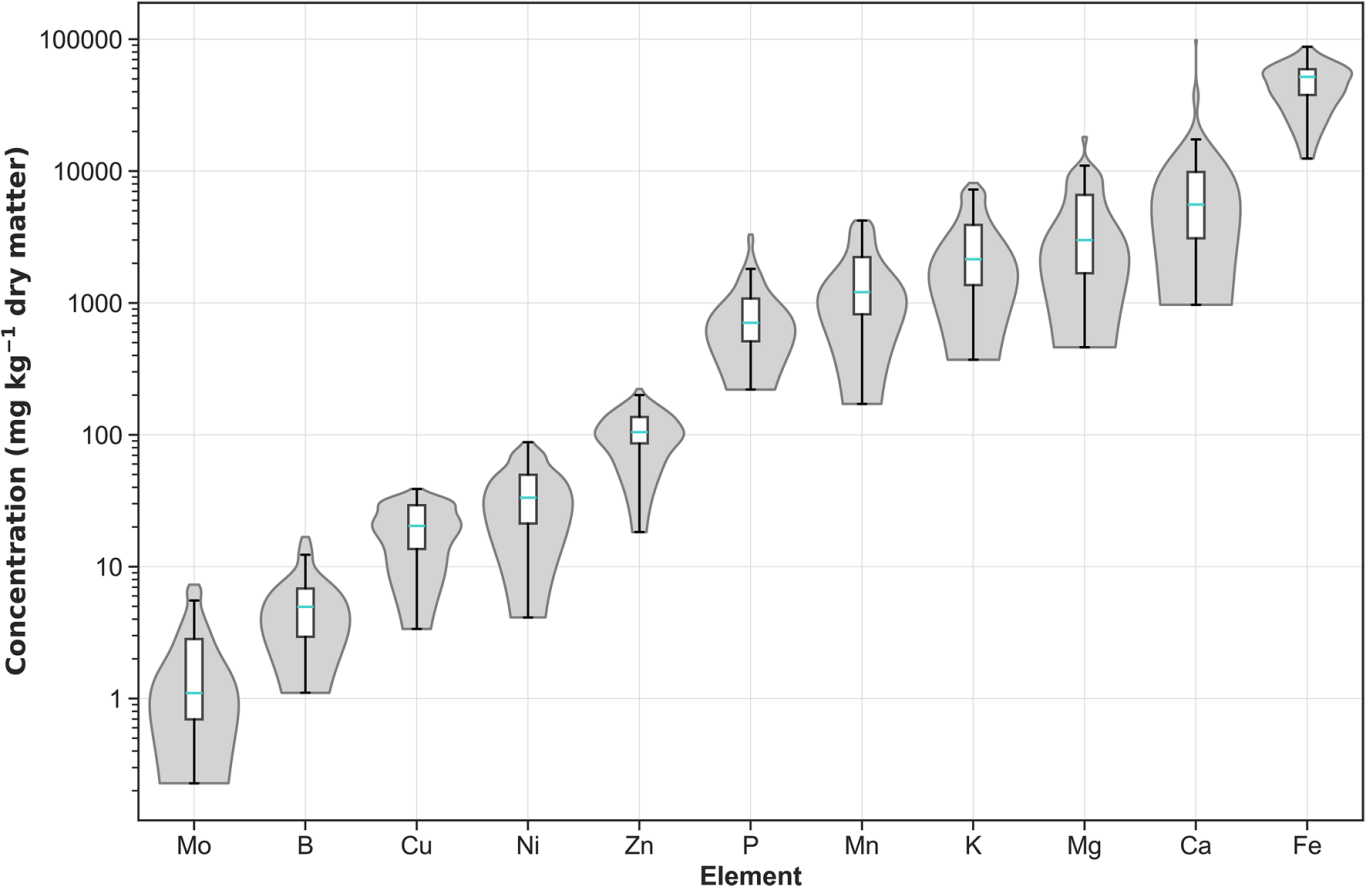
Concentration of elements (log_10_ scale y-axis) in soils (n = 53) sampled beneath the canopy of plants bearing edible fruits. The turquoise horizontal lines in the overlaid box plots represent median values.

The range of plant-essential elements concentrations found in soil samples taken from beneath the canopies of edible fruit-bearing plants are presented in Fig. 4. The levels of these elements varied widely, with Mo displaying the smallest concentration (mean ± std) at 0.08 ± 0.05, and Fe exhibiting the largest at 49,012 ± 16,960 mg kg^−1^ of dry matter. Detailed data can be found in Supplementary Table 1 and descriptive statistics can be seen in Supplementary Table 3.

**Fig. 5:**
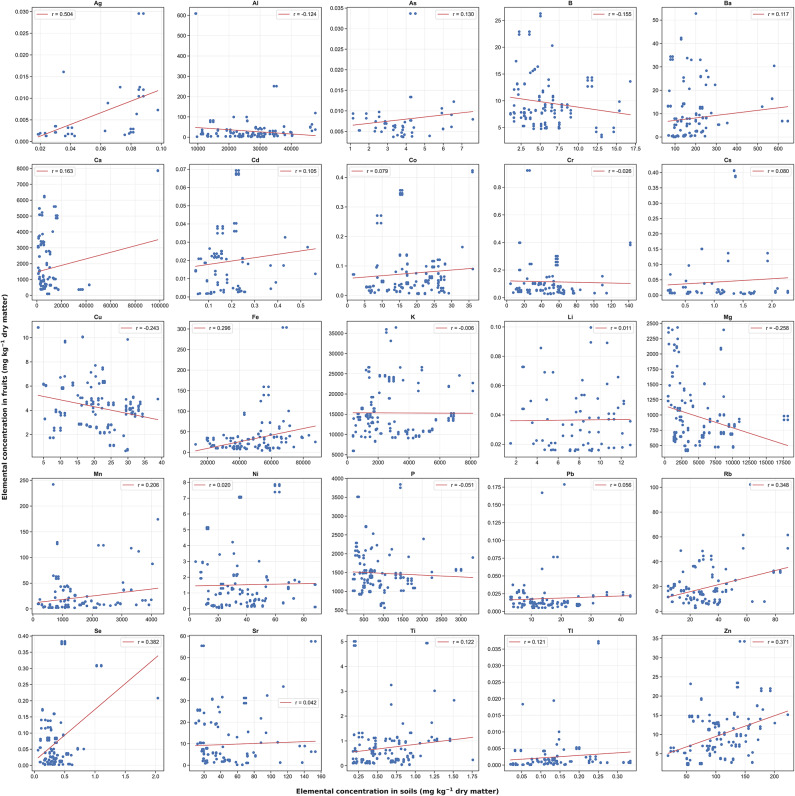
A scatter plot of the concentration of elements in fruits and the respective soils. Each panel represents a different element and includes a linear regression line, with the correlation coefficient denoted by ‘r’ for each element’s panel.

The relationship between elemental concentrations in fruits and their corresponding soils was characterized by generally weak correlations. The correlation coefficients varied from a low of -0.26 for Mg to a high of 0.5 for Ag as detailed in Fig. 5. Notably, significant correlations were observed for elements essential to human health, including Ca (r = 0.16, *p* = 0.042), Cu (r = − 0.24, *p* =  0.002), Fe (r = 0.3, *p*  =  0.000), Mn (r = 0.21, *p* = 0.010), Se (r = 0.38, *p* = 0.000), and Zn (r = 0.37, *p* = 0.000) (see Supplementary Table 3).

### Contribution to dietary mineral intake by wild edible fruits

**Fig. 6:**
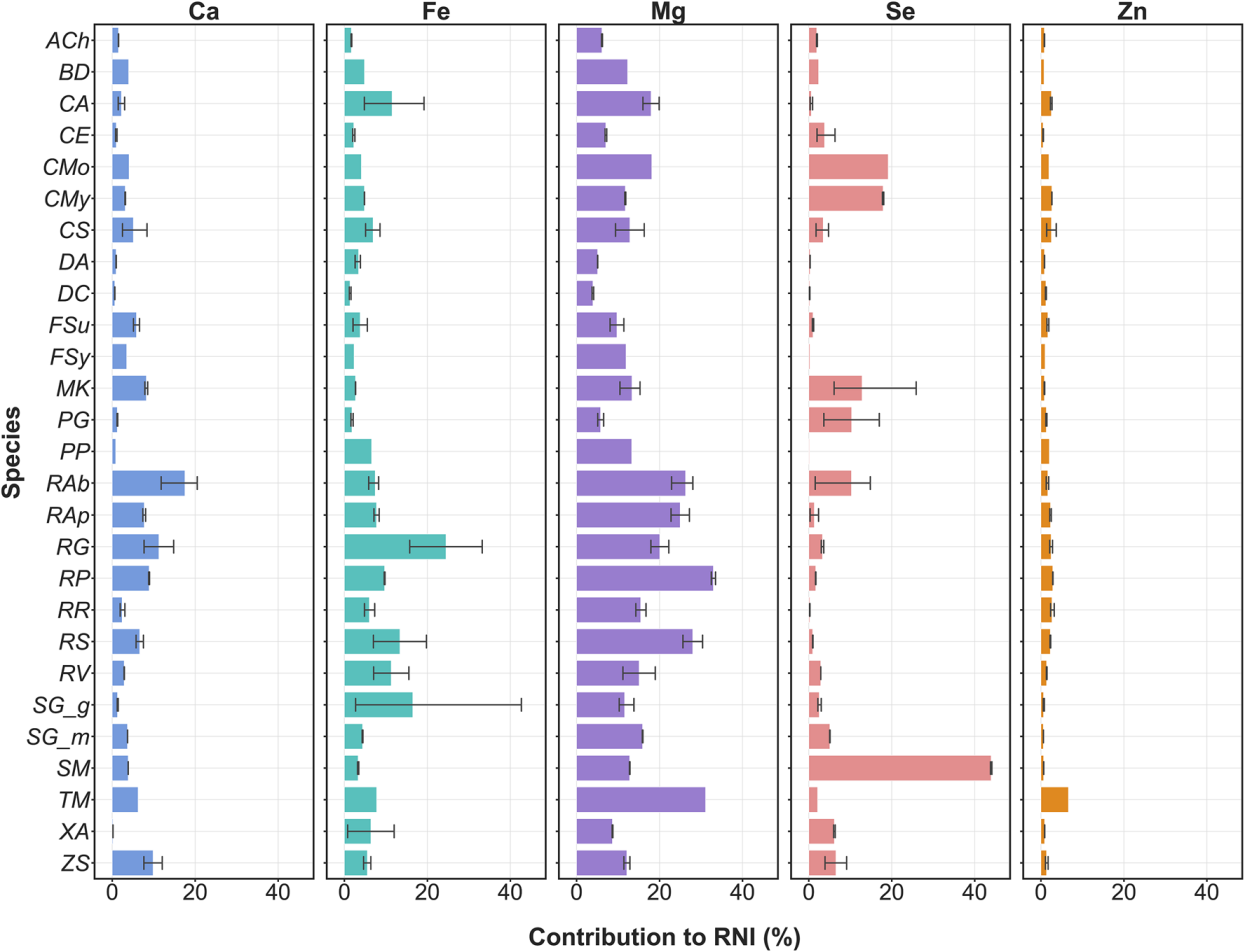
Percentage of Recommended Nutrient Intake (RNI) for boys aged 10–18 provided by 100 g of different fresh fruits. Minerals include Ca, Fe, Mg, Se, and Zn shown in separate panels. Error bars show the 95% confidence interval. Refer to Table 1 for scientific names of the species acronyms (y-axis).

The mineral intake contributed by the fruits shows variability contingent on the type of element and the specific fruit species, as indicated in Fig. 6. For boys aged 10–18 years, a 100-g serving of fresh fruits from *R. abyssinica* (*RAb*), *R. glutinosa (RG), R. pinnatus* (*RP), S. mitis* (*SM),* and *T. mauritianum* (TM) has the potential to provide up to 20%, 43%, 33%, 44%, and 7% of their daily recommended nutrient intake for Ca, Fe, Mg, Se, and Zn, respectively (Fig. 6).

### Wild edible fruits species distribution

**Fig. 7:**
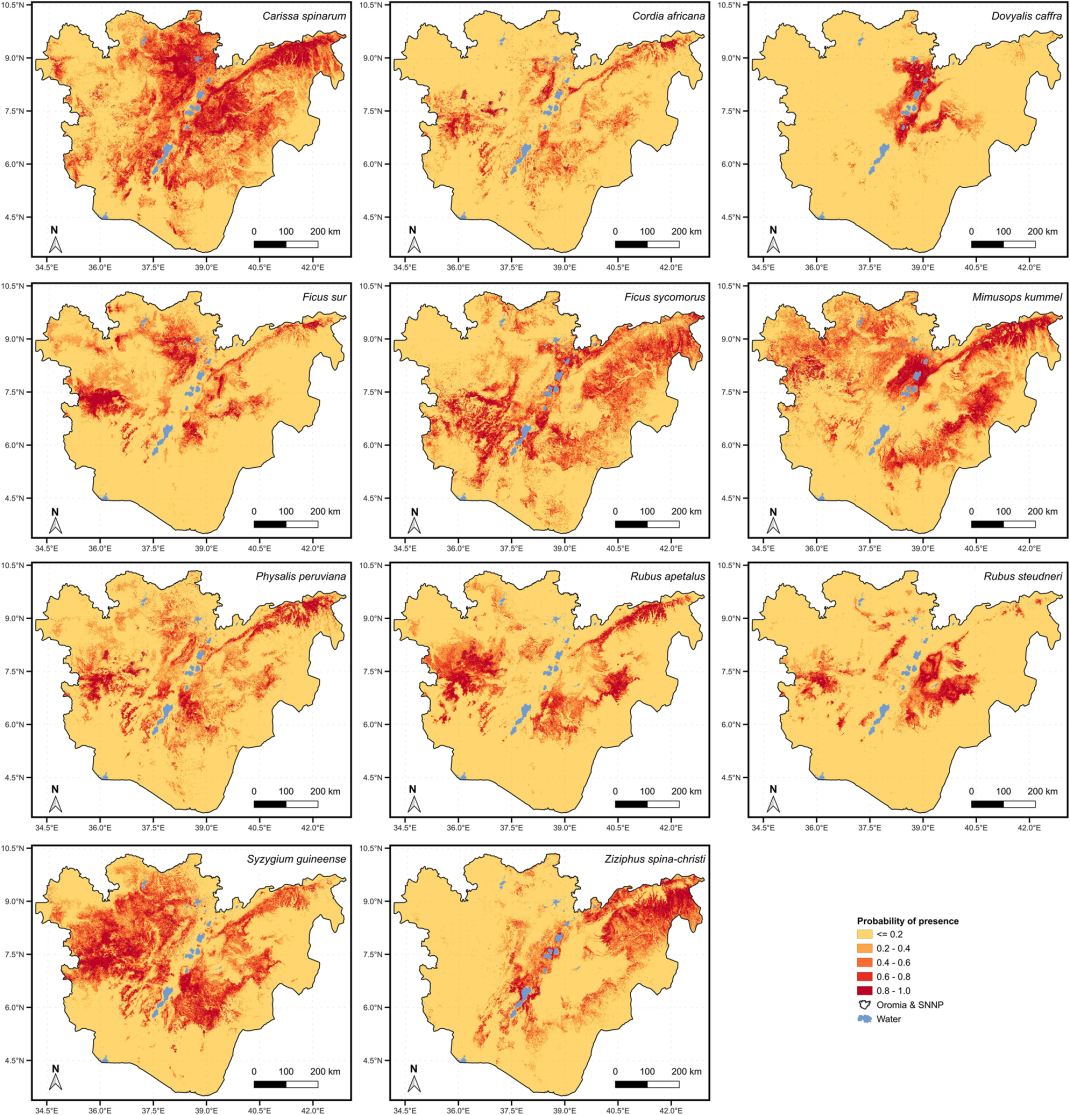
Presence likelihood for *Carissa spinarum, Cordia africana, Doviyalis caffra, Ficus sur, Ficus sycomorus, Mimusops kummel, Physalis peruviana, Rubus apetalus, Rubus steudneri, Syzigium guineense* and *Ziziphus spina-christi* across the Oromia and SNNP regions of Ethiopia.

**Fig. 8:**
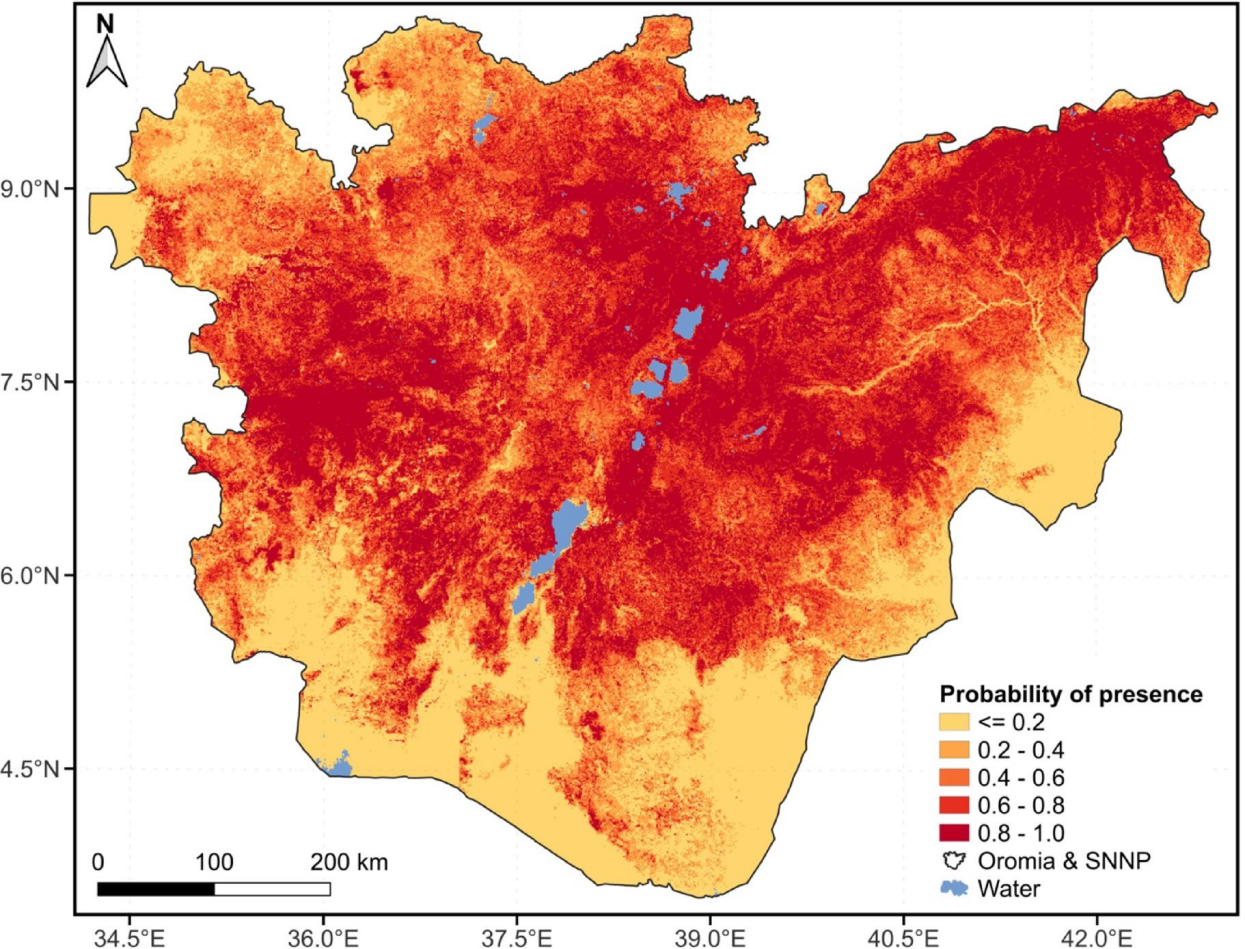
Merged maximum likelihood of presence for *Carissa spinarum, Cordia africana, Doviyalis caffra, Ficus sur, Ficus sycomorus, Mimusops kummel, Physalis peruviana, Rubus apetalus, Rubus steudneri, Syzigium guineense* and *Ziziphus spina-christi* across the Oromia and SNNP regions of Ethiopia.

The Maxent model indicated substantial spatial variation in the presence of wild edible fruit species across the 433,950 km^2^ study area (Figs. 7 and 8). The results indicate that 42.47% of the study area had a high likelihood of presence (probability ≥ 0.8) for at least one of the 11 species considered. When considering the maximum probability of presence across all species, 26.64% of the study area had high suitability indicating the presence of more than one species at a given locality. Among the individual species, *C. spinarum, S. guineense,* and *M. kummel* exhibited widespread presence (Fig. 7), with 7.91%, 6.11%, and 5.42% of the study area, respectively, having high suitability for these species. The log of Maxent model prediction and parameters, measures of model performance can be found in the Supplementary file 1.

## Discussion

This study provides the first comprehensive assessment of the elemental composition of 23 wild and four cultivated edible fruits found across diverse agroecological zones in southern parts of Ethiopia. The findings reveal that several wild edible fruit (WEF) species are good sources of essential minerals, often surpassing the levels found in cultivated edible fruits (CEFs) and commercially grown fruits like avocados, blackberries, mangoes, oranges, papayas, and strawberries. When comparing our elemental concentration findings with the USDA FoodData Central database^47^, readers should note that wild edible fruits and commercial varieties differ fundamentally. These differences include genetic variations, growth in distinct soil and environmental conditions across different geographic regions, and varying fruit moisture contents between the two categories. Notably, *C. africana, C. myxa, C. spinarum, R. abyssinica, R. apetalus, R. glutinosa, R. pinnatus, R. rosifolius, R. steudneri, R. volkensii, S. mitis, T. mauritianum,* and *S. guineense ssp. guineense* emerged as good sources of Ca, Fe, Mg or Se. They have the potential to contribute significantly to the daily recommended nutrient intake for these minerals, especially for vulnerable groups like adolescent boys. The high Se content in *S. mitis* fruits, reaching 0.31 mg kg^−1^ dry matter, surpasses the levels reported for baobab fruits (0.169 mg kg^−1^ dry matter) and lower than that of *Moringa oleifera* immature pods (1.99 mg kg^−1^ dry matter)^48^, suggesting that this species may possess exceptionally efficient mechanisms for accumulating and storing Se from its environment.

Among commercial fruits, oranges have the largest Ca content (42 mg per 100 g fresh fruit), but 15 of the wild edible fruit (WEF) species contain even higher levels of Ca than oranges. Blackberries have the highest Fe content (0.62 mg per 100 g fresh fruit) among commercial fruits, but 17 WEF species surpass blackberries in Fe concentration. Avocados have the highest Mg content (29 mg per 100 g fresh fruit) in commercial fruits, but 15 WEF species exceed avocados in Mg levels. Papayas have the highest Se content (0.6 μg per 100 g fresh fruit) among commercial fruits, while 16 WEF species contain more Se than papayas. Avocados have the highest Zn content (0.64 mg per 100 g fresh fruit) among commercial fruits, but the WEF species *T. mauritianum* has twice the Zn concentration of avocados (refer to Table 3^47^, and Supplementary Table 3).

**Table 3:** The concentrations of various elements found in commercial fruits^47^. The concentrations are expressed in mg for all elements, except for Se, which is measured in µg per 100 g of edible parts of fresh fruit.

Fruit	Elemental concentration							
	Ca	Cu	Fe	K	Mg	P	Se	Zn
Apple	5	0.03	0.03	104	5	9	0	0.02
Apricot	13	0.078	0.39	259	10	23	0.1	0.2
Avocado	12	0.19	0.55	485	29	52	0.4	0.64
Blackberries	29	0.165	0.62	162	20	22	0.4	0.53
Blueberries	6	0.057	0.28	77	6	12	0.1	0.16
Kiwi fruit	35	0.134	0.24	198	16	34	0.2	0.14
Mango	11	0.111	0.16	168	10	14	0.6	0.09
Orange	42	0.054	0.22	174	10	18	0.2	0.09
Papaya	20	0.045	0.25	182	21	10	0.6	0.08
Pear	9	0.077	0.18	104	7	11	0.1	0.09
Plum	6	0.057	0.17	157	7	16	0	0.1
Starfruit	3	0.137	0.08	133	10	12	0.6	0.12
Strawberries	12	0.119	0.28	89	12	20	0.4	0.18
Watermelon	7	0.042	0.24	112	10	11	0.4	0.1

The concentrations of some essential minerals in the WEFs were correlated with elemental levels in the soils where they grow, suggesting soil fertility could be leveraged to increase the micronutrient content in these species. However, the generally weak correlation indicates that factors beyond soil composition, such as plant genetic traits and other environmental conditions, play a role in determining mineral uptake and accumulation in edible fruits, corroborating previous research^49–51^. Further research should explore the relative influences of genotype and environment to better understand fruit nutrient biofortification potential.

Our data demonstrate that reasonable serving sizes of WEF species like *R. abyssinica* and *R. glutinosa* could supply up to 40% of daily recommended intakes of essential minerals like calcium and iron for Ethiopian adolescent boys. Complementing staple cereal intake with these fruits can help address widespread mineral nutritional deficiencies^7,8,52^ in rural communities where access to commercial fruits are very low. As wild harvests are unlikely to meet national-scale demand, integrating native fruit trees into smallholder farms via agroforestry could boost both access and conservation. The diverse growth forms of the studied WEFs, ranging from trees and shrubs to lianas and herbs, present opportunities for their integration into various agroforestry systems and traditional home gardens, promoting both nutritional security and biodiversity conservation. The universal consumption of these fruits across age groups and their varied flavour profiles indicate their potential to be embraced by local communities as part of their dietary traditions. However, further research is needed on propagation, cultivation requirements, and fruit production potential.

The substantial spatial variation observed in the distribution of WEF species across Ethiopia’s landscapes highlights the need for location-specific strategies to promote their utilization and conservation. The extensive prevalence of species like *C. spinarum*, *S. guineense*, and *M. kummel* suggests that these fruits could be prioritized in consultation with relevant stakeholders for incorporation into local diets and agricultural systems in areas where they are abundant. Interventions should include training programs for Health Extension Workers and Agriculture Extension Workers regarding indigenous fruit availability and nutritional composition profiles.

This study reinforces the need to valorise and conserve these underutilized resources by highlighting their nutritional value. Integrating WEFs into dietary recommendations, agricultural practices, and environmental policies could contribute to addressing dietary mineral deficiencies, improving food and nutritional security, and preserving Ethiopia’s rich plant biodiversity.

However, further research is warranted to investigate factors influencing the bioavailability of these essential minerals from WEF species, as well as to assess their other potential nutritional, anti-nutritional and bioactive components. This study lays the foundation to promote the use and conservation of little-known but nutritionally and ecologically important native fruits. Our documentation of the ethnobotanical knowledge, uses and chemistry of these species can guide utilization campaigns, inform dietary recommendations, and support biodiversity policy in Ethiopia. Public education, engagement with local communities and policy makers will be key next steps to realize the potential of these overlooked resources to enhance food and nutrition security. Overall, this research supports an integrated socioecological approach leveraging biodiversity and traditional knowledge to sustainably address malnutrition while protecting invaluable genetic resources for future generations.

In conclusion, this study underscores the potential of WEF species as nutritious and readily available resources that could play a vital role in enhancing dietary diversity and alleviating mineral deficiencies in Ethiopia. By fostering the sustainable utilization and conservation of these underutilized plant resources, Ethiopia can simultaneously advance its goals of improving food and nutritional security while preserving its rich biodiversity.

## Supplementary Information

Below is the link to the electronic supplementary material.

Supplementary Material 1

Supplementary Material 2

Supplementary Material 3

Supplementary Material 10

Supplementary Material 5

Supplementary Material 6

Supplementary Material 7

Supplementary Material 8

Supplementary Material 9

Supplementary Material 10

Supplementary Material 11

## Data Availability

Data is provided within the manuscript or supplementary information files. Additionally, the data is publicly available at the following link on Figshare. 10.6084/m9.figshare.25869115.
