# Supplementary material for: Elemental concentration and spatial distribution of wild edible fruits and implications for dietary mineral intake in Ethiopia
Source: Sci Rep. 2025 Nov 27;15:42307. doi: 10.1038/s41598-025-26400-7 (PMC12661052; doi:10.1038/s41598-025-26400-7)
Supplement: Supplementary file 1 — Supplementary Material 1 [file 41598_2025_26400_MOESM1_ESM.docx]

# Appendices

Appendix 1. A questionnaire used to collect ethnobotanical data concerning the wild edible fruits growing in the Oromia and SNNP regions Ethiopia.

Appendix 2. A questionnaire used to collect the metadata for fruit and soil samples sampled in the Oromia and SNNP regions of Ethiopia.
