# Supplementary material for: Elemental concentration and spatial distribution of wild edible fruits and implications for dietary mineral intake in Ethiopia
Source: Sci Rep. 2025 Nov 27;15:42307. doi: 10.1038/s41598-025-26400-7 (PMC12661052; doi:10.1038/s41598-025-26400-7)
Supplement: Supplementary file 2 — Supplementary Material 2 [file 41598_2025_26400_MOESM2_ESM.pdf]

WEF-ethnobotany

Wild Edible Fruits

WEF

Fruit sample ID *

Please scan the QR-Code from the label that was pasted on to the fruit sample container.

Participant information sheet and consent to be read out.

University of Nottingham
UK | CHINA | MALAYSIA
School of Biosciences

Information for participant

Name of Study: Wild edible fruits (WEF) for food and environmental security in Ethiopia
Name of Researcher(s): Dr Diriba B Kumssa, Dr Tristan Dew, Dr Kassahun Tesfaye, Mr Obssi Dessalegn, Prof Levi Yant, Prof Martin Broadley
Lead researcher: Dr Diriba B Kumssa (diriba.kumssa1@nottingham.ac.uk)

Invitation
Hello, my name is _______. I am a member of the WEF project team from the University of Nottingham, UK. You are being invited to be involved in a research study. Before you decide whether you want to take part, it is important for you to understand why the research is being done and what your participation will involve. Please listen to the following information carefully and discuss it with other people if you wish. Please let me know if anything is unclear or if you would like more information. Take time to decide whether you wish to take part.

What is the purpose of this study? The aim of this study is to collect information about the dietary usage and other related purposes of the WEF growing in your vicinity.

Why I have been chosen? You are randomly selected because you live close to a WEF growing in this area. There is no specific reason other than this.

What will participation involve? You will be asked questions about the WEF how you use or manage it. Your responses will be written down on a tablet (please show the tablet for the participant and the picture of the WEF which was sampled earlier in this vicinity).

What if I decide that I don't want to take part? You are free to decide that you don't want to take part in the study and can:
1. Refuse to answer any questions that you don't want to
2. Decide to stop the interview at any time
3. Remove your consent for the data collected to be used

Will I be paid for my time? There is no payment for taking part in this survey.

Will I be anonymous, and who will know my identity? If you agree to take part in the interview, a Participant Number will be generated for you, and that's the only thing that will be used to identify you. Your identity will only be known by the interviewer, and will not be found in any record. Hard copy and electronic data will be stored on the University of Nottingham's computer network: this will be deleted after 5 years, or if you withdraw your consent (whichever is sooner).

Who shall I contact with any questions? Please contact the Principal Investigator, Dr Diriba B Kumssa (diriba.kumssa1@nottingham.ac.uk). If you have any concerns about the research, then in the first instance please contact: Dr Kate Millar (kate.millar@nottingham.ac.uk)

Participant Consent Form

I confirm that:	Checked
I have read and understood the Participant Information Sheet, or it has been read to me. I have been able to ask questions about the study and my questions have been answered to my satisfaction.	X
I consent voluntarily to be a participant in this study and understand that I can refuse to answer questions and I can withdraw from the study at any time, without having to give a reason.	X
Taking part in this study involves an interview.	X
My words can be quoted in publications, reports, web pages and other research outputs.	X
I give permission for the de-identified (anonymised) data that I provide to be used for future research and learning.	X

I agree to take part in the study.

I have understood why this research is conducted and agree to take part in the study. *

○ OK

Signature to indicate consent

Respondent's gender

- Female
- Male
- Other

Respondent's age range (yrs)

- 18–24 years old
- 25–34 years old
- 35–44 years old
- 45–64 years old
- 65–74 years old
- 75 years or older

What is the local name of the fruit bearing woody plant? *

In what language is this local name? *

What is the growth form of the woody plant?

- [ ] Tree
- [ ] Shrub
- [ ] Liana

When does the woody plant fruit? *

[ ] January	[ ] February	[ ] March
[ ] April	[ ] May	[ ] June
[ ] July	[ ] August	[ ] September
[ ] October	[ ] November	[ ] December

Who consumes the fresh fruits? *

- [ ] Children (girls)
- [ ] Adult women
- [ ] Everybody
- [ ] Adolescent women
- [ ] Everybody during lean season
- [ ] Children (boys)
- [ ] Adolescent men
- [ ] Adult men
- [ ] Nobody

What is the taste of the fresh fruit when it is ripe? *

- [ ] Sweet
- [ ] Sour
- [ ] Astringent
- [ ] Other

What is the colour of the ripe fruit? *

- [ ] Black

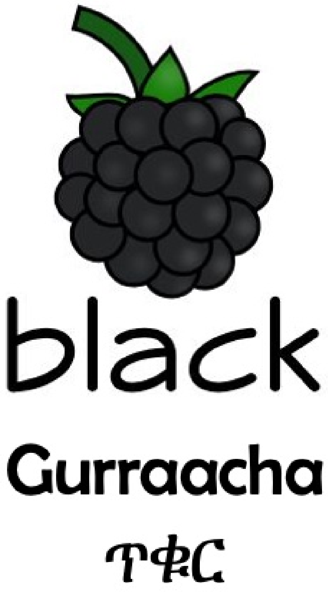

- [ ] Blue

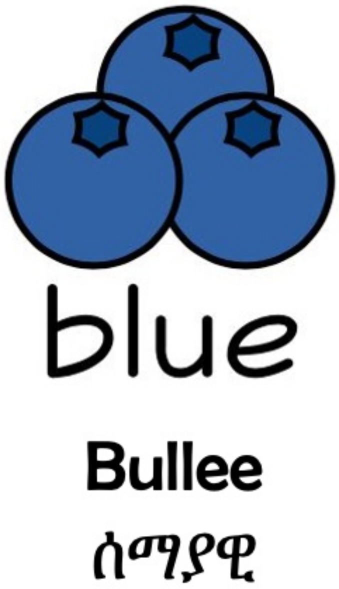

- [ ] Brown

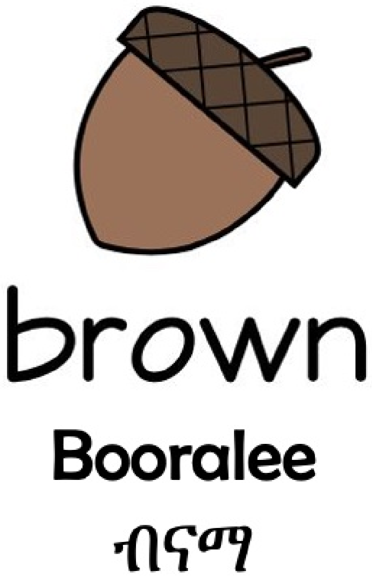

☐ Gray

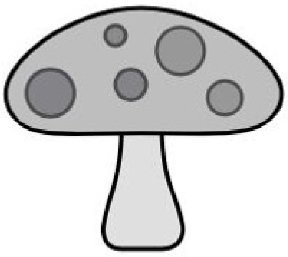

gray

Daalacha

ግራጫ

☐ Green

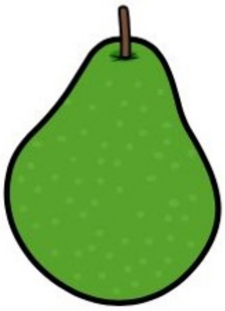

green

Magariisa

አረንጓዴ

☐ Orange

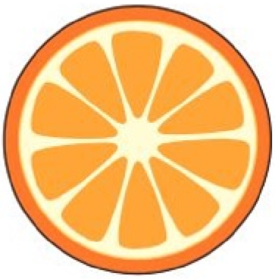

orange

Burtukaana

ብርቱካናማ

☐ Peach

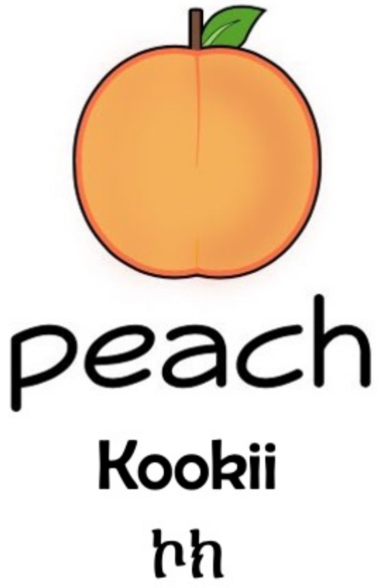

☐ Pink

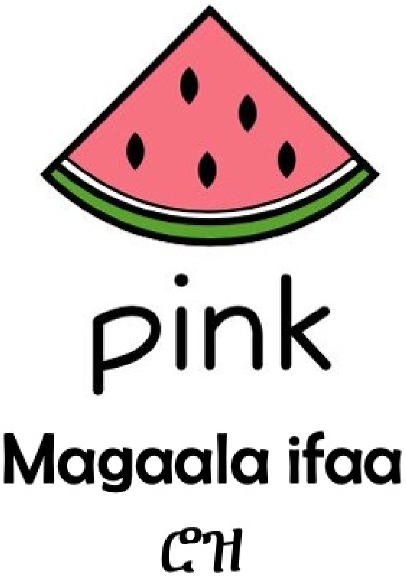

☐ Purple

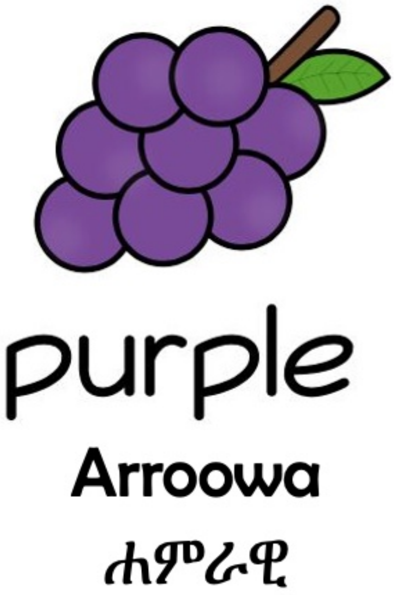

☐ Red

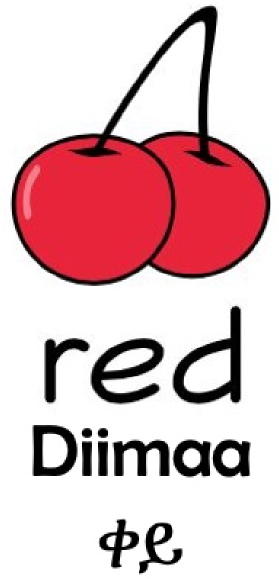

- [ ] Yellow

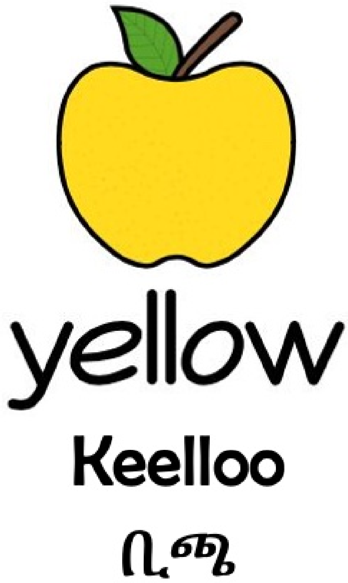

- [ ] White

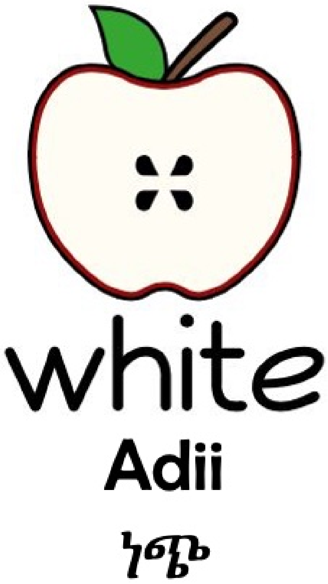

- [ ] Other

How is the fruit consumed? *

- [ ] Whole fresh
- [ ] Peeled fresh
- [ ] Deseeded fresh
- [ ] Whole cooked
- [ ] Peeled cooked
- [ ] Deseeded cooked
- [ ] Fermented
