# Supplementary material for: Elemental concentration and spatial distribution of wild edible fruits and implications for dietary mineral intake in Ethiopia
Source: Sci Rep. 2025 Nov 27;15:42307. doi: 10.1038/s41598-025-26400-7 (PMC12661052; doi:10.1038/s41598-025-26400-7)
Supplement: Supplementary file 3 — Supplementary Material 3 [file 41598_2025_26400_MOESM3_ESM.pdf]

WEF-Sample metadata

This note can be read out loud

Wild Edible Fruits

WEF

Location of the sampled wild woody plant edible fruit. *

latitude (x.y °)

longitude (x.y °)

altitude (m)

accuracy (m)

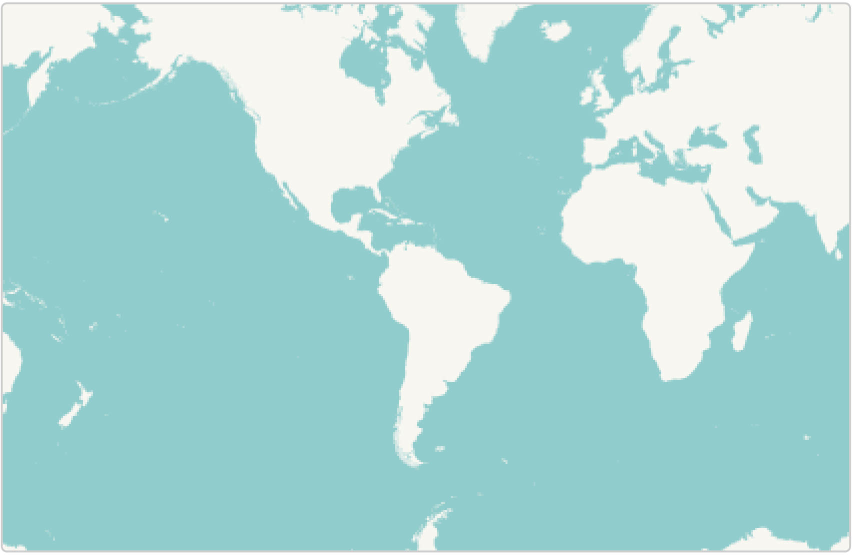

Scientific name of the woody plant *

Select one from the list. If this is not in the list choose "Unidentified".

- Annona cherimola
- Annona senegalensis
- Balanites aegyptiaca
- Berchemia discolor
- Bridelia micrantha
- Bridelia scleroneura
- Afrocanthium lactescens
- Carissa spinarum
- Celtis africana
- Clausena anisata
- Cordia africana
- Diospyros mespiliformis
- Dovyalis abyssinica
- Ehretia cymosa
- Euclea divinorum
- Ficus palmata
- Ficus sur
- Ficus sycomorus
- Ficus vasta
- Flacourtia indica
- Flueggea virosa
- Gardenia ternifolia
- Grewia ferruginea
- Lantana viburnoides
- Mimusops kummel
- Myrsine africana
- Olea europaea
- Opuntia ficus-indica
- Osyris quadripartita
- Pappea capensis
- Phoenix reclinata
- Piliostigma thonningii
- Protea gaguedi
- Searsia retinorrhoea
- Searsia tenuinervis

- Searsia tenuinervis
- Searsia pyroides
- Rosa abyssinica
- Rubus apetalus
- Sclerocarya birrea
- Sterculia africana
- Strychnos innocua
- Syzygium guineense
- Tamarindus indica
- Vitex doniana
- Ximenia americana
- Ximenia caffra
- Ziziphus abyssinica
- Ziziphus mucronata
- Ziziphus spina-christi
- Lannea schimperi
- Lantana camara
- Prosopis juliflora
- Premna resinosa
- Pouteria altissima
- Phyllanthus limmuensis
- Pavetta crassipes
- Pavetta abyssinica
- Oncoba spinosa
- Olea capensis
- Afromorus mesozygia
- Mimusops laurifolia
- Ocimum grandiflorum
- Balanites rotundifolia
- Allophylus ferrugineus
- Allophylus abyssinicus
- Vachellia tortilis
- Acokanthera schimperi
- Morus alba
- Embelia schimperi
- Elaeodendron buchananii
- Ekebergia capensis

[illegible]
- Dombeya torrida
- Diospyros abyssinica
- Dombeya longibracteolata
- Crateva adansonii
- Cordia sinensis
- Cordia monoica
- Commiphora edulis
- Commiphora africana
- Euclea racemosa
- Erythrococca abyssinica
- Rhaphiolepis loquata
- Cephalopentandra ecirrhosa
- Capparis tomentosa
- Celtis toka
- Capparis erythrocarpos
- Bullockia pseudosetiflora
- Capparis decidua
- Buddleja polystachya
- Boscia senegalensis
- Boscia coriacea
- Lannea schweinfurthii
- Hyphaene thebaica
- Hoslundia opposita
- Heliotropium steudneri
- Grewia flavescens
- Grewia villosa
- Grewia velutina
- Grewia trichocarpa
- Grewia tenax
- Grewia schweinfurthii
- Grewia mollis
- Grewia kakothamnos
- Grewia erythraea
- Grewia bicolor
- Grewia arborea
- Grewia balensis

- ◯ Garcinia livingstonei
- ◯ Garcinia ovalifolia
- ◯ Ficus vallis-choudae
- ◯ Ficus platyphylla
- ◯ Ficus ovata
- ◯ Ficus ingens
- ◯ Rytigynia neglecta
- ◯ Rubus volkensii
- ◯ Rubus steudneri
- ◯ Ritchiea albersii
- ◯ Rubus erlangeri
- ◯ Psydrax schimperiana
- ◯ Pygeum africanum
- ◯ Ziziphus mauritiana
- ◯ Vangueria madagascariensis
- ◯ Vangueria apiculata
- ◯ Uvaria leptocladon
- ◯ Zanthoxylum asiaticum
- ◯ Uvaria angolensis
- ◯ Coptosperma graveolens
- ◯ Solanum memphiticum
- ◯ Spiniluma oxyacantha
- ◯ Scutia myrtina
- ◯ Salvadora persica
- ◯ Scolopia theifolia
- ◯ Sageretia thea
- ◯ Saba comorensis
- ◯ Maerua subcordata
- ◯ Manilkara butugi
- ◯ Lepisanthes senegalensis
- ◯ Unidentified

Fruit sample ID_1 *

Please scan the QR-Code from the label that was pasted on to the fruit sample container.

....

Fruit sample ID_2

Please scan the QR-Code from the label that was pasted on to the fruit sample container.

Fruit sample ID_3

Please scan the QR-Code from the label that was pasted on to the fruit sample container.

Soil sample ID *

Please scan the QR-Code from the label that was pasted on to the soil sample bag.

DNA sample ID_1 *

Please scan the QR-Code from the label that was pasted on to the DNA sample bag.

DNA sample ID_2 *

Please scan the QR-Code from the label that was pasted on to the DNA sample bag.

Surrounding photo

Picture of the surrounding area

Click here to upload file. (< 5MB)

Plant photo

Picture of the whole woody plant

Click here to upload file. (< 5MB)

Leaves photo

Picture of the leaves

Click here to upload file. (< 5MB)

Fruits photo
Picture of the fruits on the tree

Click here to upload file. (< 5MB)

Flowers photo
Picture of the flowers

Click here to upload file. (< 5MB)

Fruit sample photo
Picture of the collected fruit samples

Click here to upload file. (< 5MB)

Fruit sample boxes photo *
Take the picture of the fruit sample boxes from a site.

Click here to upload file. (< 5MB)

Soil sample bag photo *
Take the picture of the soil sample bag from a site.

Click here to upload file. (< 5MB)

DNA sample bags photo *
Take the picture of the DNA sample bags from a site.

Click here to upload file. (< 5MB)
