# Supplementary figures and images for: Elemental concentration and spatial distribution of wild edible fruits and implications for dietary mineral intake in Ethiopia (part 1 of 2)
Source: Sci Rep. 2025 Nov 27;15:42307. doi: 10.1038/s41598-025-26400-7 (PMC12661052; doi:10.1038/s41598-025-26400-7)

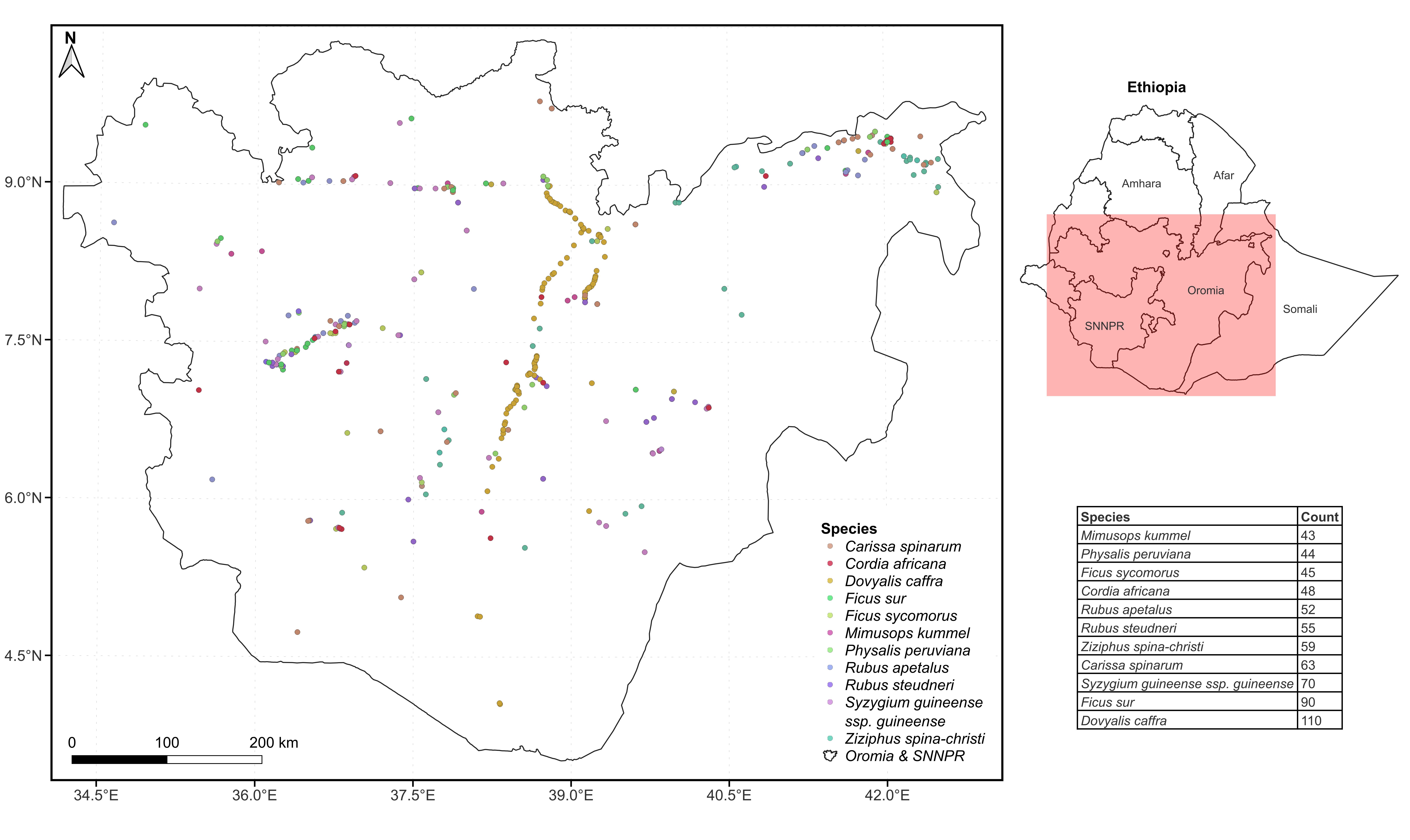

Supplement: Supplementary file 4 — Supplementary Material 10 [file 41598_2025_26400_MOESM4_ESM.jpeg]

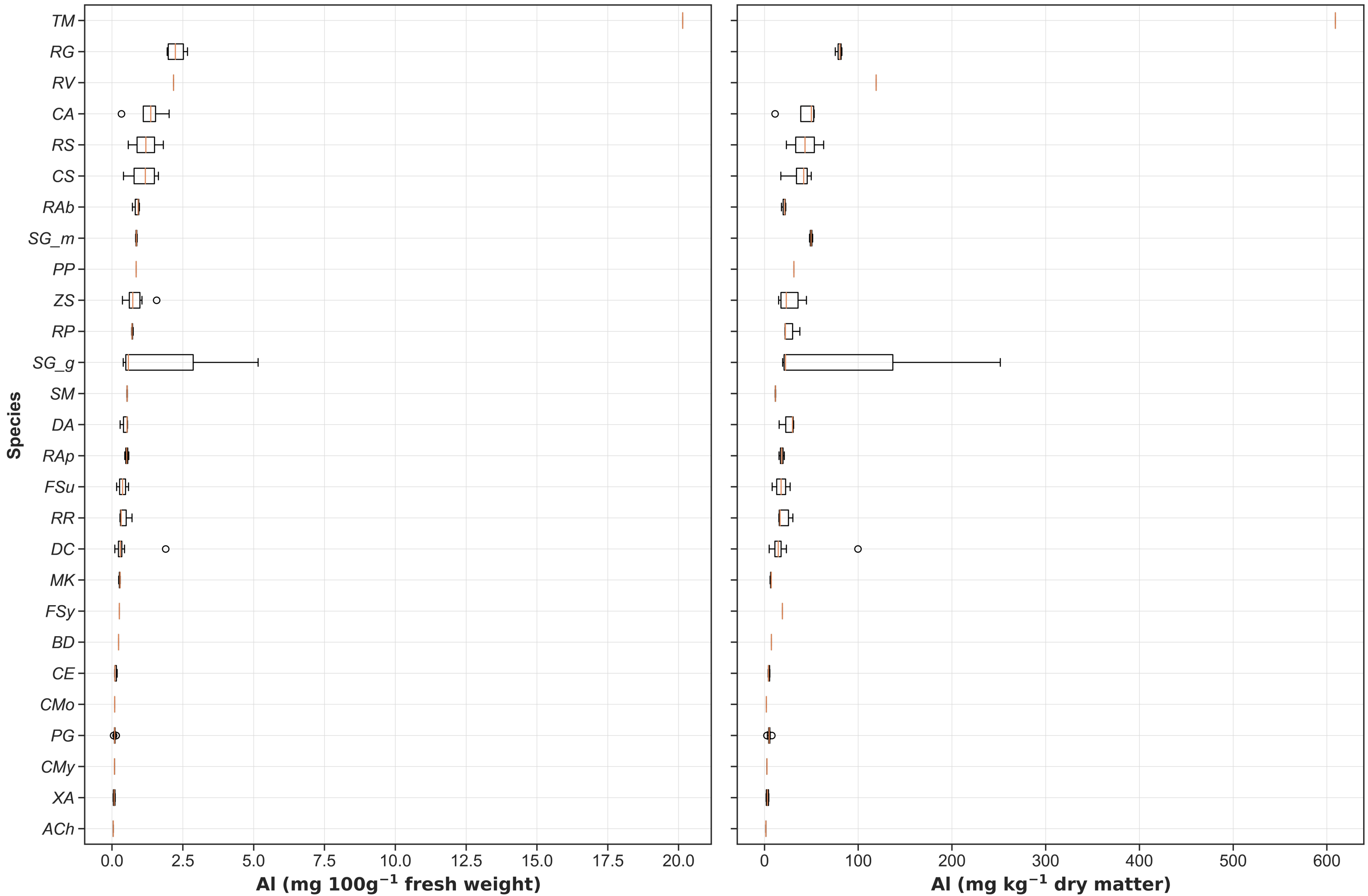

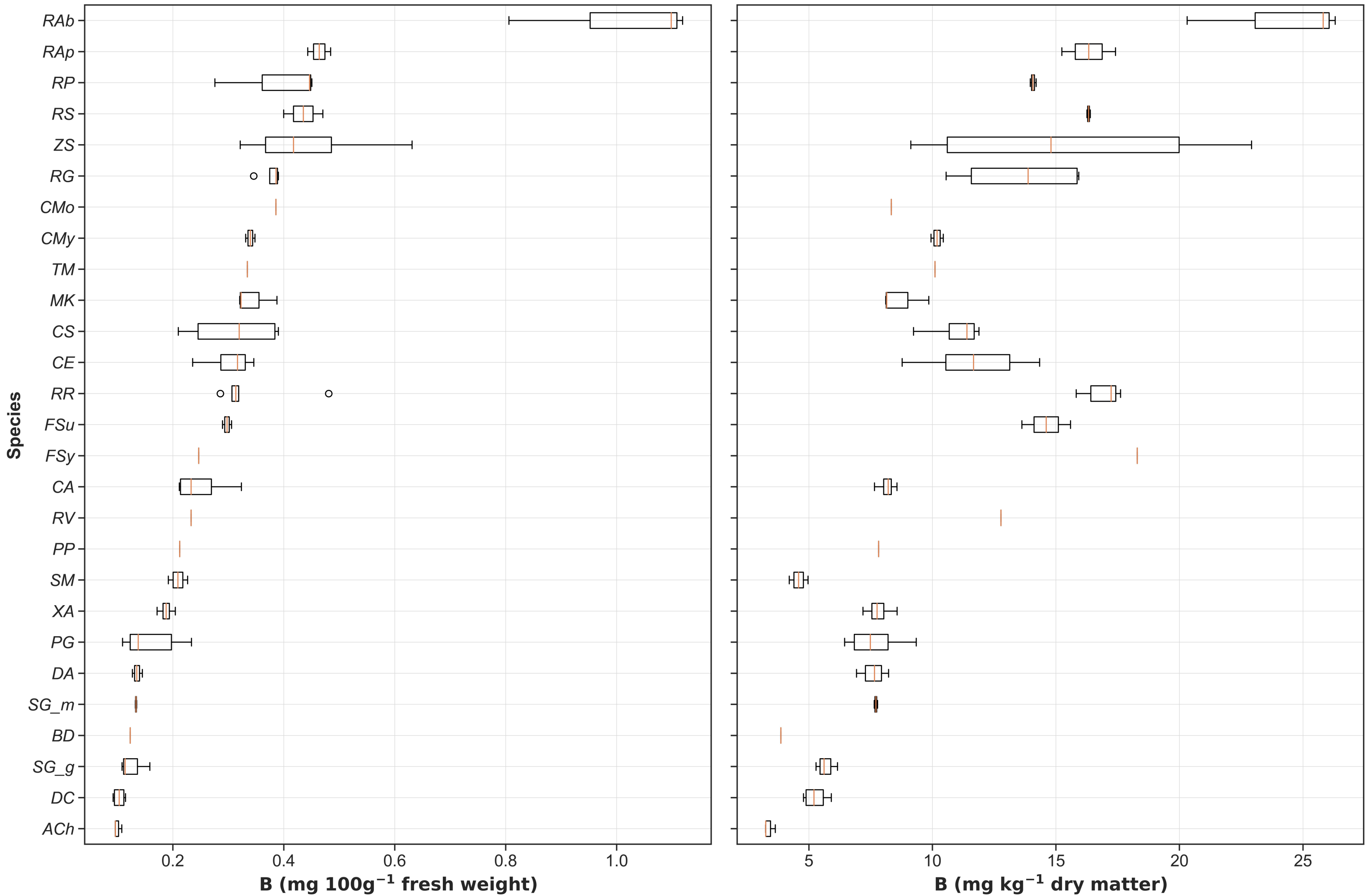

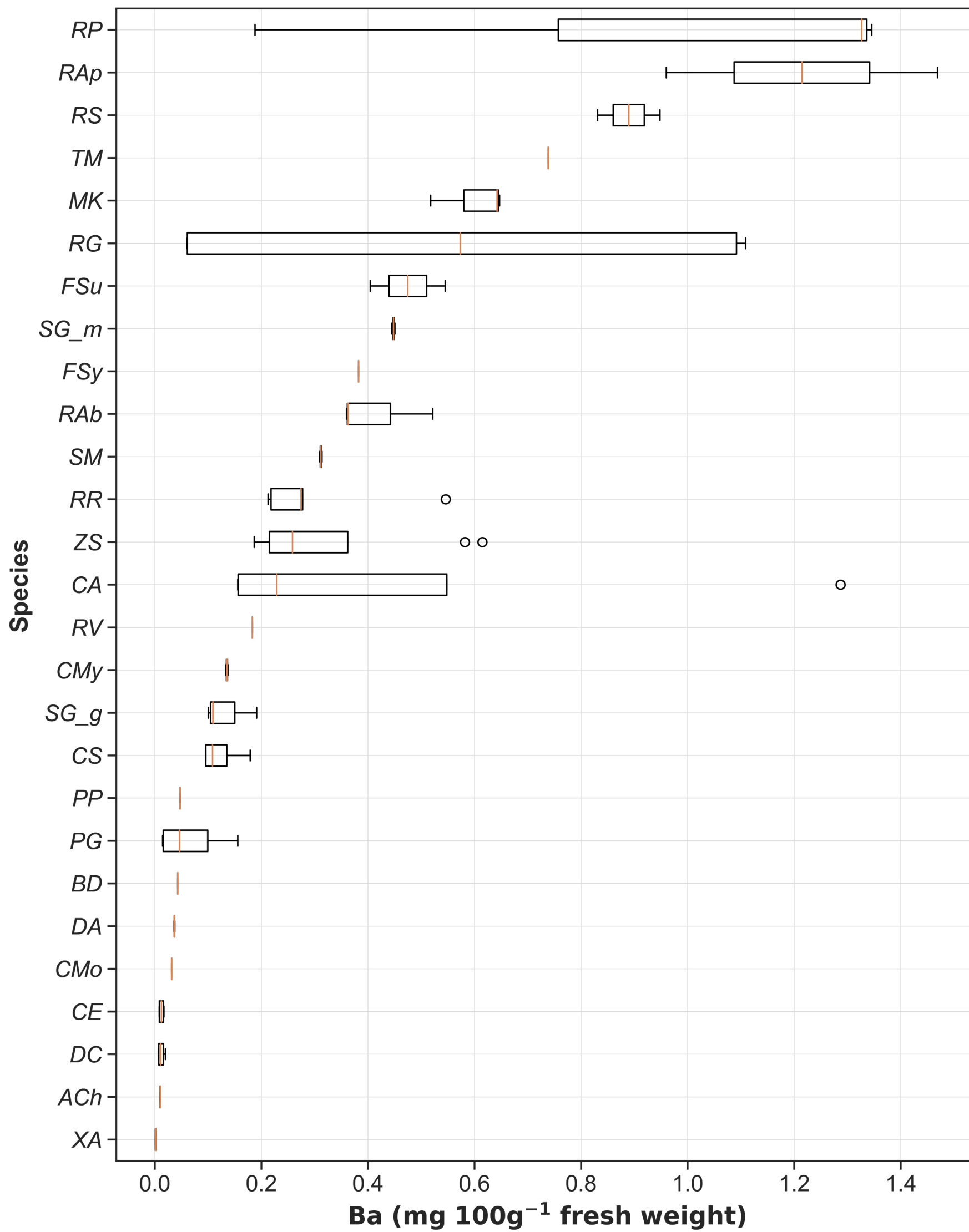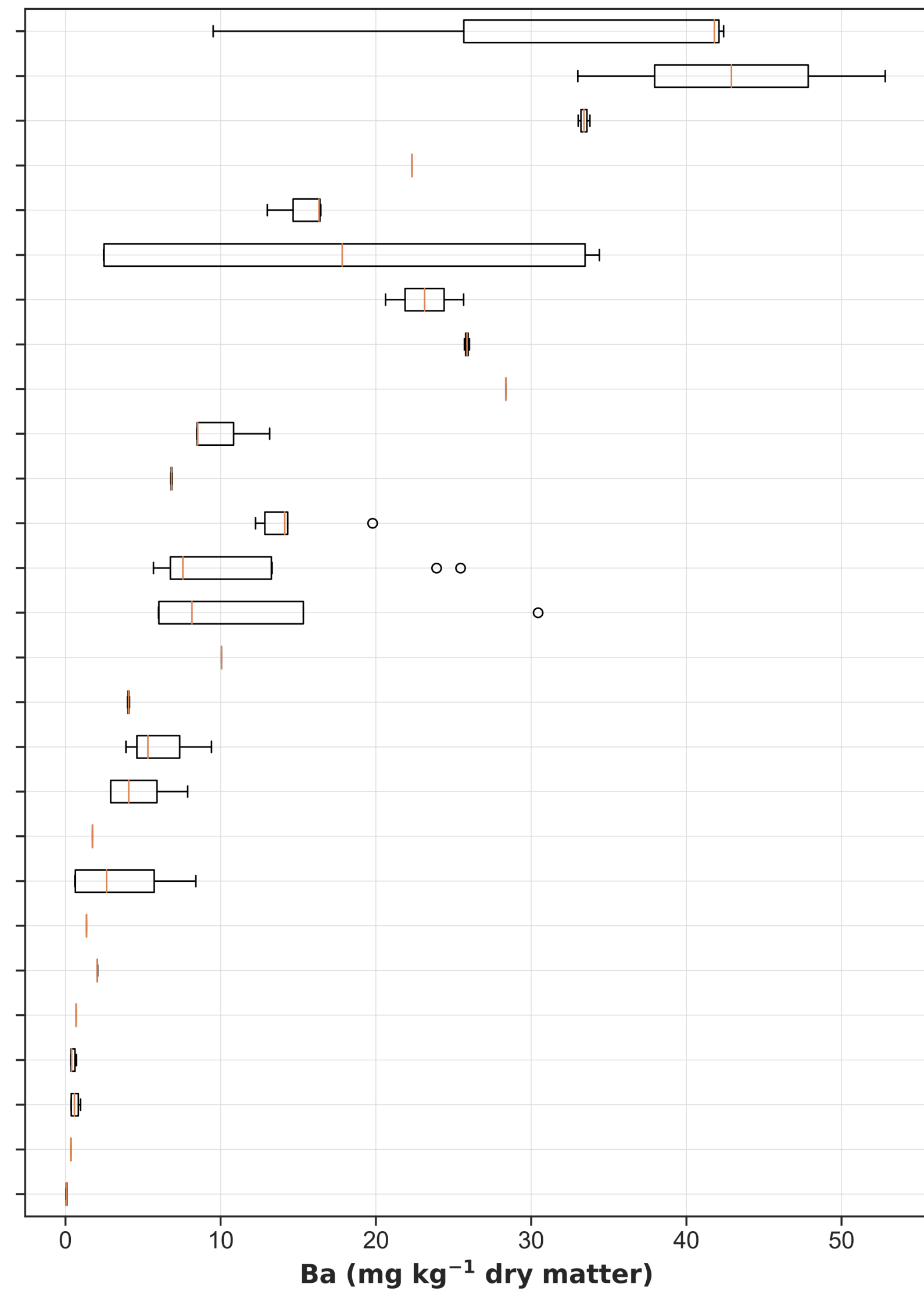

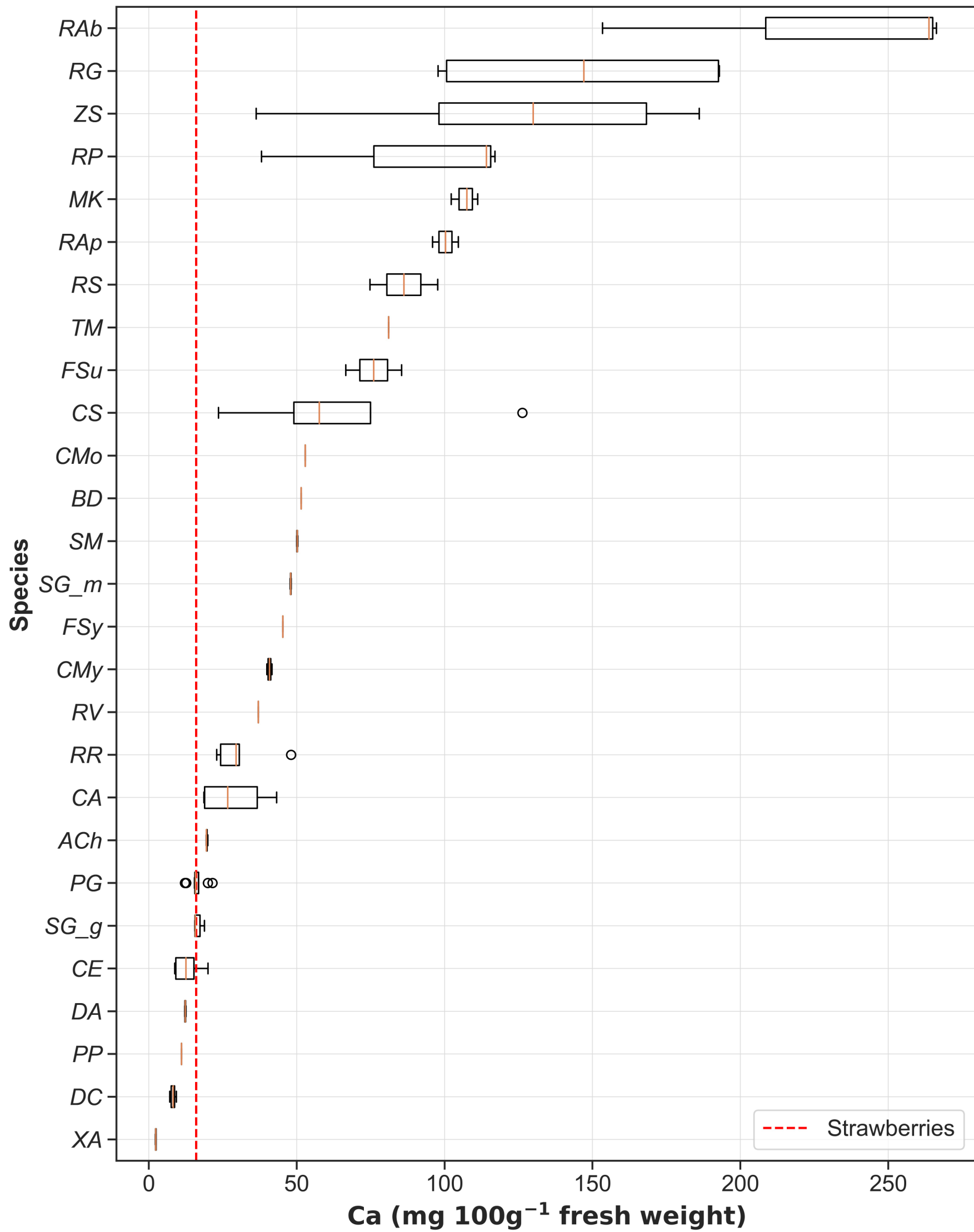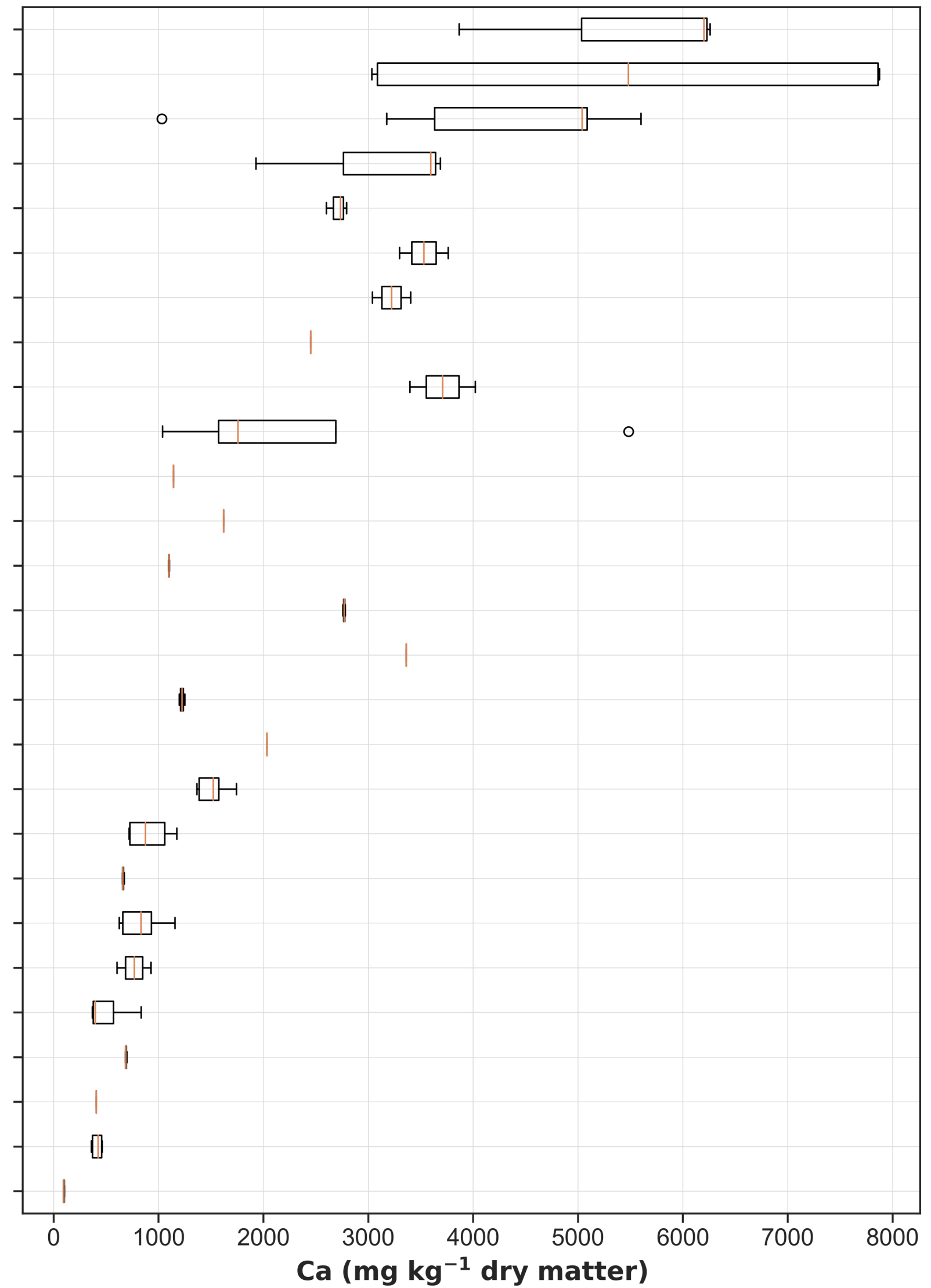

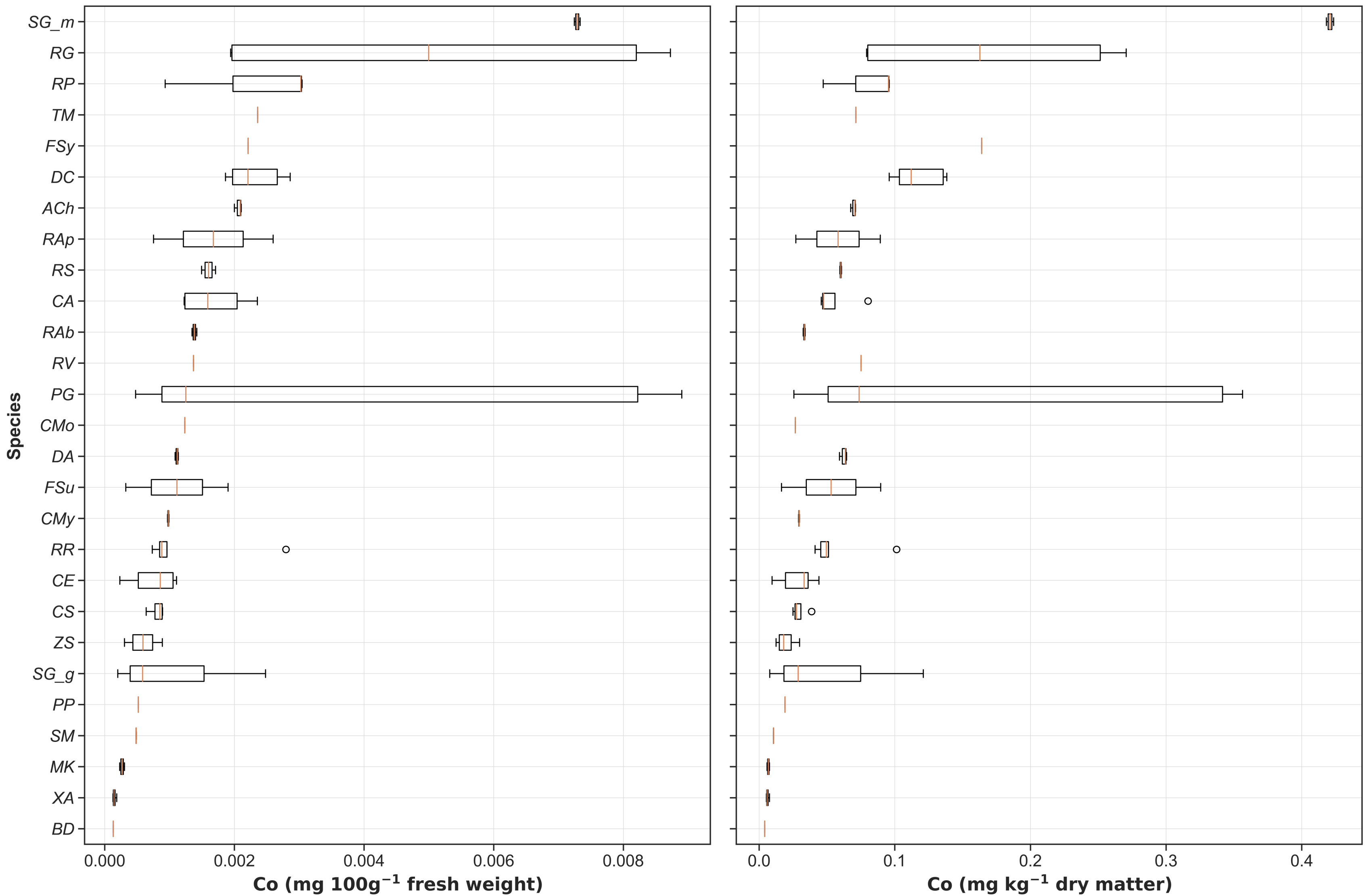

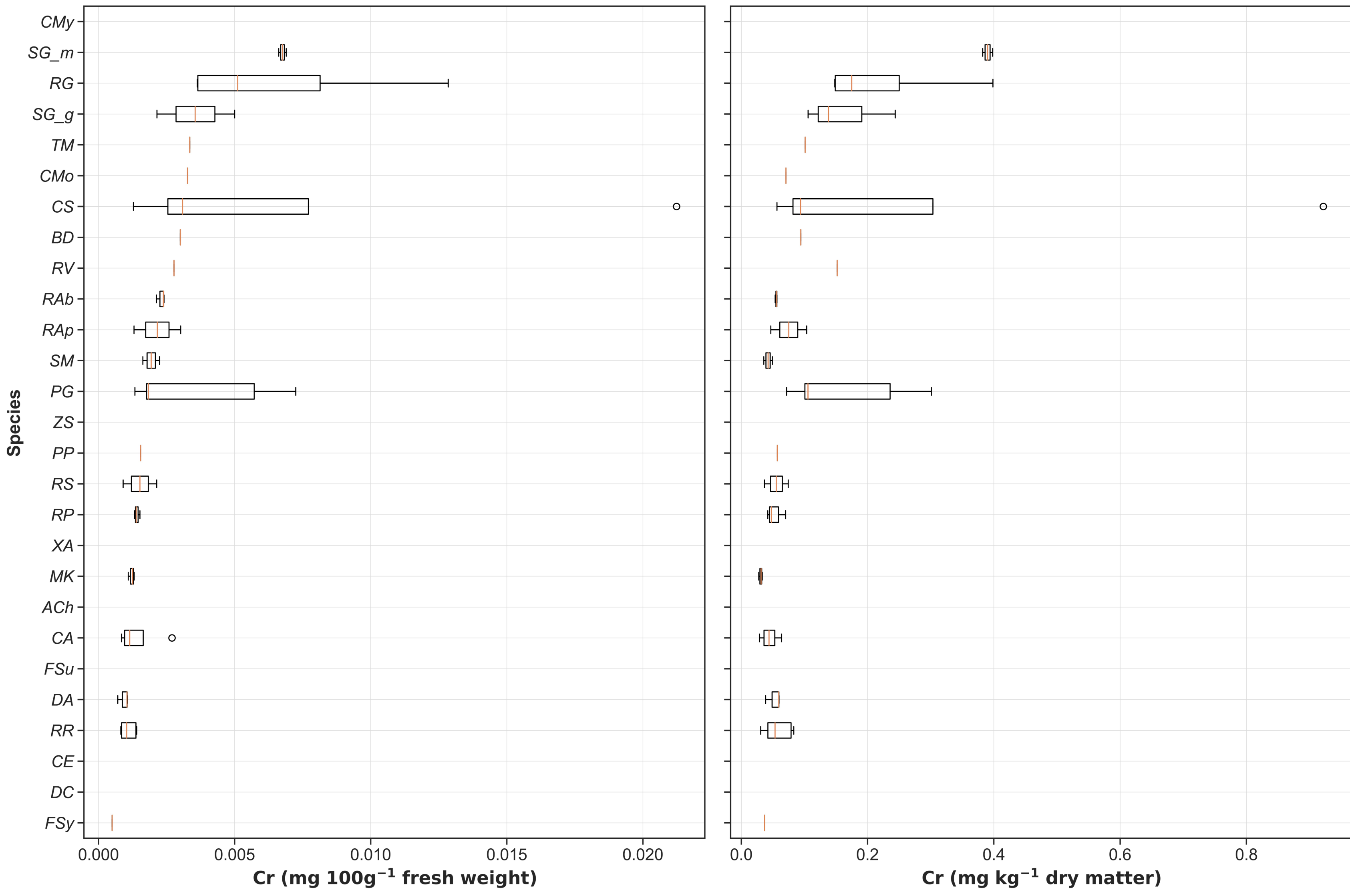

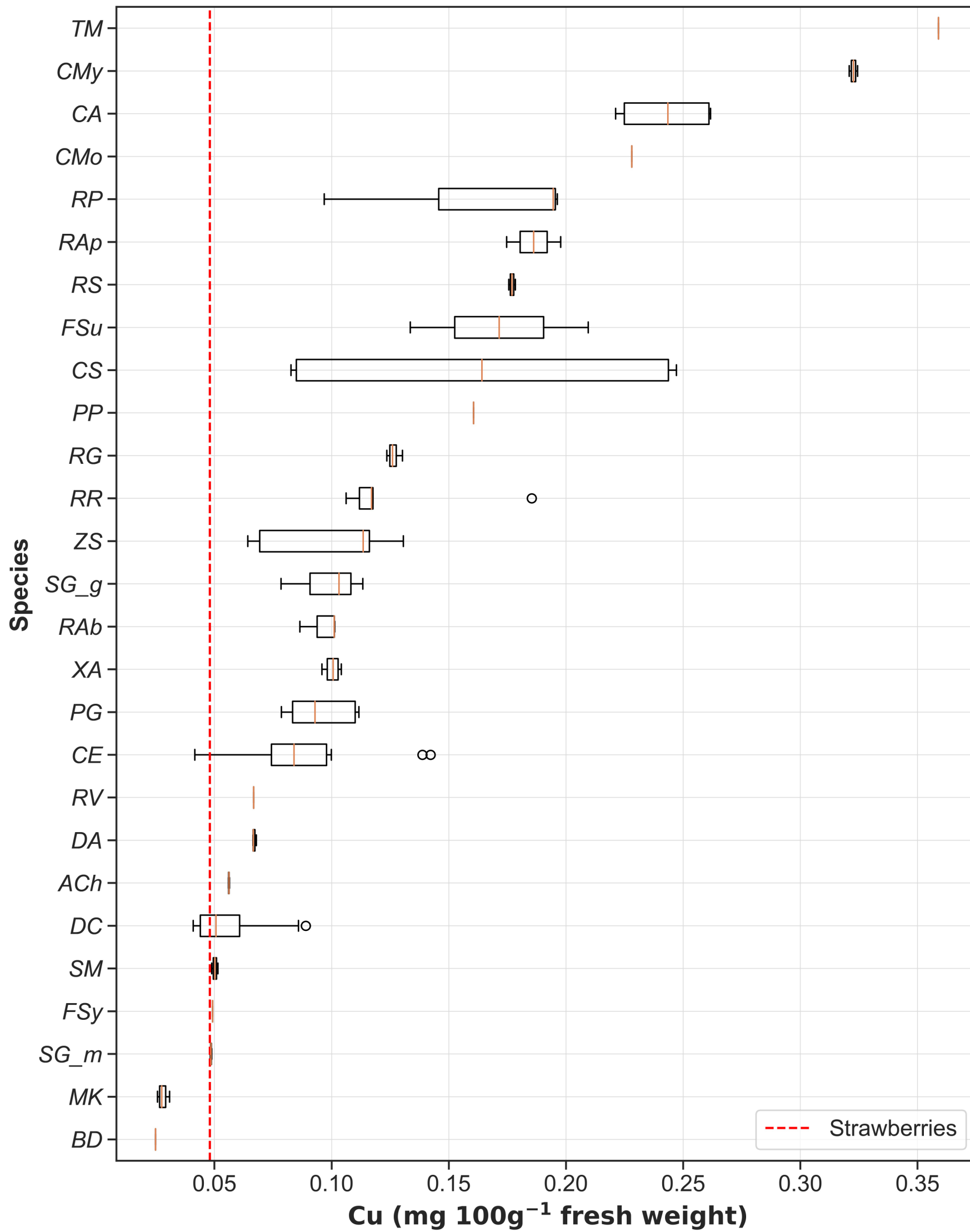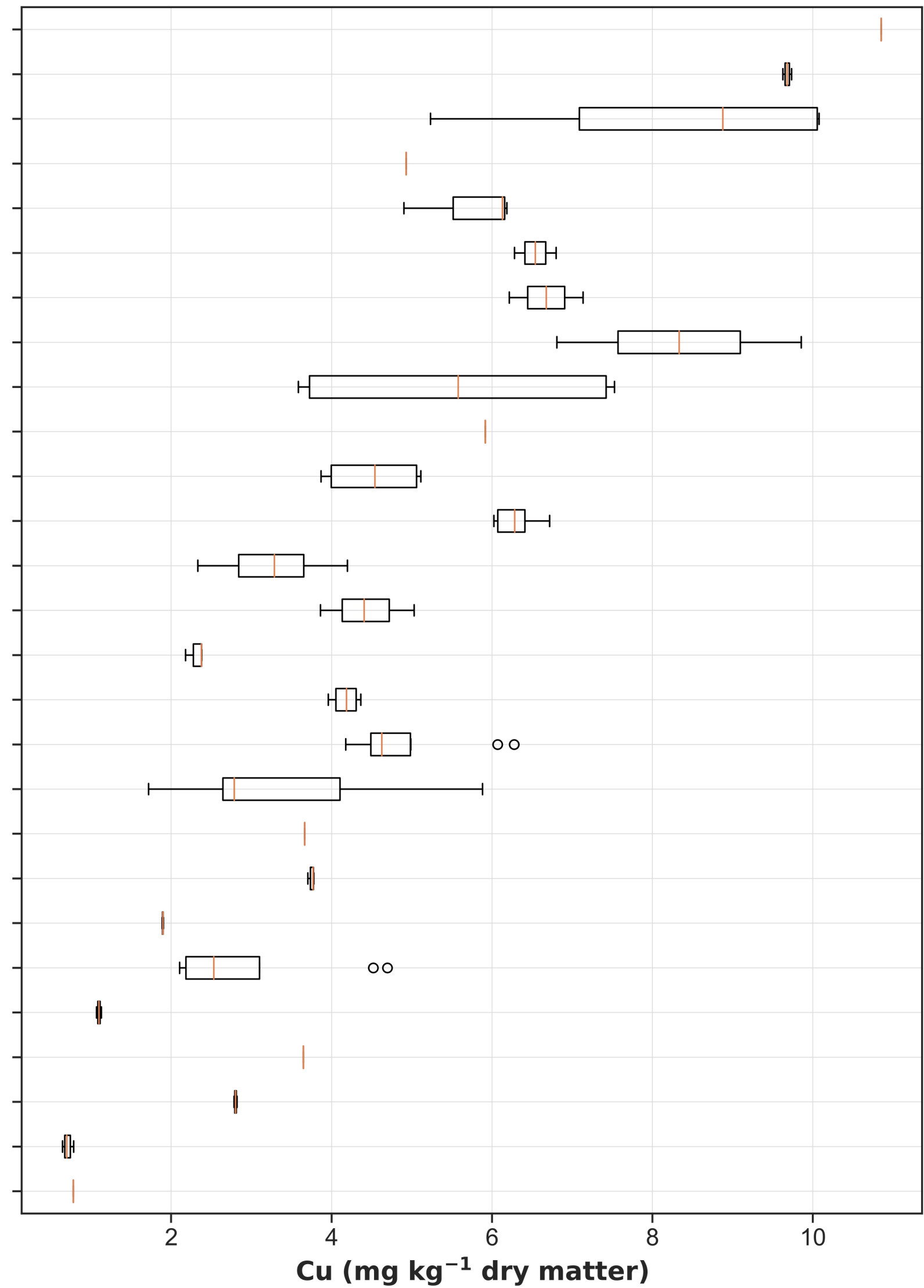

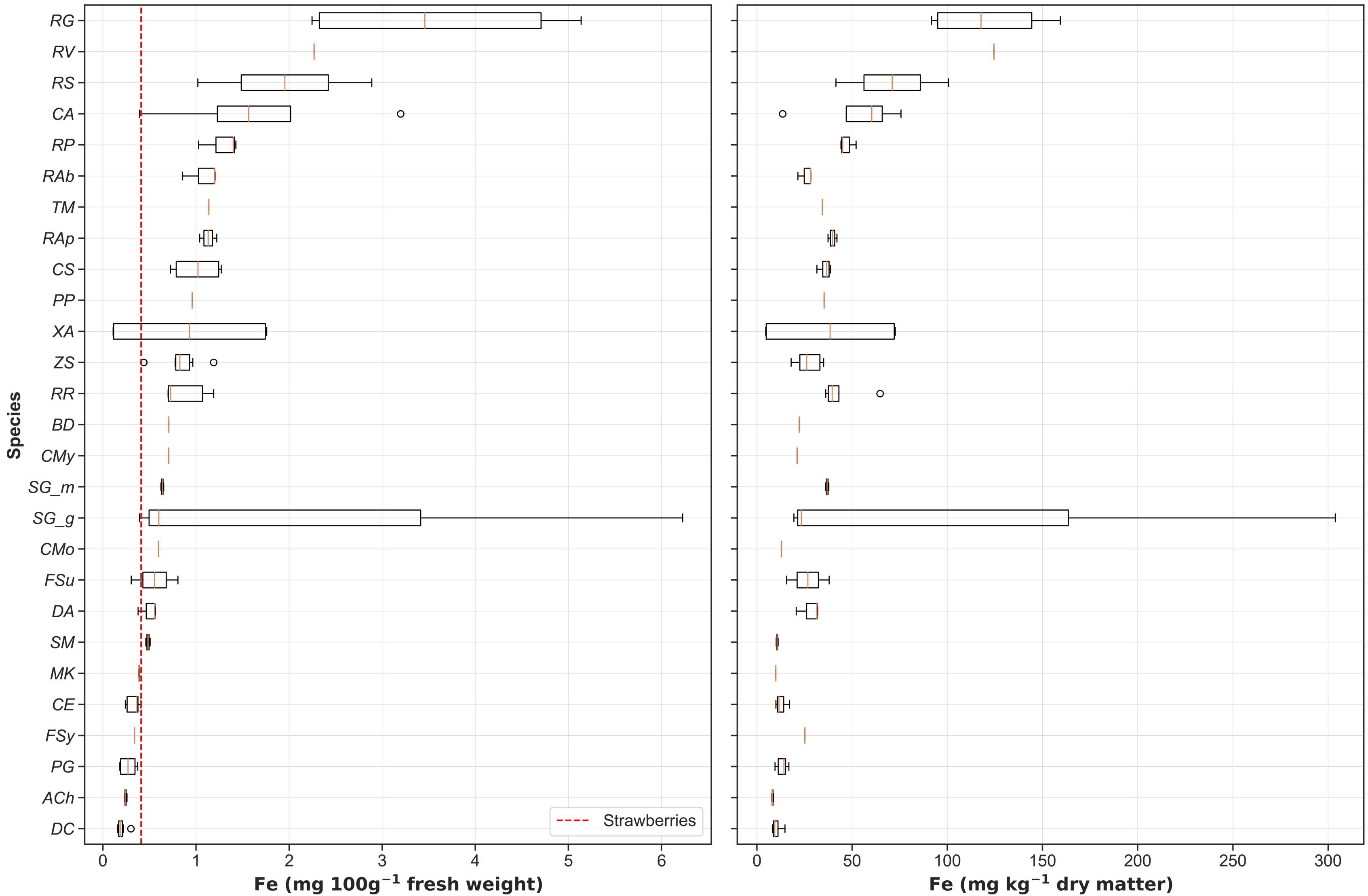

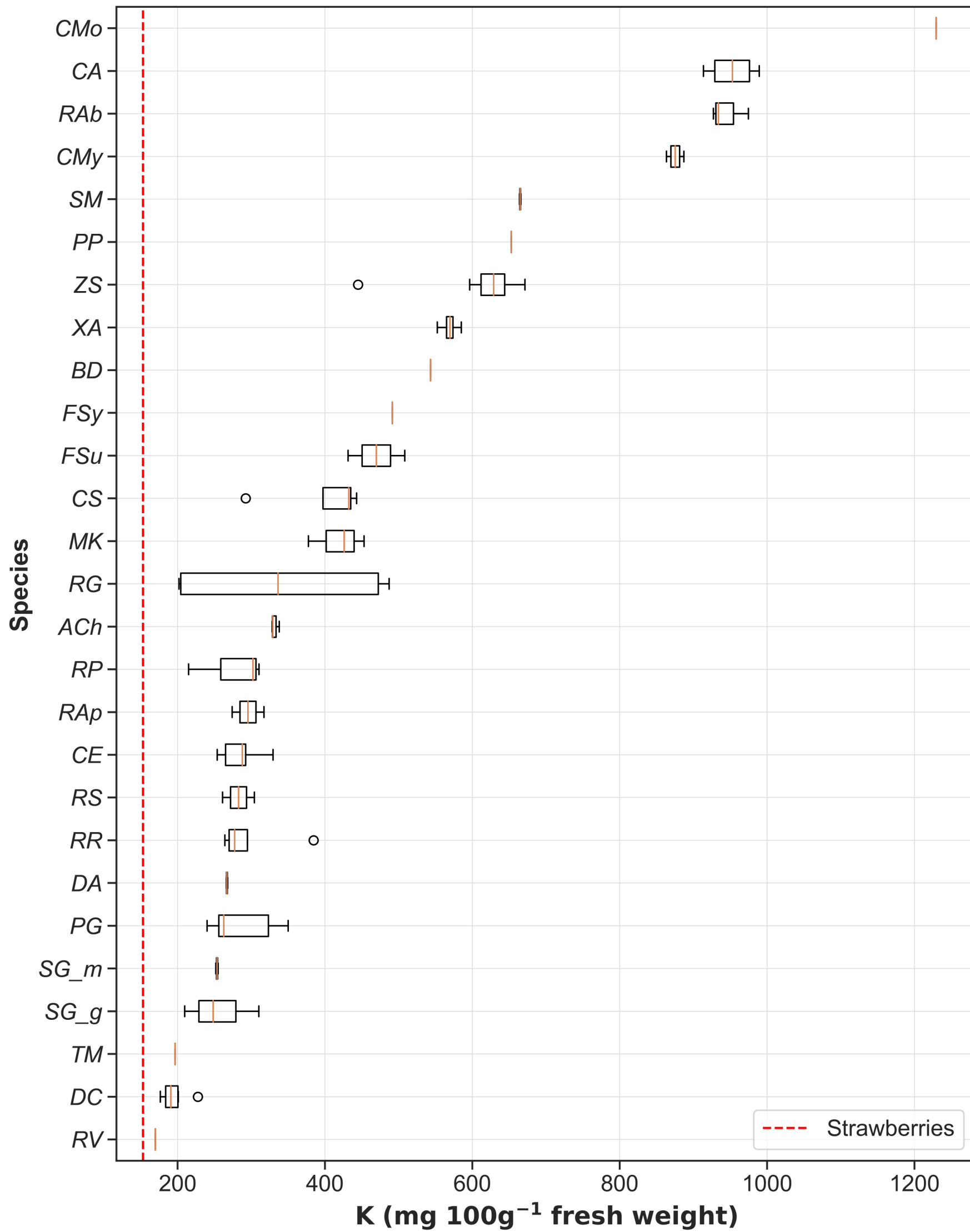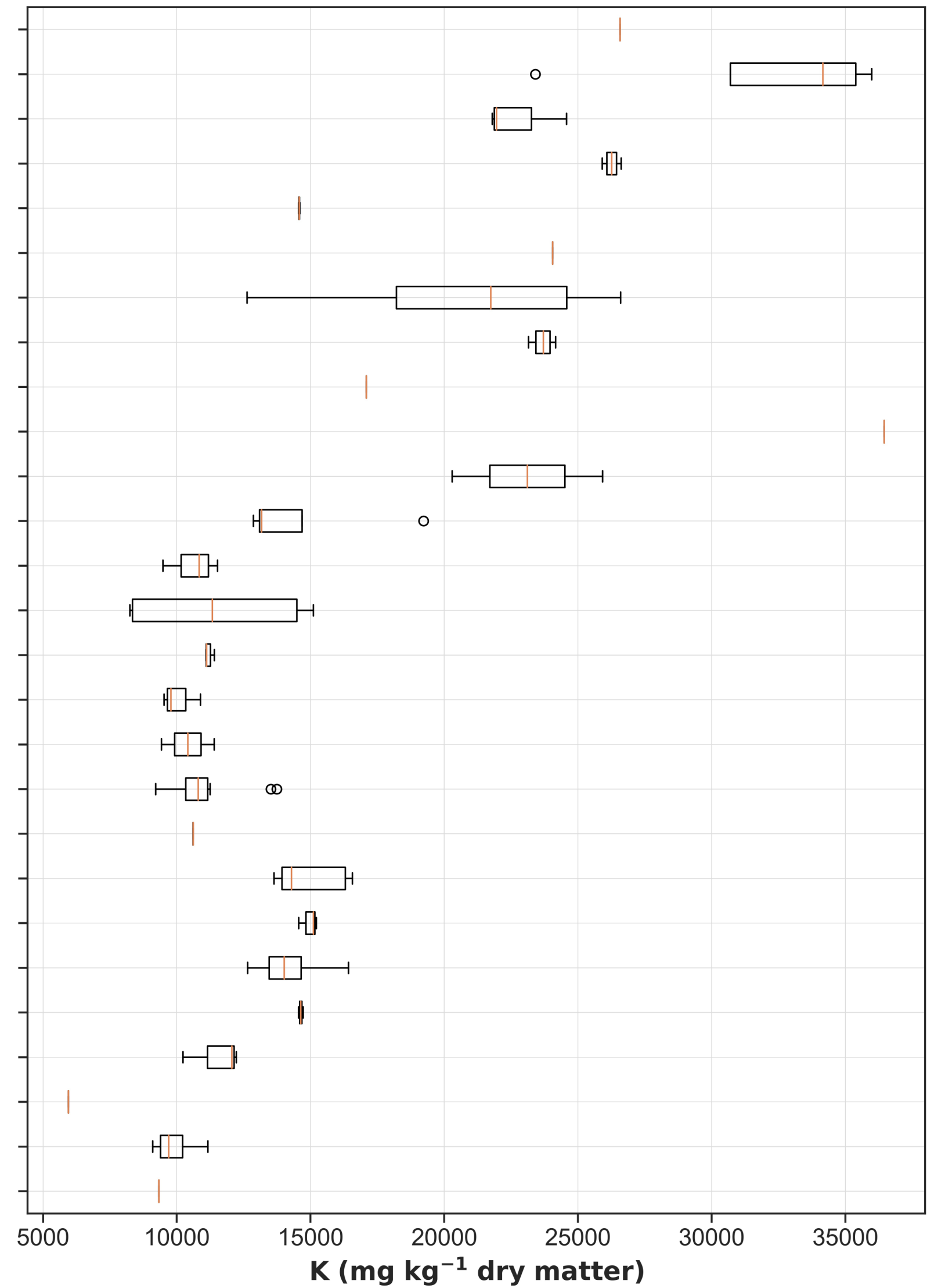

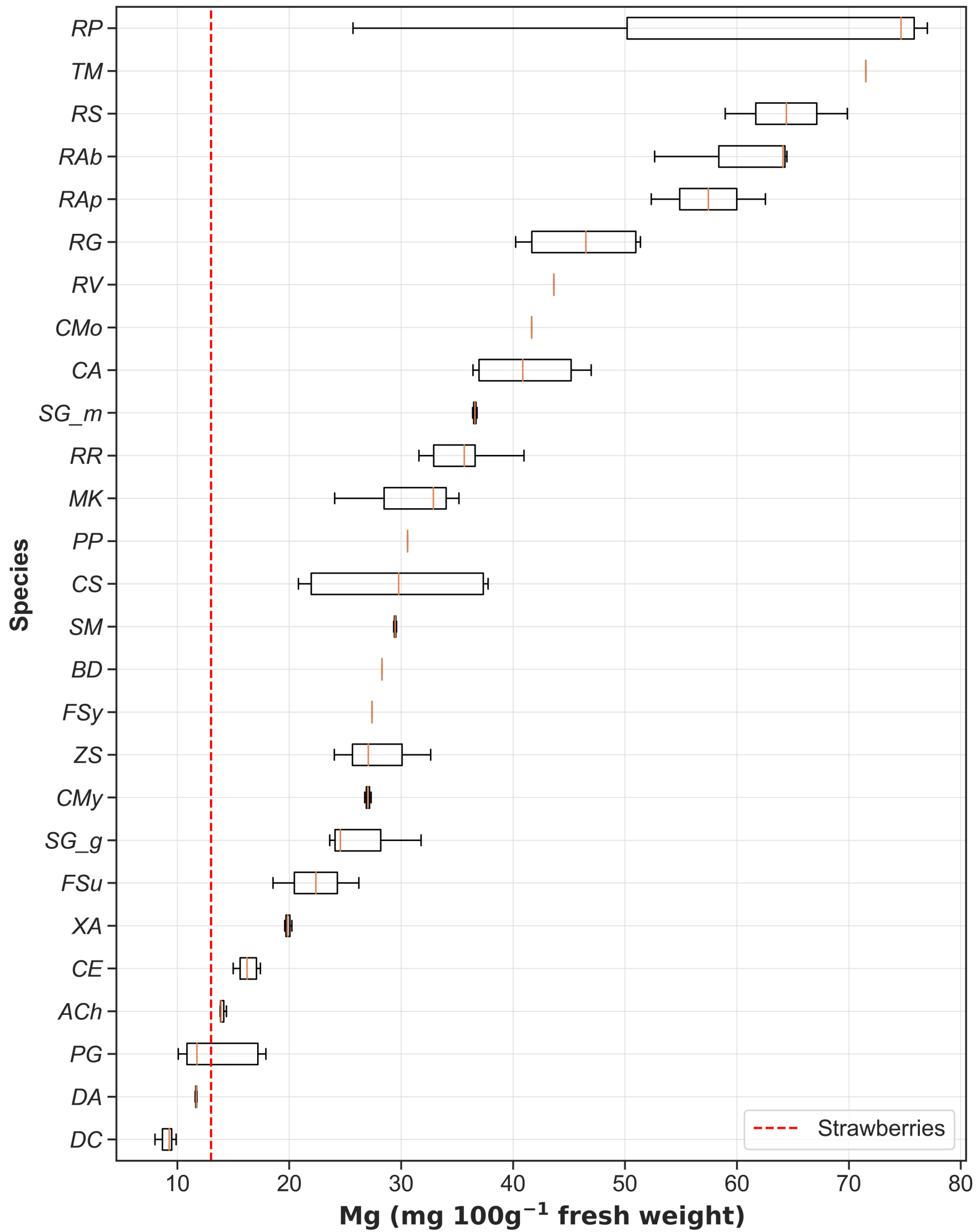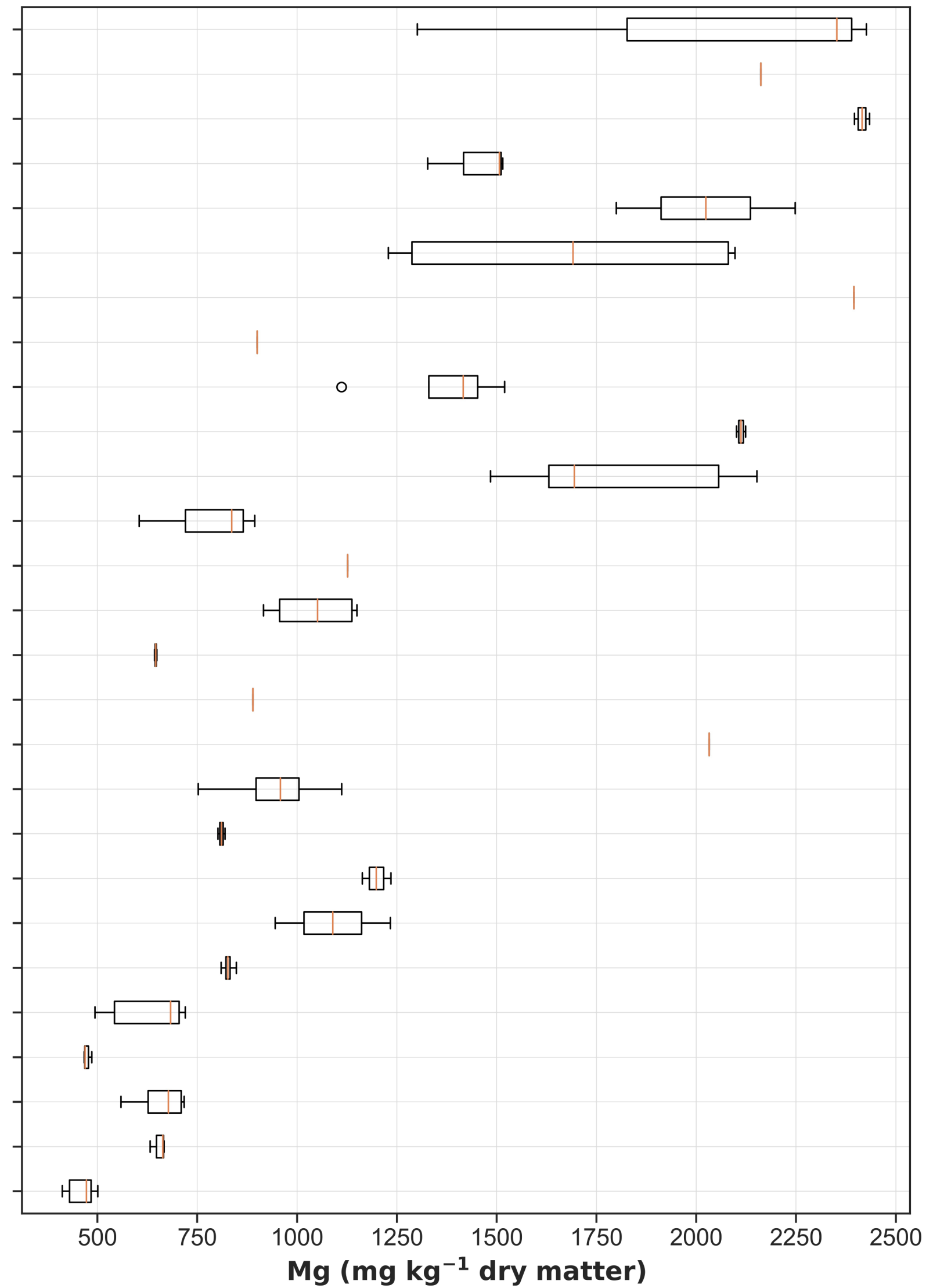

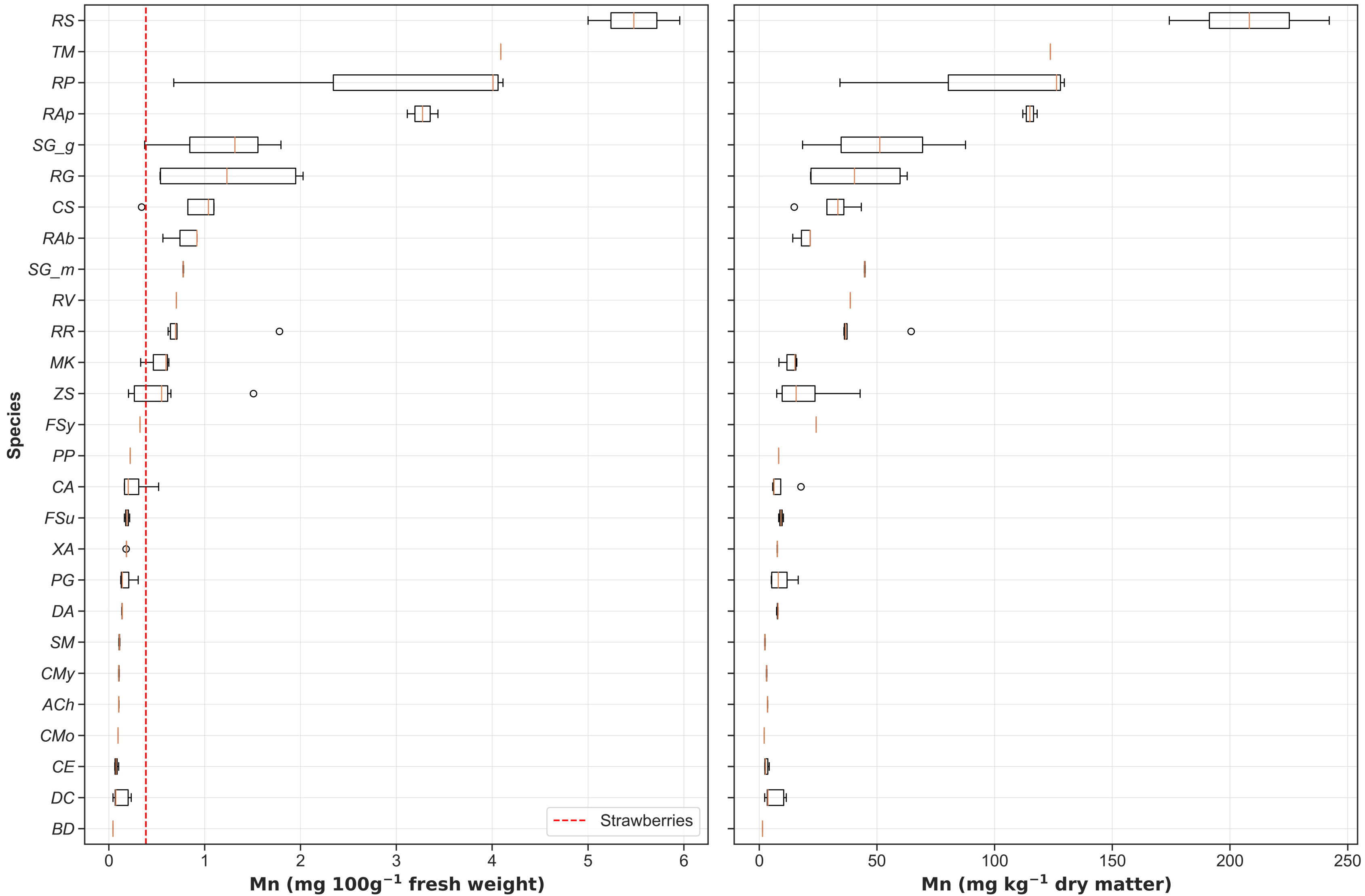

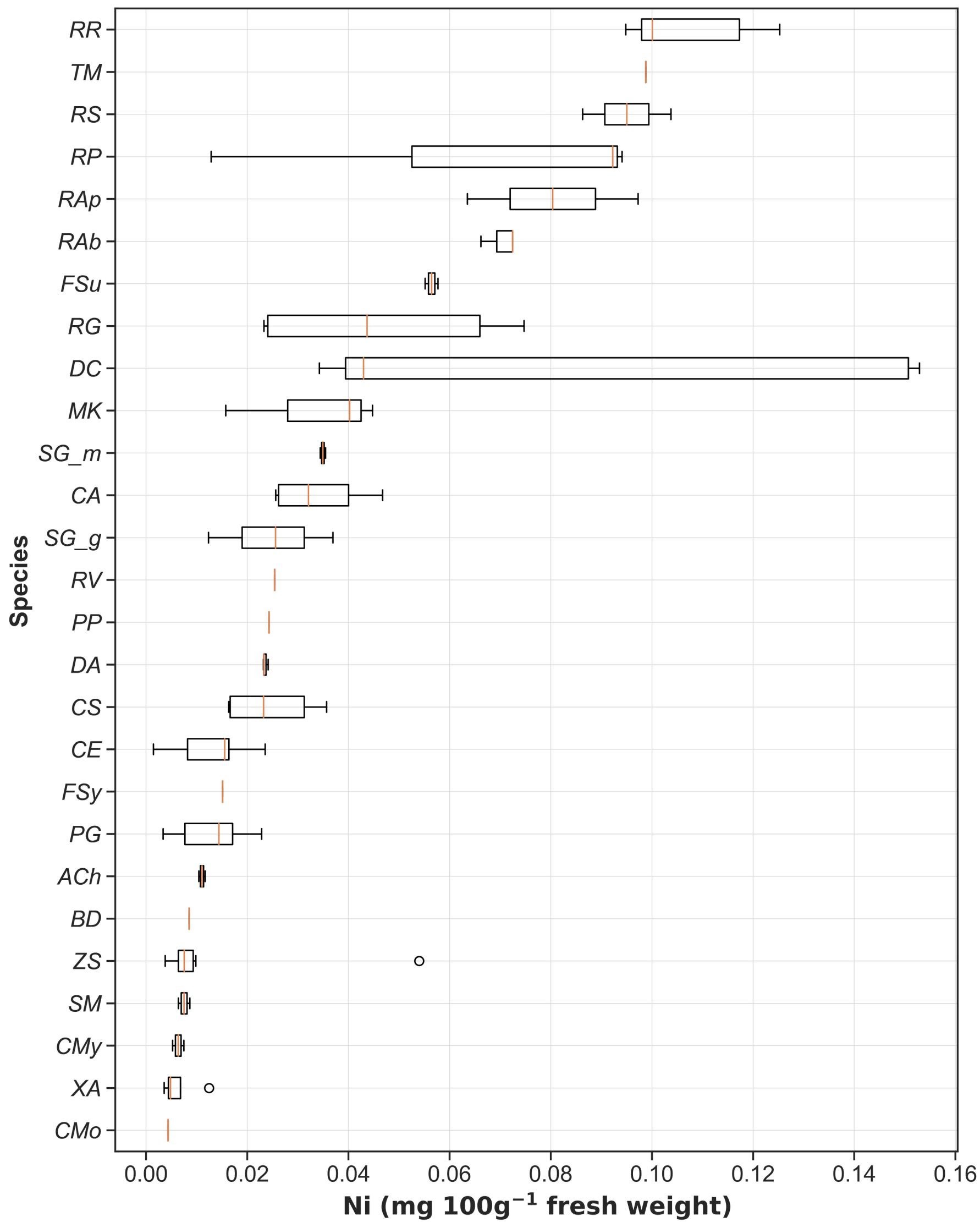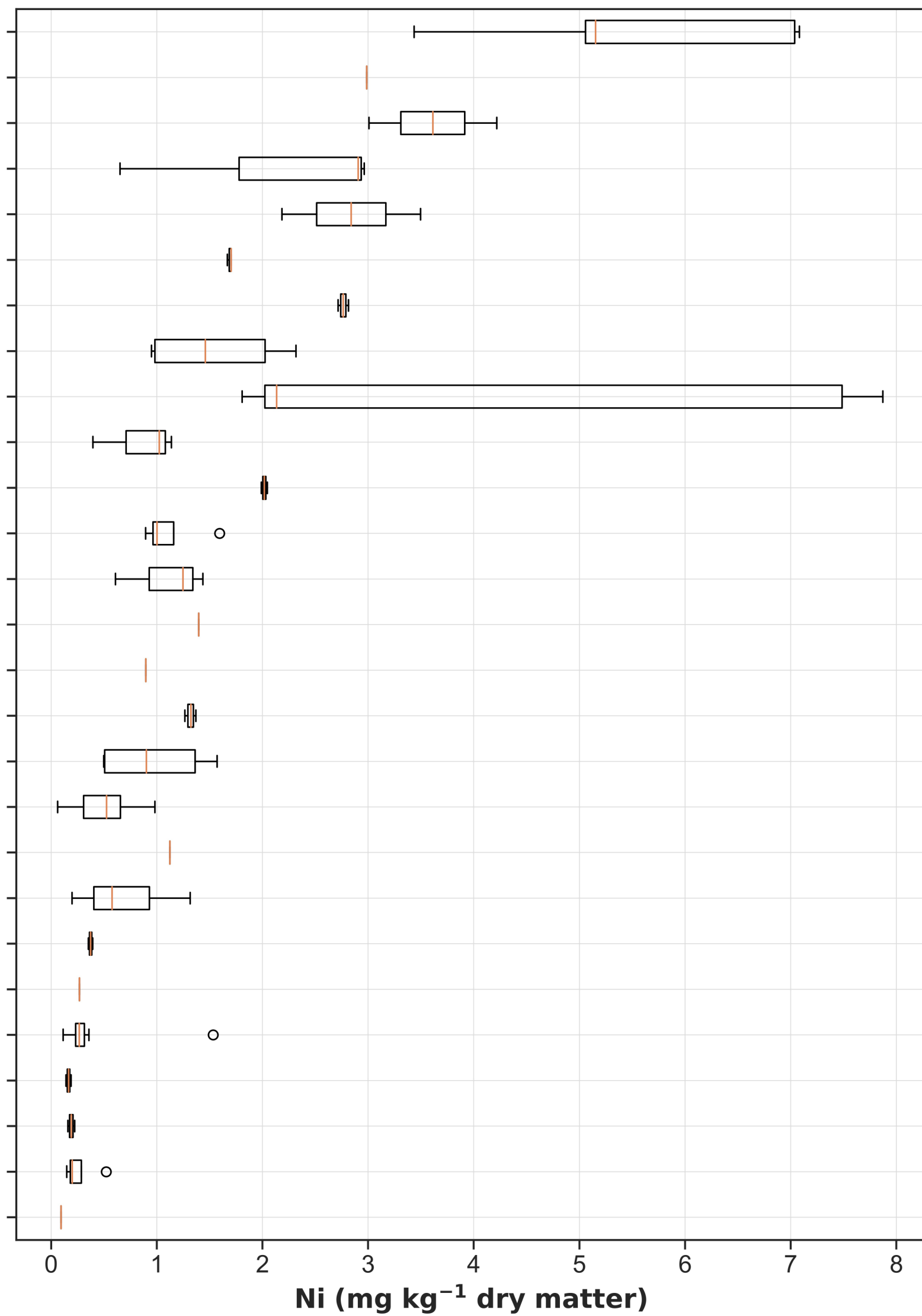

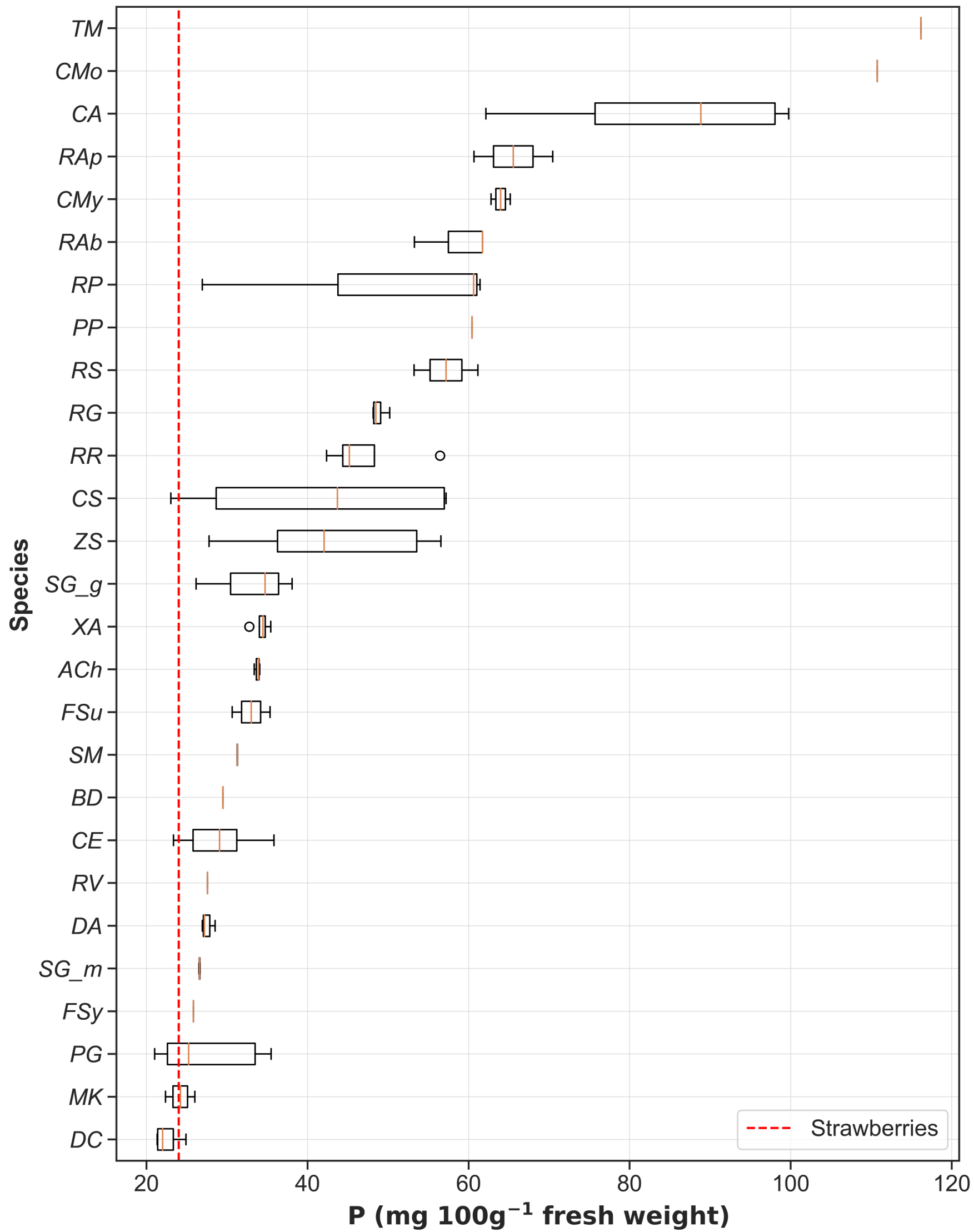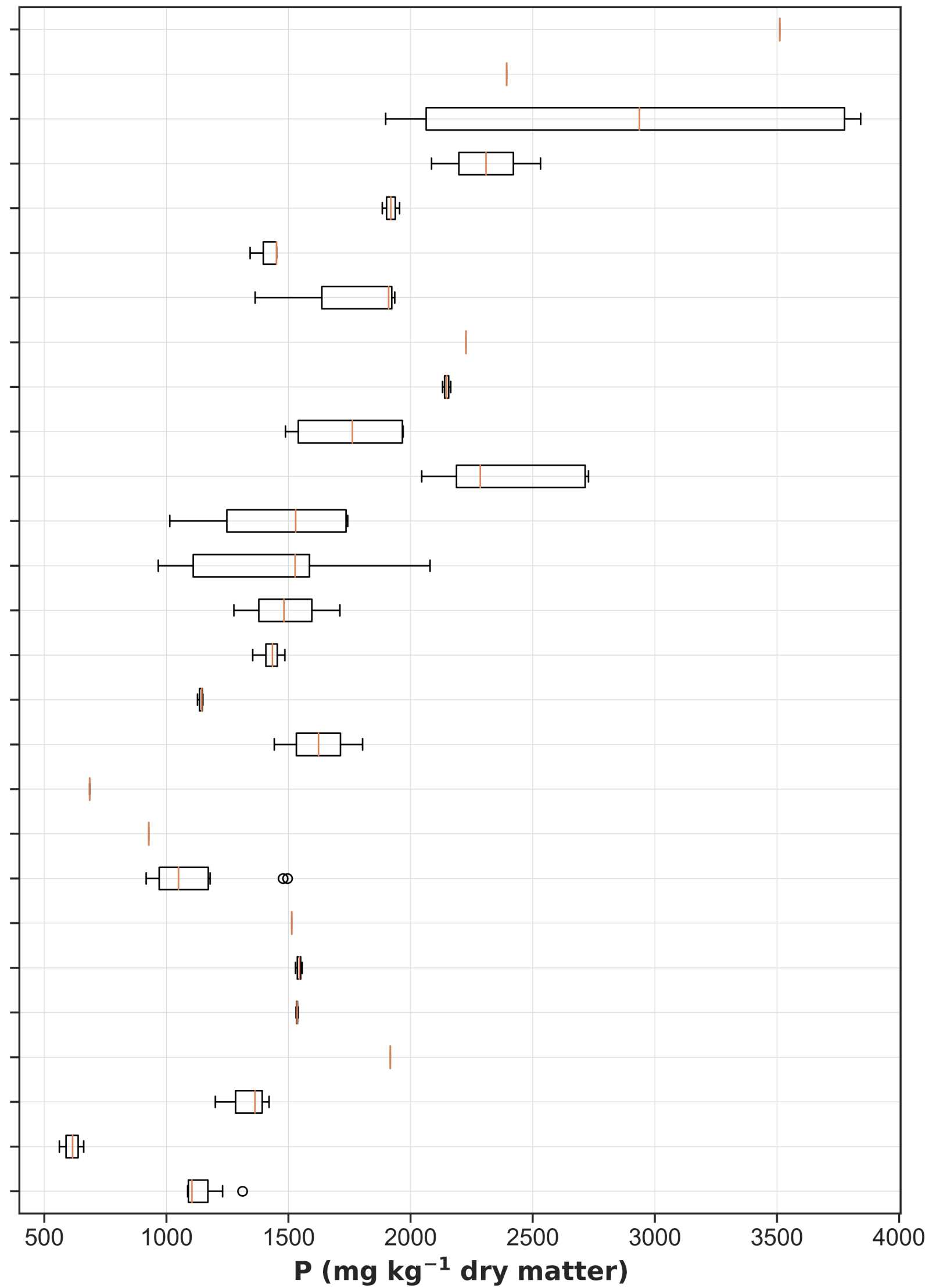

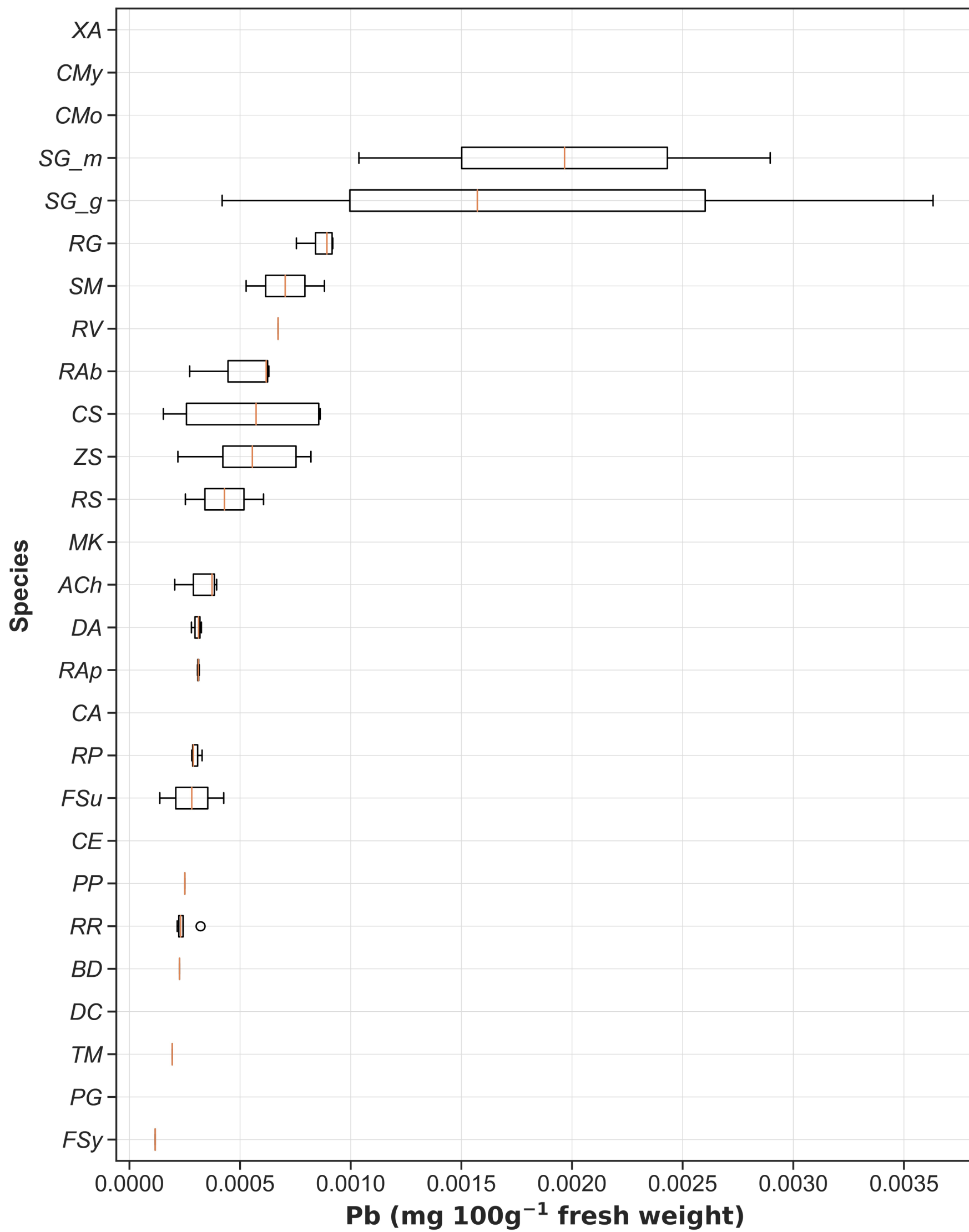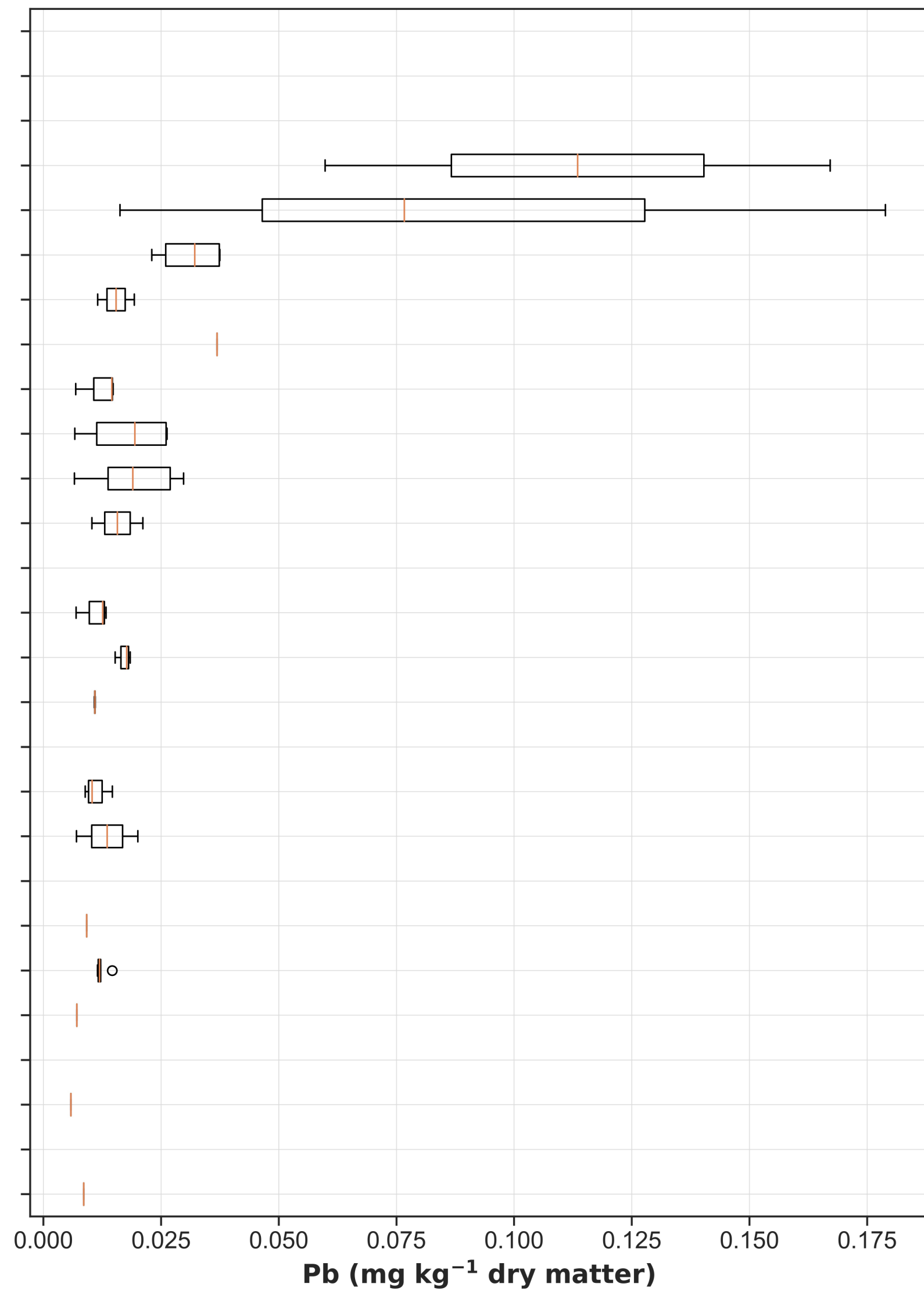

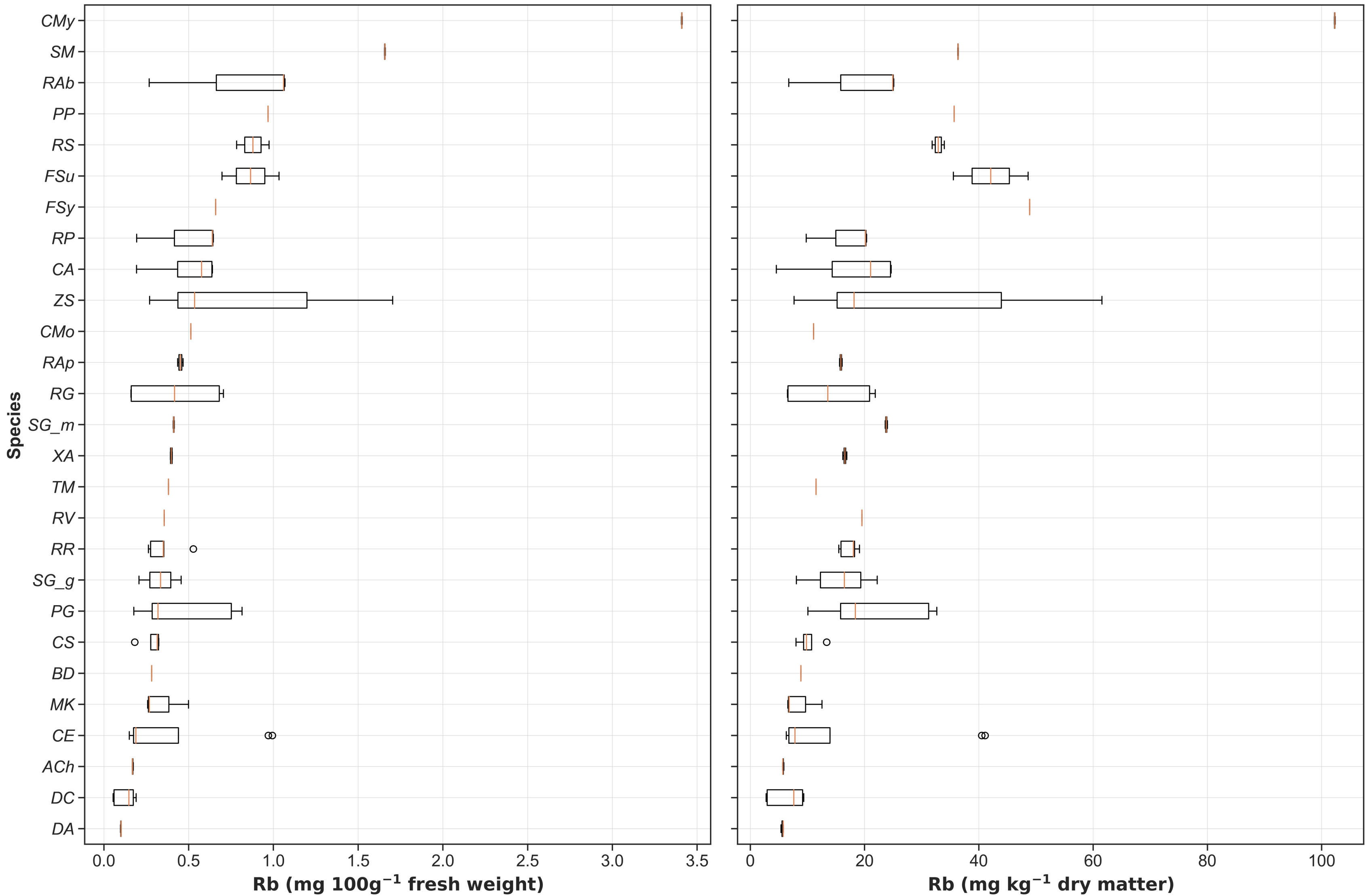

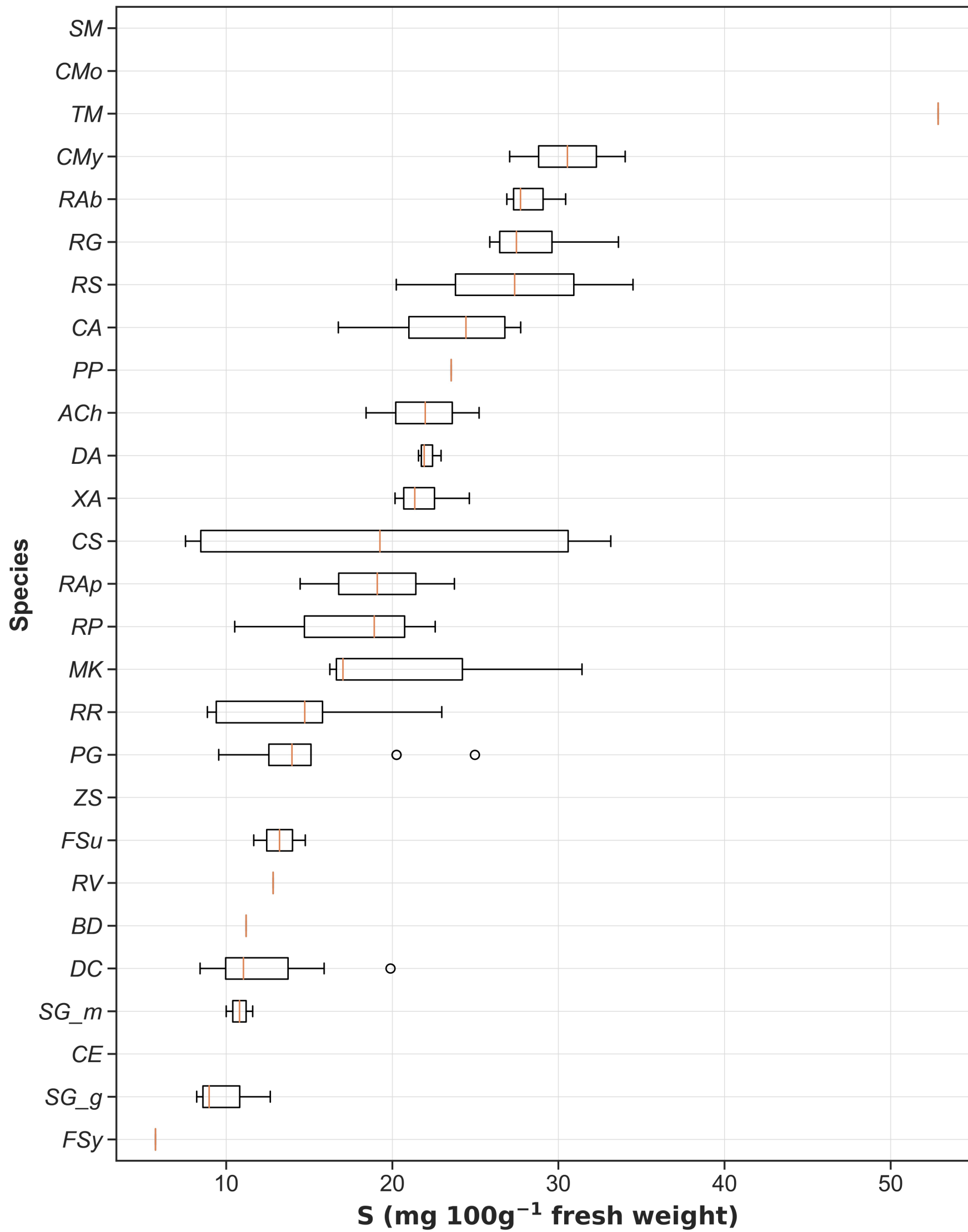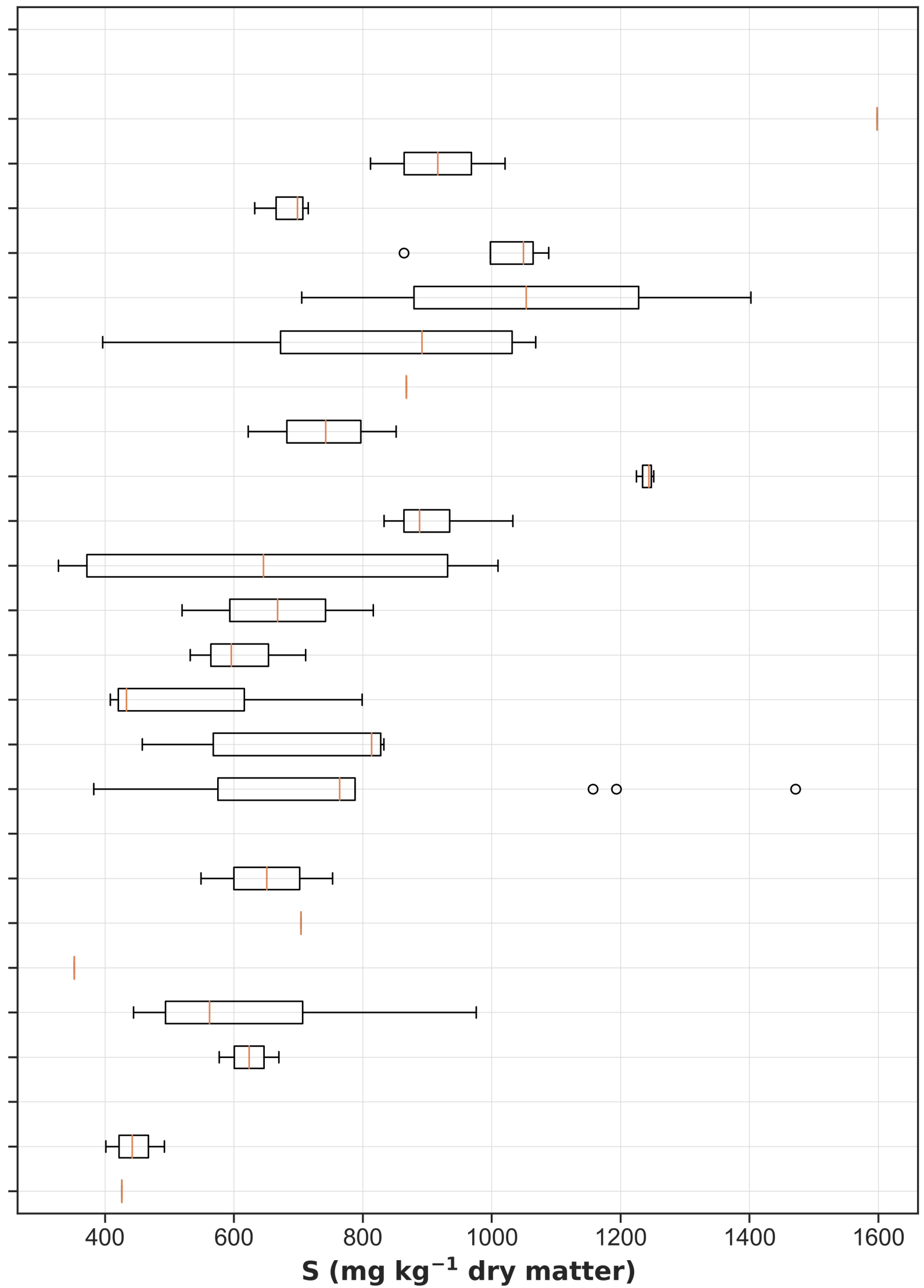

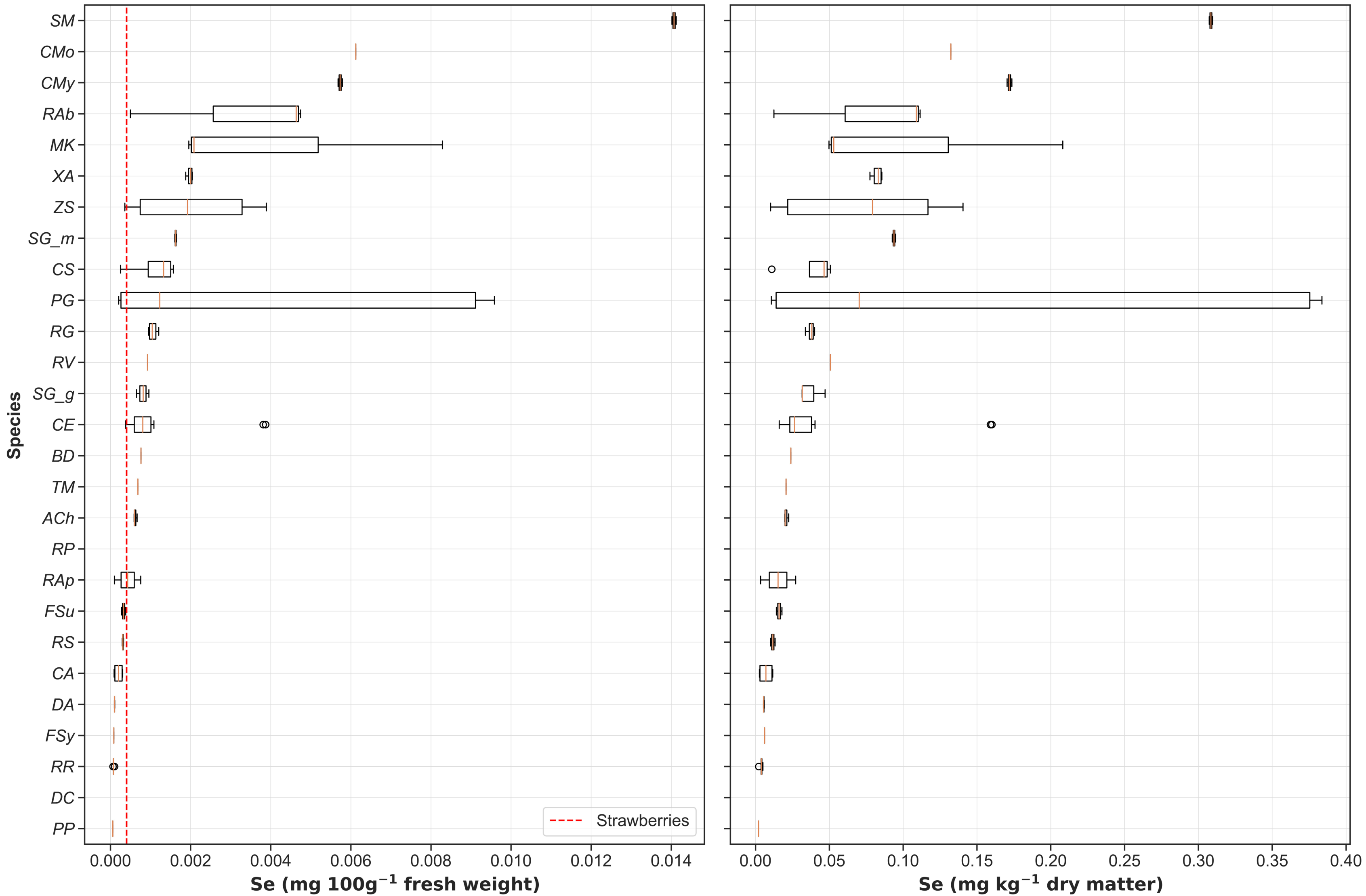

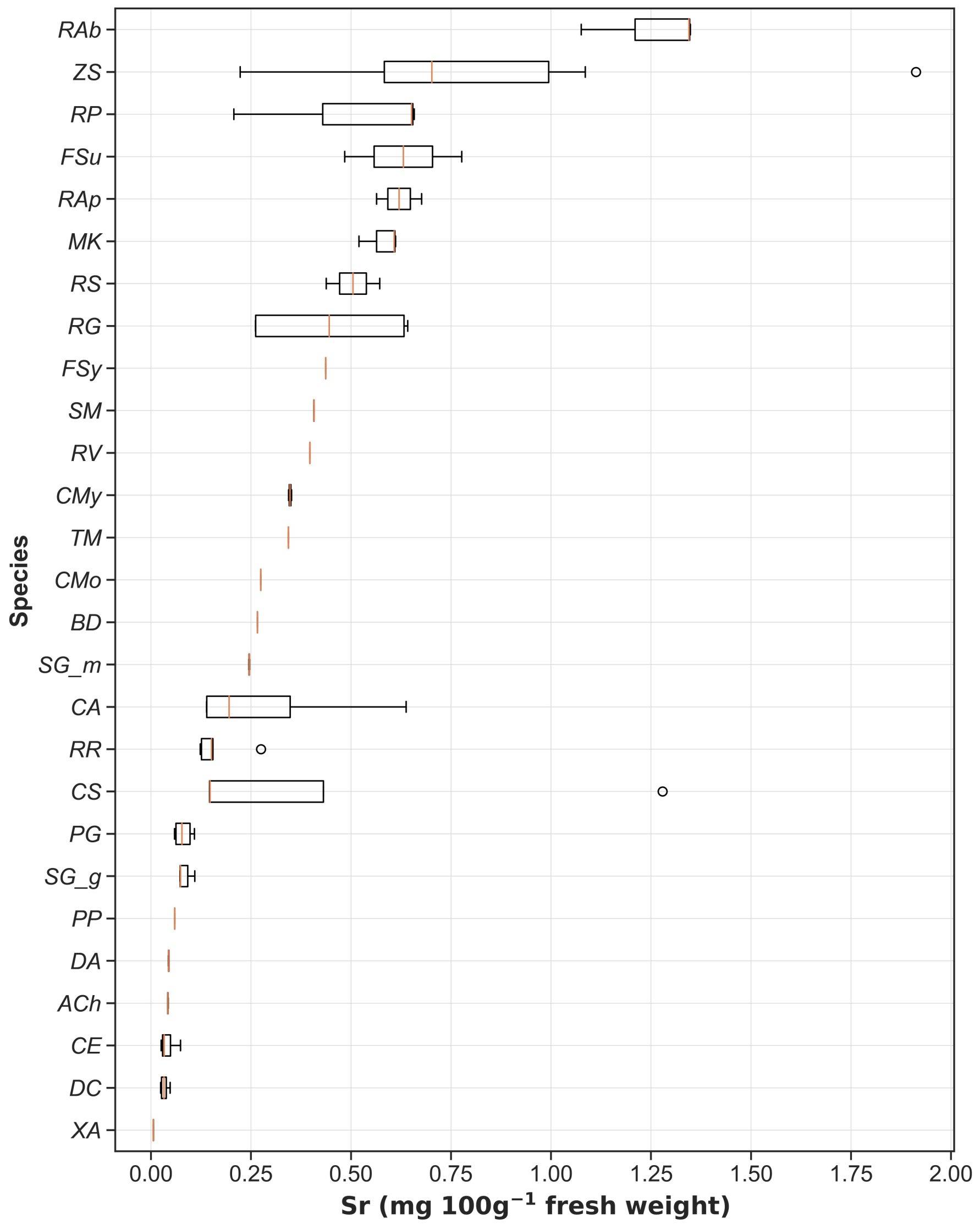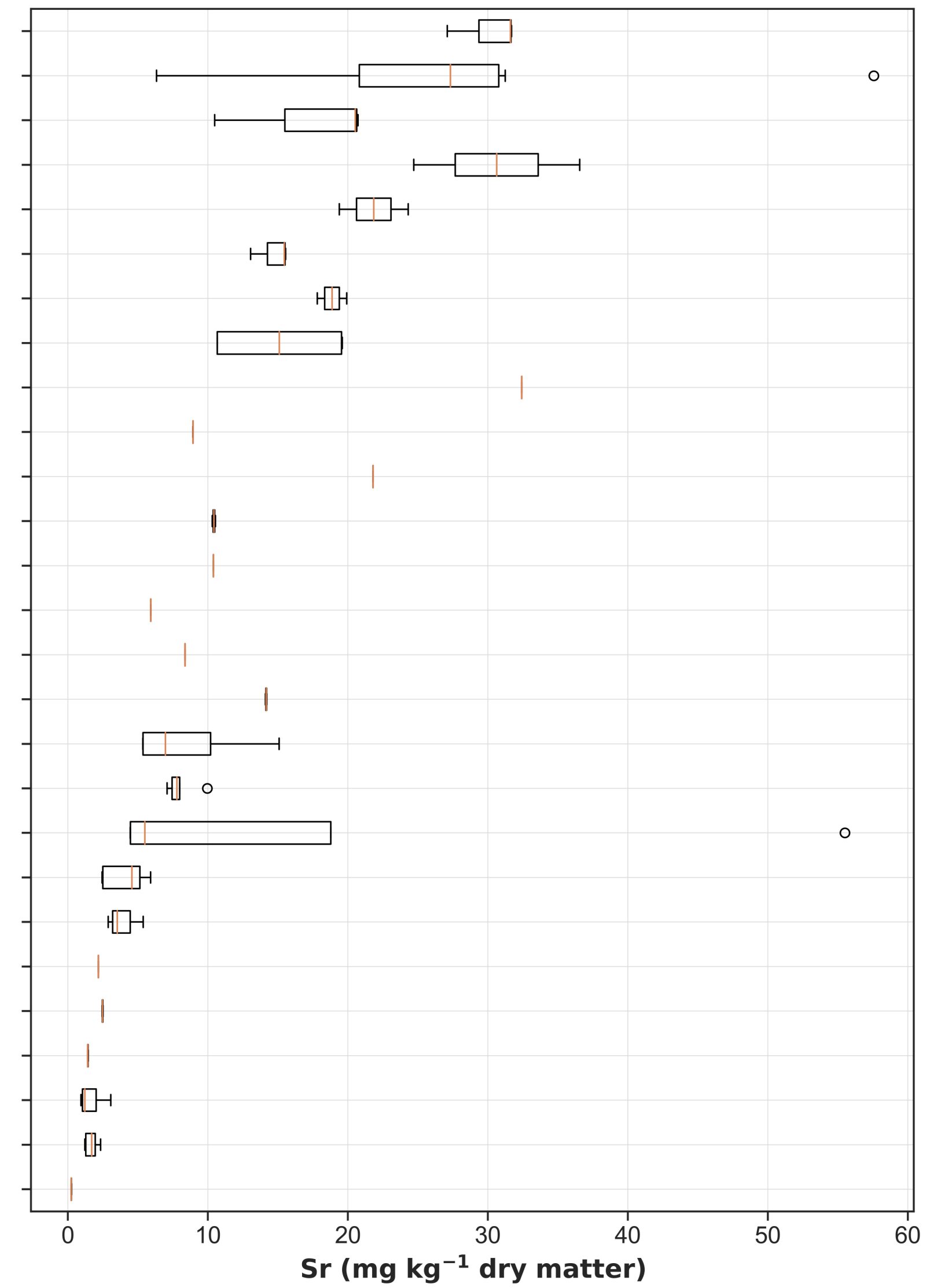

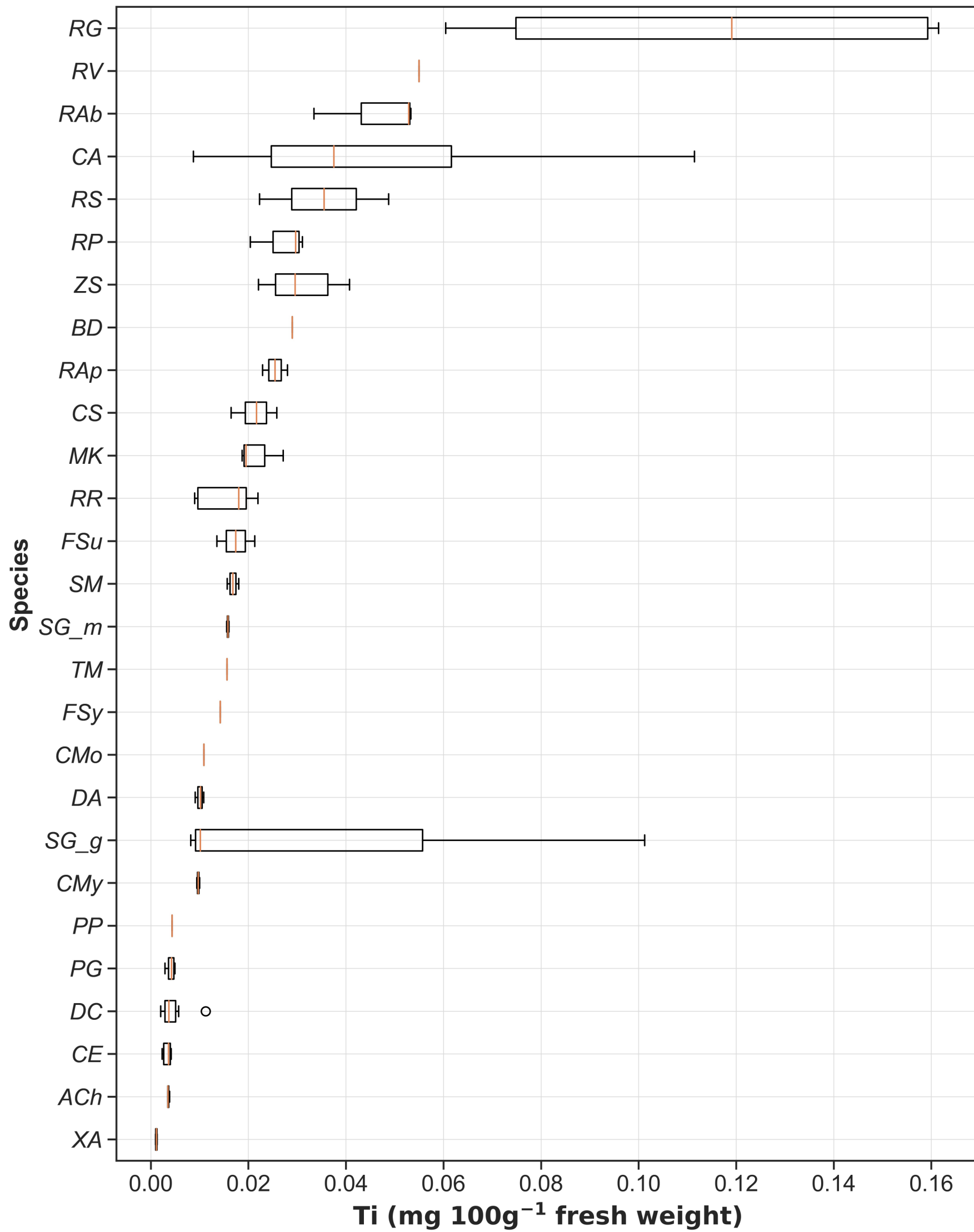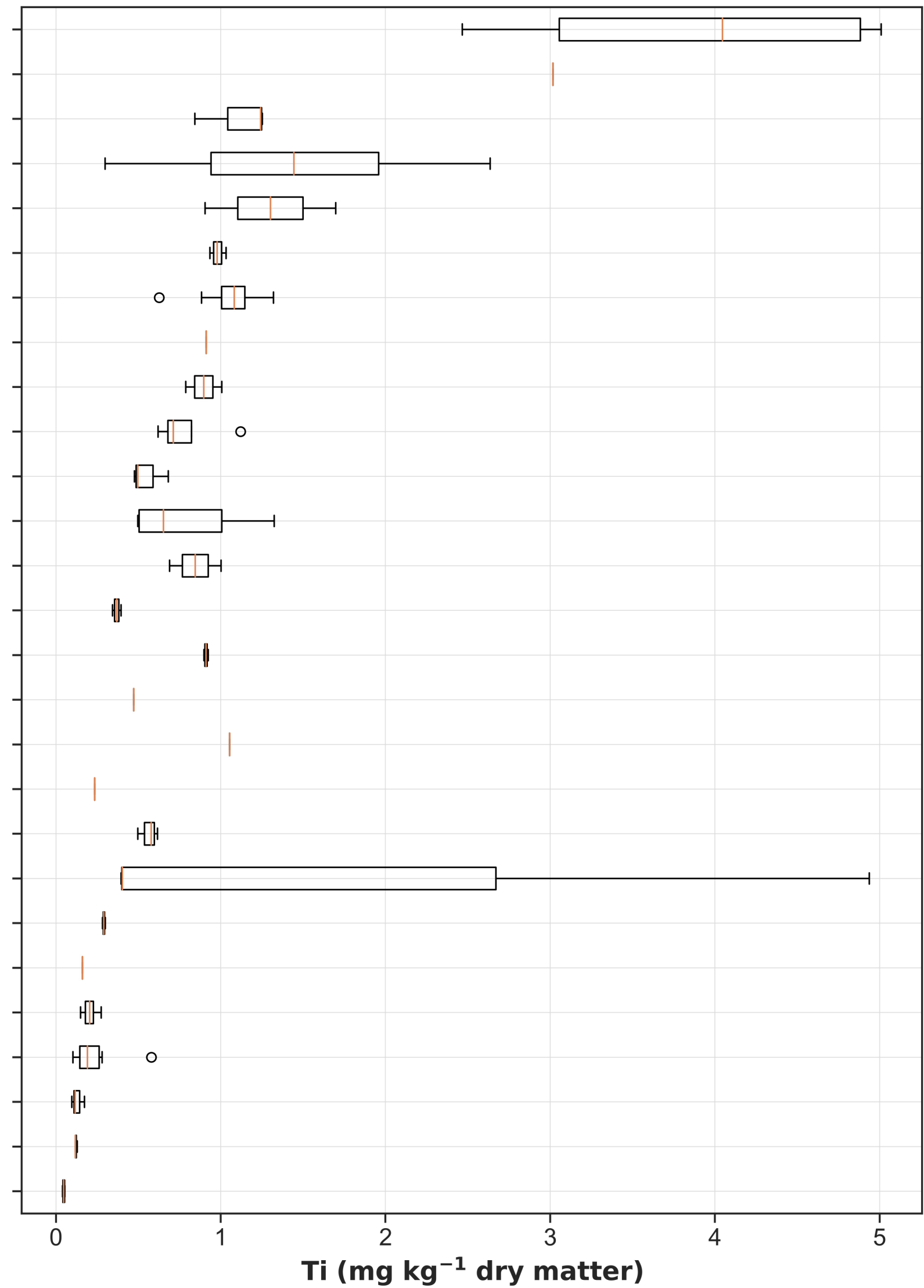

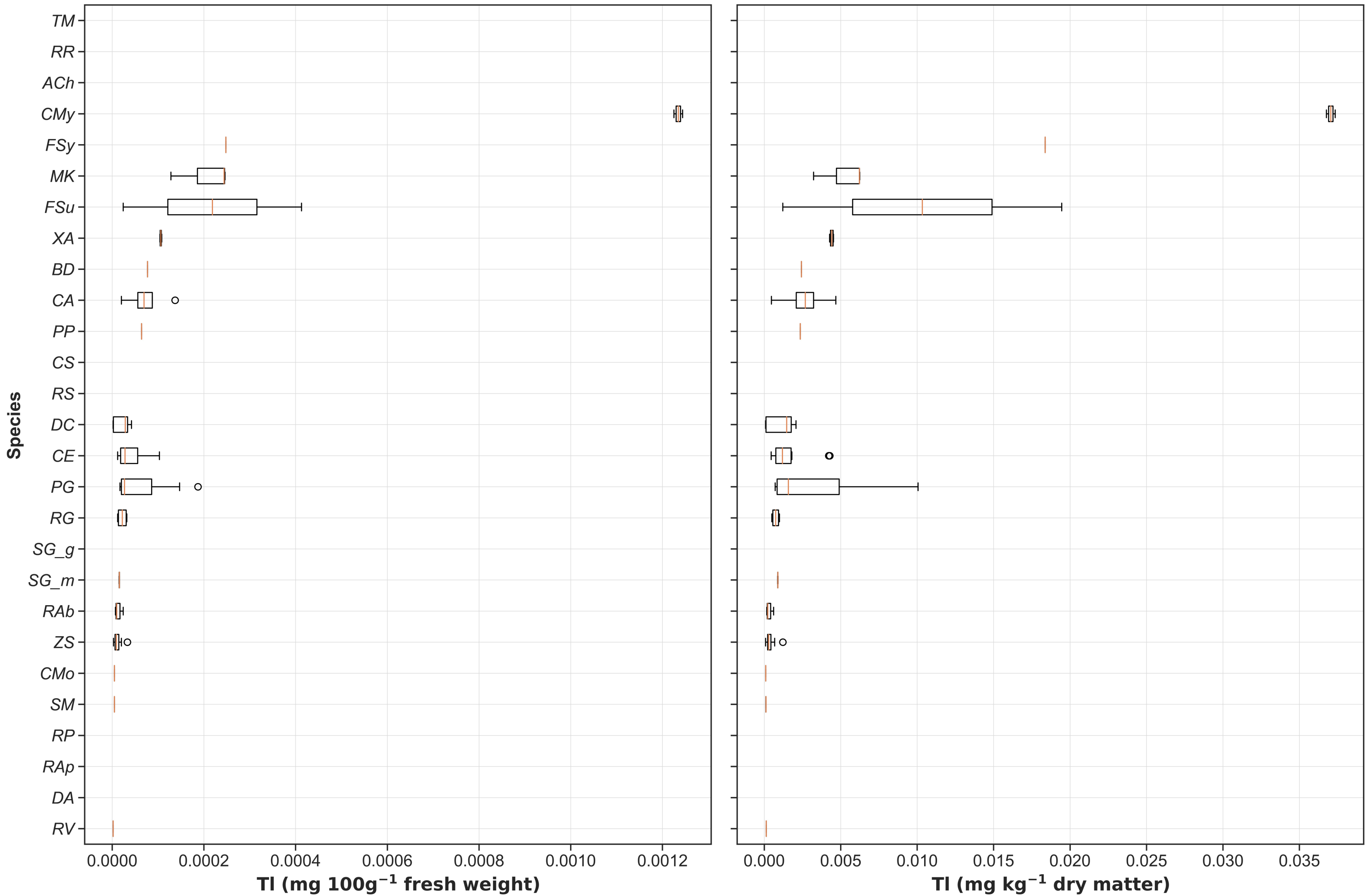

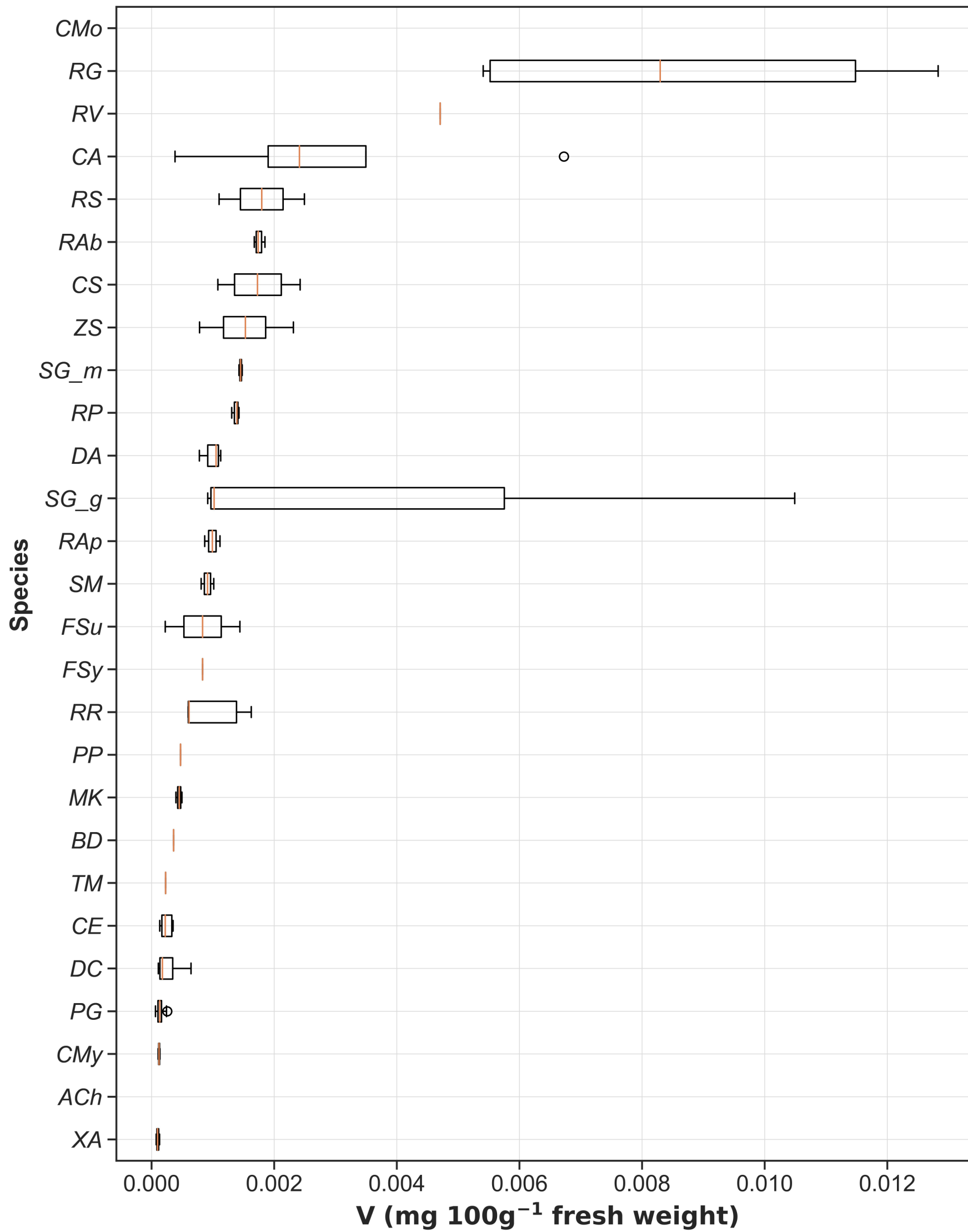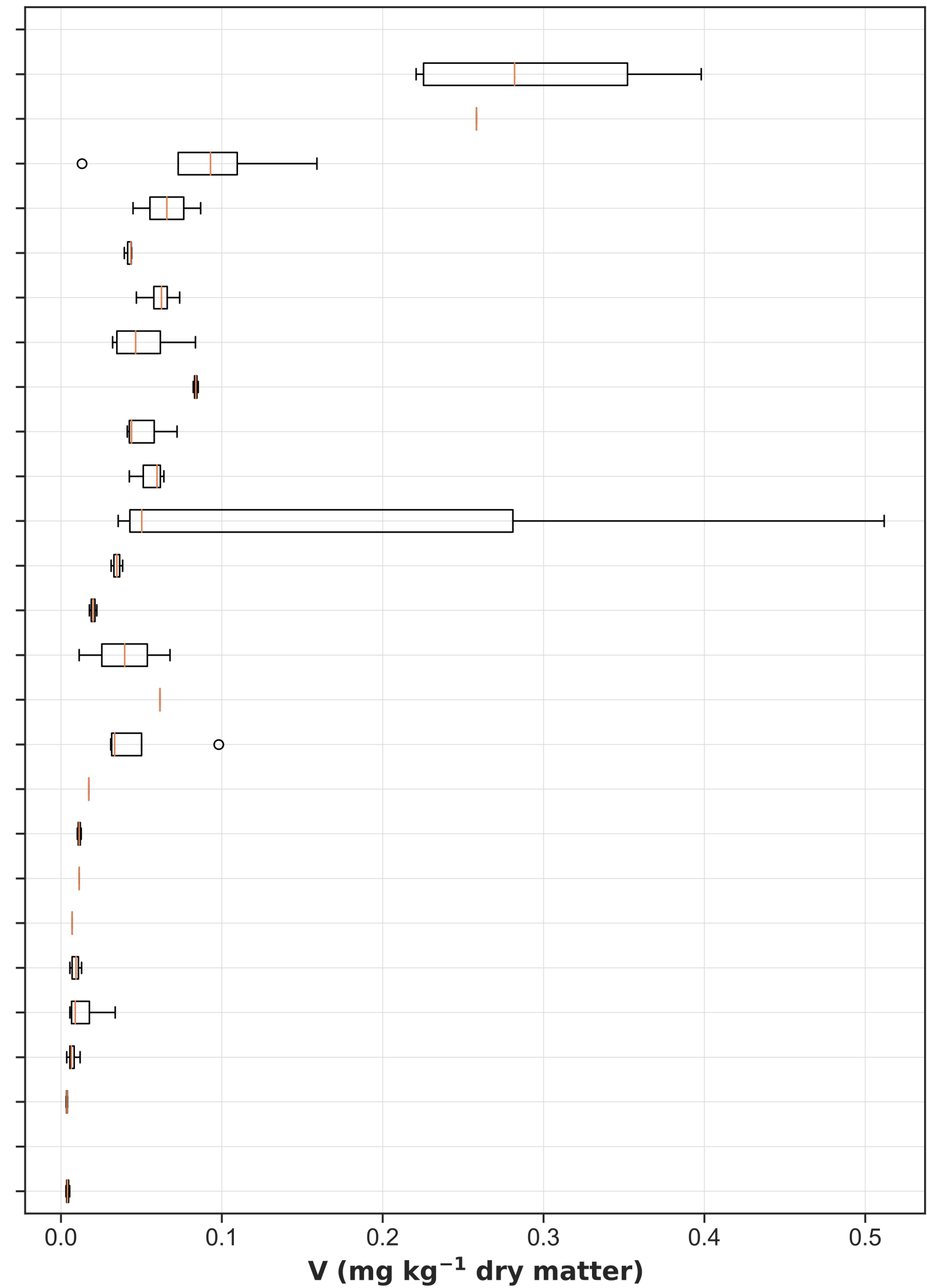

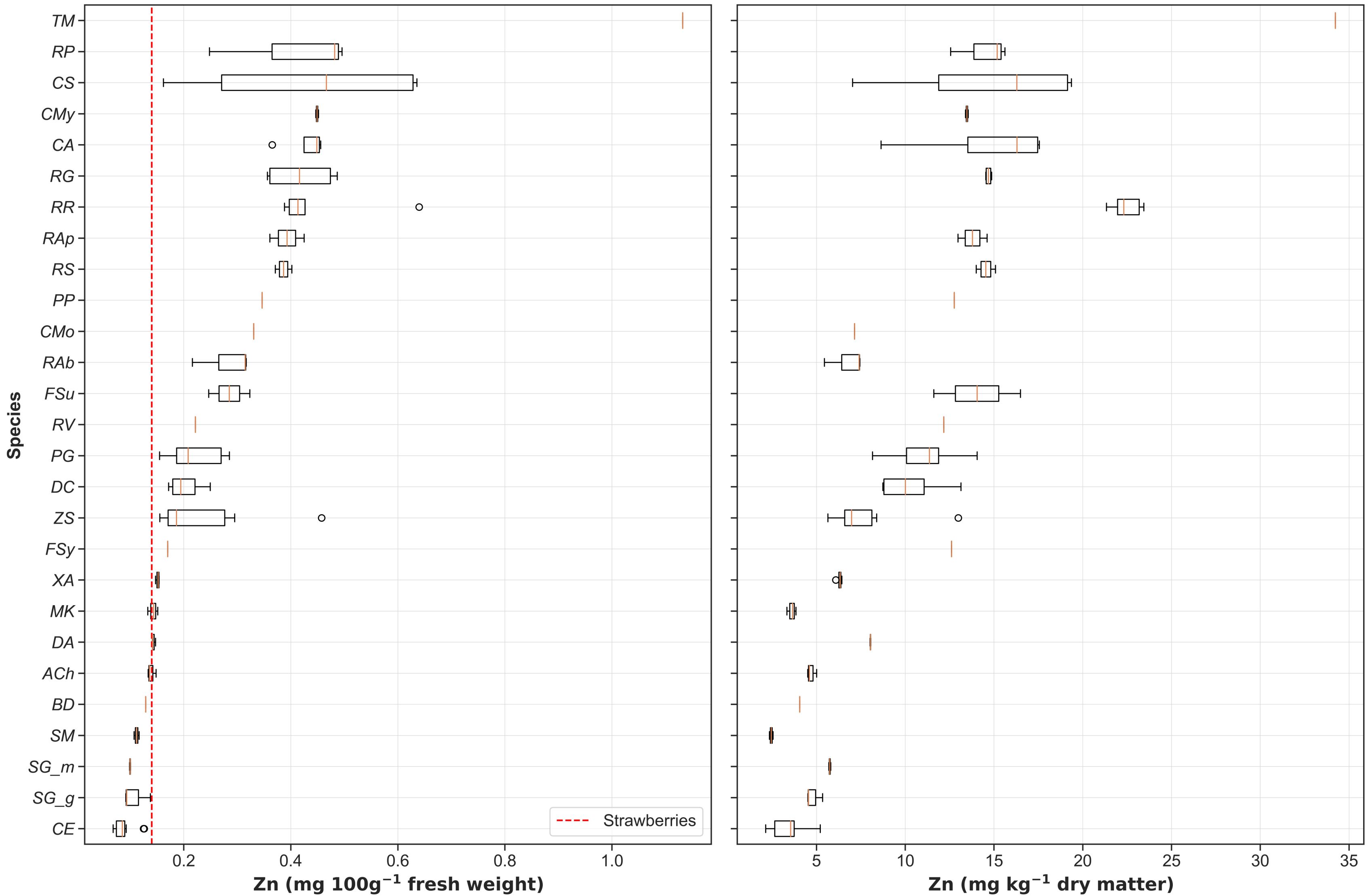

Supplement: Supplementary file 5 — Supplementary Material 5 [file 41598_2025_26400_MOESM5_ESM.pdf]

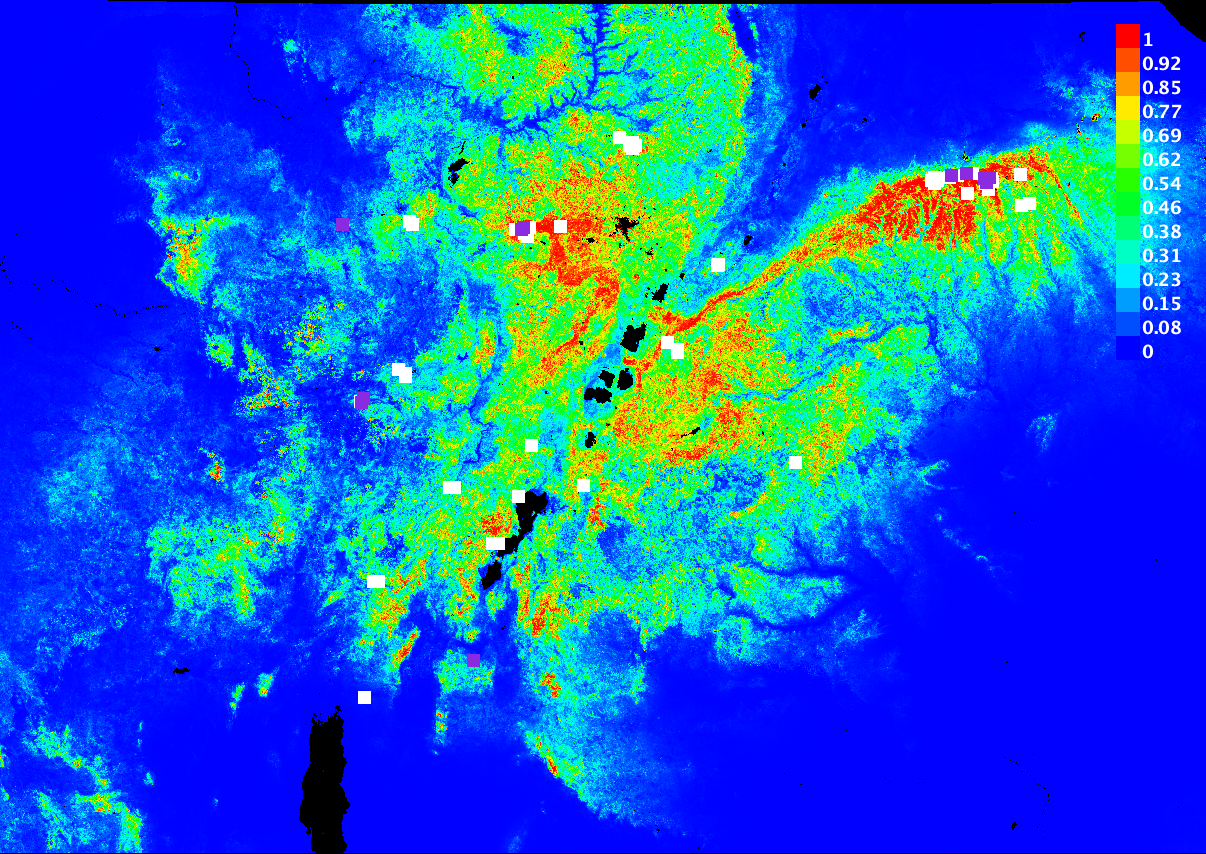

Supplement: Supplementary file 6 — Supplementary Material 6 [file 41598_2025_26400_MOESM6_ESM.zip › Supplementary file 1/plots/Carissa_spinarum.png]

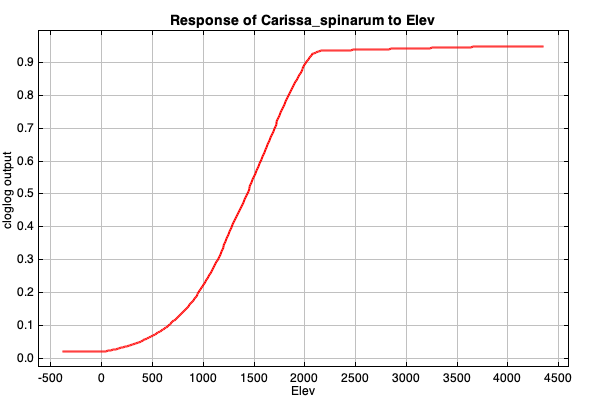

Supplement: Supplementary file 6 — Supplementary Material 6 [file 41598_2025_26400_MOESM6_ESM.zip › Supplementary file 1/plots/Carissa_spinarum_Elev.png]

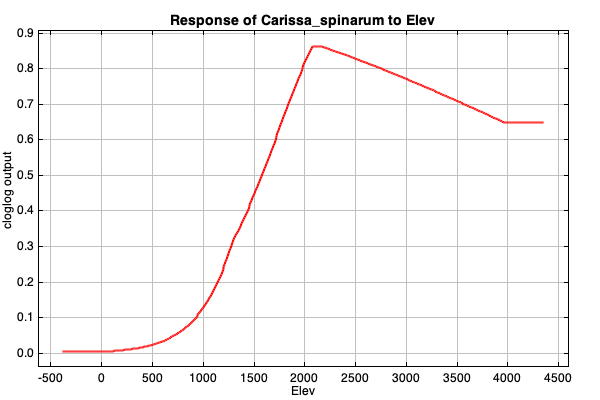

Supplement: Supplementary file 6 — Supplementary Material 6 [file 41598_2025_26400_MOESM6_ESM.zip › Supplementary file 1/plots/Carissa_spinarum_Elev_only.png]

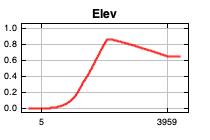

Supplement: Supplementary file 6 — Supplementary Material 6 [file 41598_2025_26400_MOESM6_ESM.zip › Supplementary file 1/plots/Carissa_spinarum_Elev_only_thumb.png]

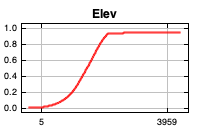

Supplement: Supplementary file 6 — Supplementary Material 6 [file 41598_2025_26400_MOESM6_ESM.zip › Supplementary file 1/plots/Carissa_spinarum_Elev_thumb.png]

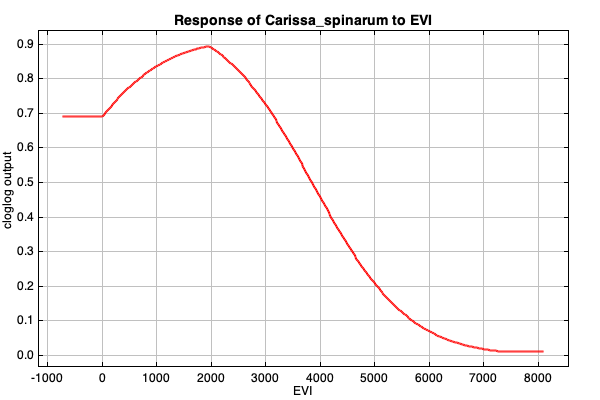

Supplement: Supplementary file 6 — Supplementary Material 6 [file 41598_2025_26400_MOESM6_ESM.zip › Supplementary file 1/plots/Carissa_spinarum_EVI.png]

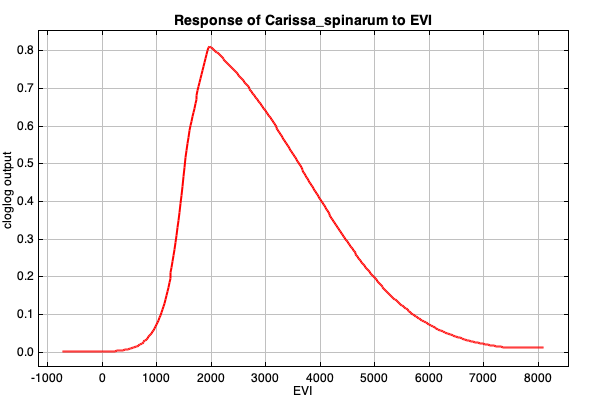

Supplement: Supplementary file 6 — Supplementary Material 6 [file 41598_2025_26400_MOESM6_ESM.zip › Supplementary file 1/plots/Carissa_spinarum_EVI_only.png]

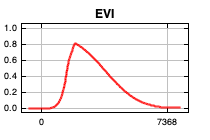

Supplement: Supplementary file 6 — Supplementary Material 6 [file 41598_2025_26400_MOESM6_ESM.zip › Supplementary file 1/plots/Carissa_spinarum_EVI_only_thumb.png]

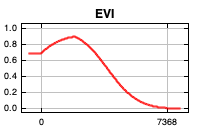

Supplement: Supplementary file 6 — Supplementary Material 6 [file 41598_2025_26400_MOESM6_ESM.zip › Supplementary file 1/plots/Carissa_spinarum_EVI_thumb.png]

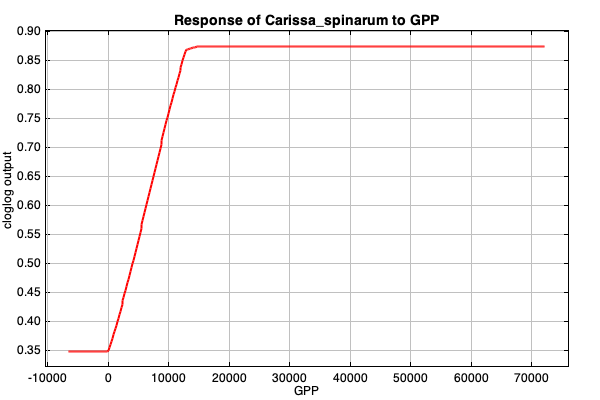

Supplement: Supplementary file 6 — Supplementary Material 6 [file 41598_2025_26400_MOESM6_ESM.zip › Supplementary file 1/plots/Carissa_spinarum_GPP.png]

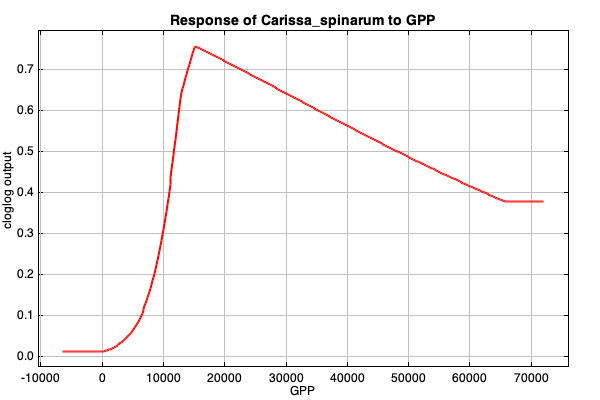

Supplement: Supplementary file 6 — Supplementary Material 6 [file 41598_2025_26400_MOESM6_ESM.zip › Supplementary file 1/plots/Carissa_spinarum_GPP_only.png]

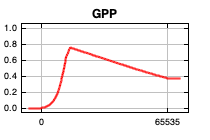

Supplement: Supplementary file 6 — Supplementary Material 6 [file 41598_2025_26400_MOESM6_ESM.zip › Supplementary file 1/plots/Carissa_spinarum_GPP_only_thumb.png]

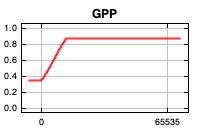

Supplement: Supplementary file 6 — Supplementary Material 6 [file 41598_2025_26400_MOESM6_ESM.zip › Supplementary file 1/plots/Carissa_spinarum_GPP_thumb.png]

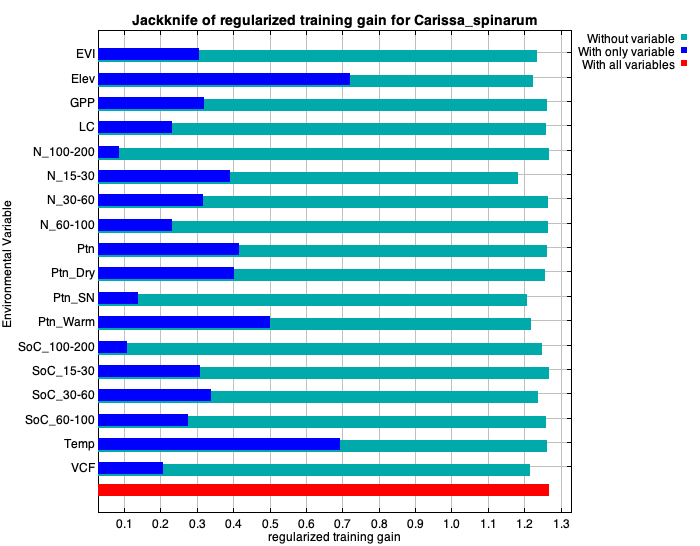

Supplement: Supplementary file 6 — Supplementary Material 6 [file 41598_2025_26400_MOESM6_ESM.zip › Supplementary file 1/plots/Carissa_spinarum_jacknife.png]

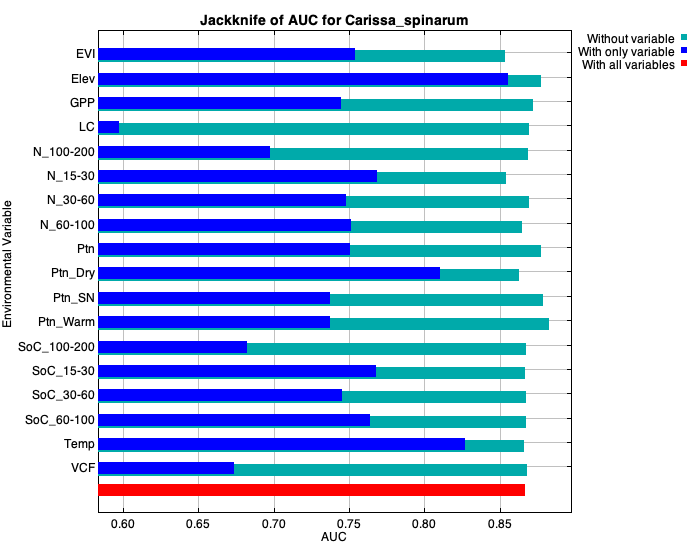

Supplement: Supplementary file 6 — Supplementary Material 6 [file 41598_2025_26400_MOESM6_ESM.zip › Supplementary file 1/plots/Carissa_spinarum_jacknife_auc.png]

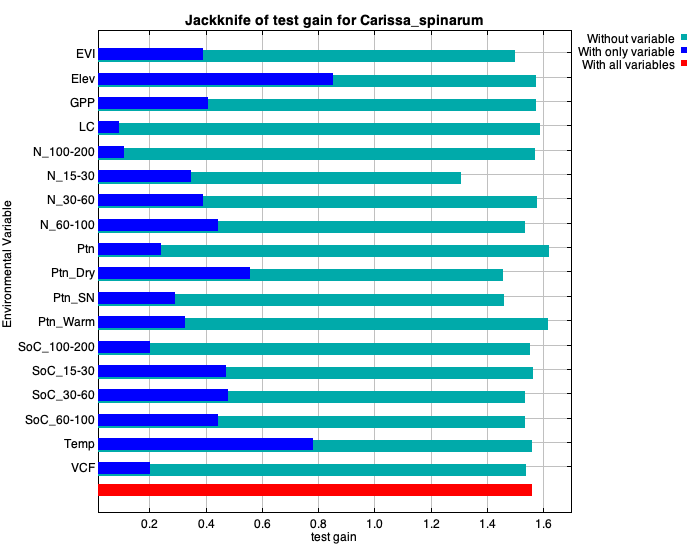

Supplement: Supplementary file 6 — Supplementary Material 6 [file 41598_2025_26400_MOESM6_ESM.zip › Supplementary file 1/plots/Carissa_spinarum_jacknife_test.png]

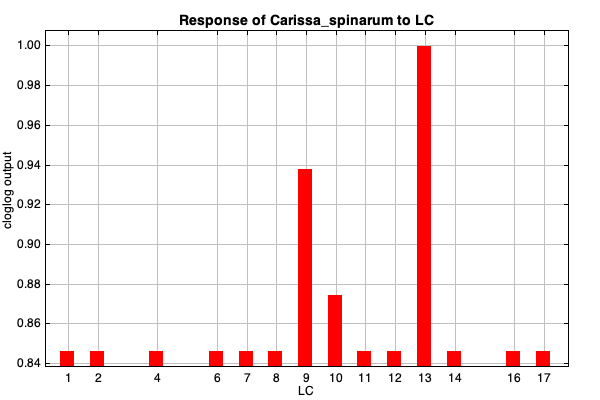

Supplement: Supplementary file 6 — Supplementary Material 6 [file 41598_2025_26400_MOESM6_ESM.zip › Supplementary file 1/plots/Carissa_spinarum_LC.png]

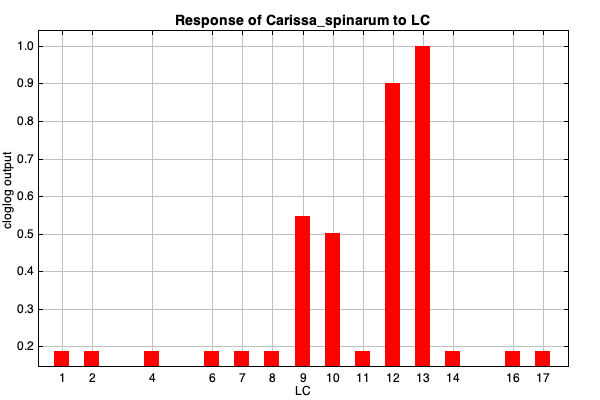

Supplement: Supplementary file 6 — Supplementary Material 6 [file 41598_2025_26400_MOESM6_ESM.zip › Supplementary file 1/plots/Carissa_spinarum_LC_only.png]

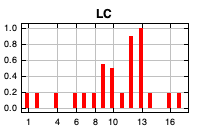

Supplement: Supplementary file 6 — Supplementary Material 6 [file 41598_2025_26400_MOESM6_ESM.zip › Supplementary file 1/plots/Carissa_spinarum_LC_only_thumb.png]

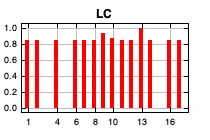

Supplement: Supplementary file 6 — Supplementary Material 6 [file 41598_2025_26400_MOESM6_ESM.zip › Supplementary file 1/plots/Carissa_spinarum_LC_thumb.png]

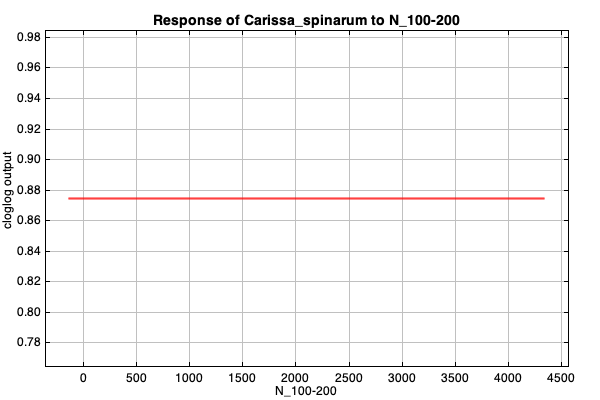

Supplement: Supplementary file 6 — Supplementary Material 6 [file 41598_2025_26400_MOESM6_ESM.zip › Supplementary file 1/plots/Carissa_spinarum_N_100-200.png]

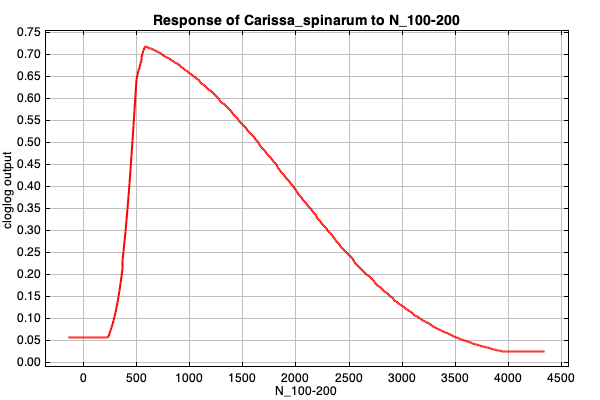

Supplement: Supplementary file 6 — Supplementary Material 6 [file 41598_2025_26400_MOESM6_ESM.zip › Supplementary file 1/plots/Carissa_spinarum_N_100-200_only.png]

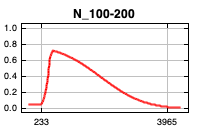

Supplement: Supplementary file 6 — Supplementary Material 6 [file 41598_2025_26400_MOESM6_ESM.zip › Supplementary file 1/plots/Carissa_spinarum_N_100-200_only_thumb.png]

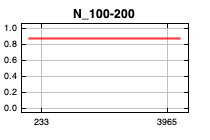

Supplement: Supplementary file 6 — Supplementary Material 6 [file 41598_2025_26400_MOESM6_ESM.zip › Supplementary file 1/plots/Carissa_spinarum_N_100-200_thumb.png]

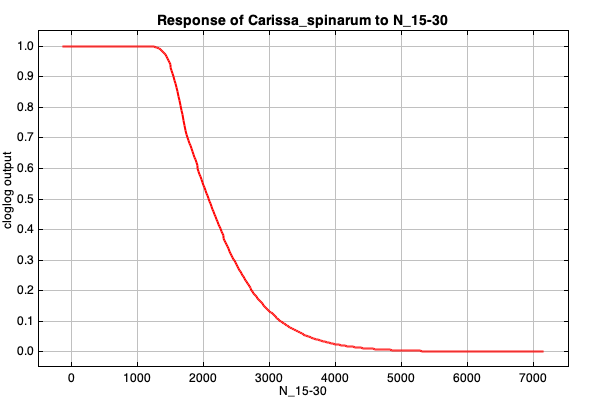

Supplement: Supplementary file 6 — Supplementary Material 6 [file 41598_2025_26400_MOESM6_ESM.zip › Supplementary file 1/plots/Carissa_spinarum_N_15-30.png]

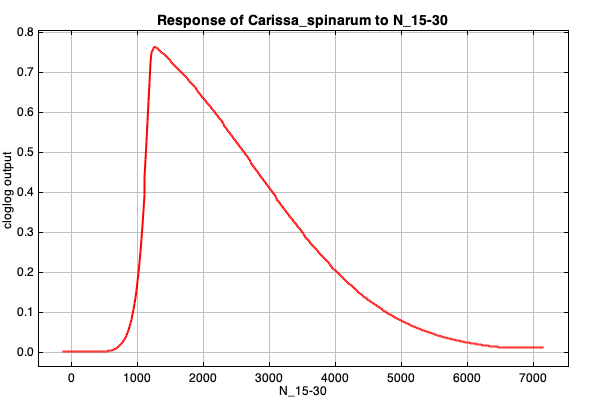

Supplement: Supplementary file 6 — Supplementary Material 6 [file 41598_2025_26400_MOESM6_ESM.zip › Supplementary file 1/plots/Carissa_spinarum_N_15-30_only.png]

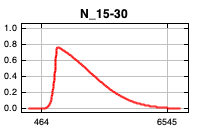

Supplement: Supplementary file 6 — Supplementary Material 6 [file 41598_2025_26400_MOESM6_ESM.zip › Supplementary file 1/plots/Carissa_spinarum_N_15-30_only_thumb.png]

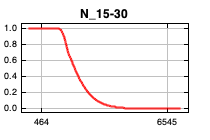

Supplement: Supplementary file 6 — Supplementary Material 6 [file 41598_2025_26400_MOESM6_ESM.zip › Supplementary file 1/plots/Carissa_spinarum_N_15-30_thumb.png]

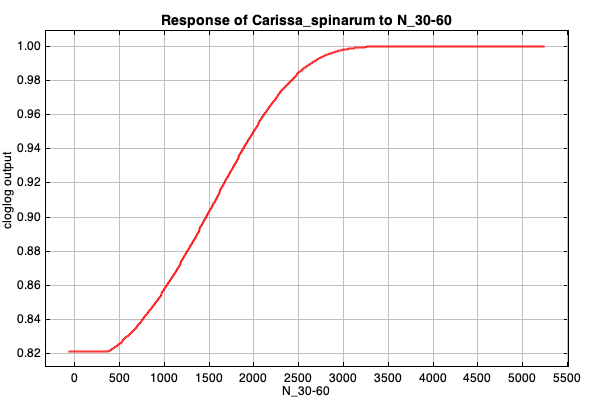

Supplement: Supplementary file 6 — Supplementary Material 6 [file 41598_2025_26400_MOESM6_ESM.zip › Supplementary file 1/plots/Carissa_spinarum_N_30-60.png]

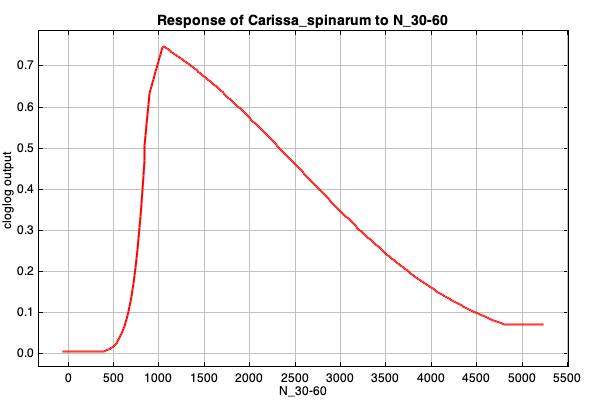

Supplement: Supplementary file 6 — Supplementary Material 6 [file 41598_2025_26400_MOESM6_ESM.zip › Supplementary file 1/plots/Carissa_spinarum_N_30-60_only.png]

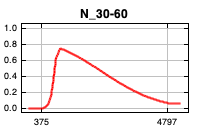

Supplement: Supplementary file 6 — Supplementary Material 6 [file 41598_2025_26400_MOESM6_ESM.zip › Supplementary file 1/plots/Carissa_spinarum_N_30-60_only_thumb.png]

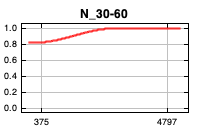

Supplement: Supplementary file 6 — Supplementary Material 6 [file 41598_2025_26400_MOESM6_ESM.zip › Supplementary file 1/plots/Carissa_spinarum_N_30-60_thumb.png]

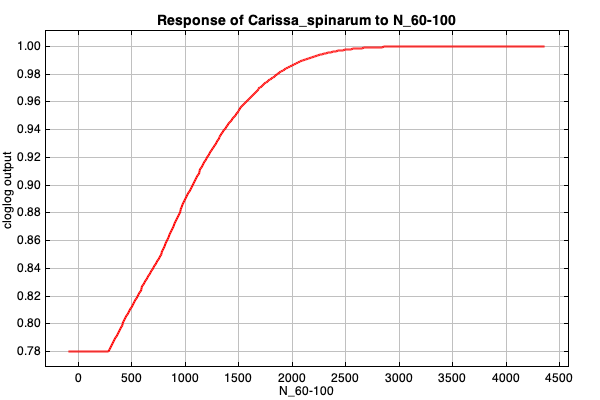

Supplement: Supplementary file 6 — Supplementary Material 6 [file 41598_2025_26400_MOESM6_ESM.zip › Supplementary file 1/plots/Carissa_spinarum_N_60-100.png]

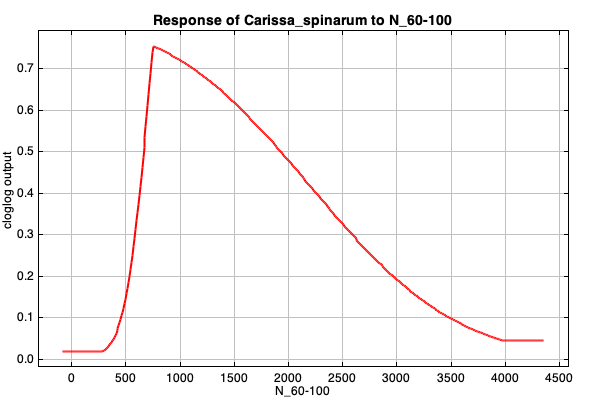

Supplement: Supplementary file 6 — Supplementary Material 6 [file 41598_2025_26400_MOESM6_ESM.zip › Supplementary file 1/plots/Carissa_spinarum_N_60-100_only.png]

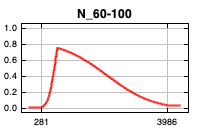

Supplement: Supplementary file 6 — Supplementary Material 6 [file 41598_2025_26400_MOESM6_ESM.zip › Supplementary file 1/plots/Carissa_spinarum_N_60-100_only_thumb.png]

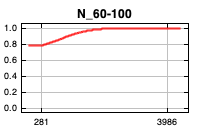

Supplement: Supplementary file 6 — Supplementary Material 6 [file 41598_2025_26400_MOESM6_ESM.zip › Supplementary file 1/plots/Carissa_spinarum_N_60-100_thumb.png]

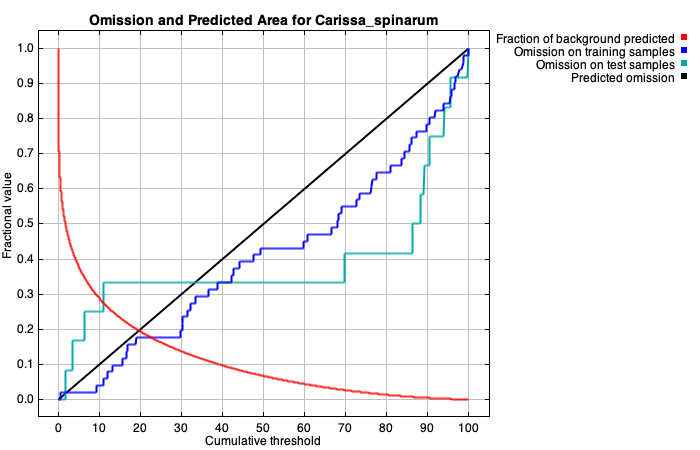

Supplement: Supplementary file 6 — Supplementary Material 6 [file 41598_2025_26400_MOESM6_ESM.zip › Supplementary file 1/plots/Carissa_spinarum_omission.png]

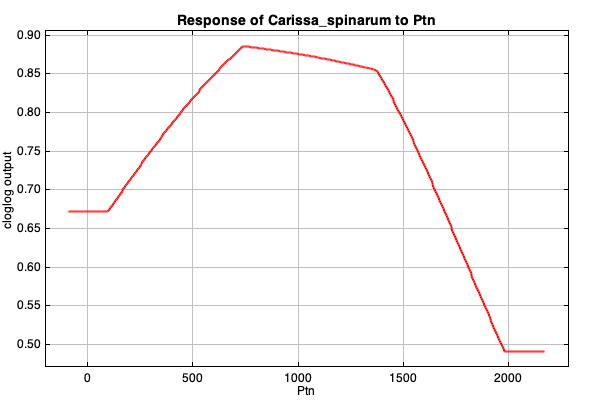

Supplement: Supplementary file 6 — Supplementary Material 6 [file 41598_2025_26400_MOESM6_ESM.zip › Supplementary file 1/plots/Carissa_spinarum_Ptn.png]

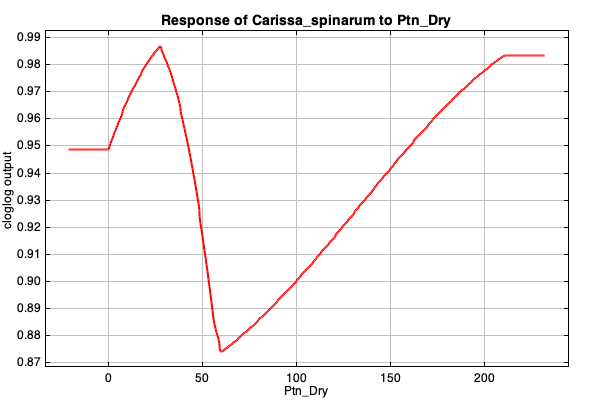

Supplement: Supplementary file 6 — Supplementary Material 6 [file 41598_2025_26400_MOESM6_ESM.zip › Supplementary file 1/plots/Carissa_spinarum_Ptn_Dry.png]

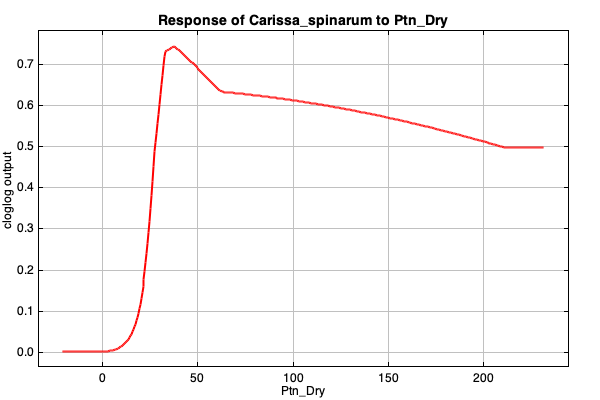

Supplement: Supplementary file 6 — Supplementary Material 6 [file 41598_2025_26400_MOESM6_ESM.zip › Supplementary file 1/plots/Carissa_spinarum_Ptn_Dry_only.png]

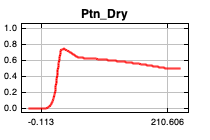

Supplement: Supplementary file 6 — Supplementary Material 6 [file 41598_2025_26400_MOESM6_ESM.zip › Supplementary file 1/plots/Carissa_spinarum_Ptn_Dry_only_thumb.png]

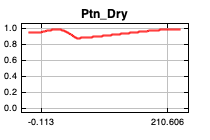

Supplement: Supplementary file 6 — Supplementary Material 6 [file 41598_2025_26400_MOESM6_ESM.zip › Supplementary file 1/plots/Carissa_spinarum_Ptn_Dry_thumb.png]

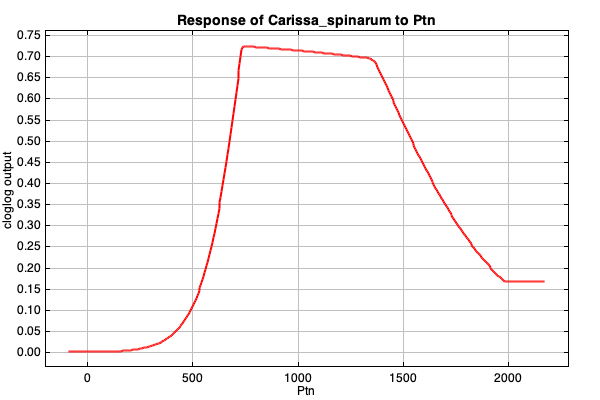

Supplement: Supplementary file 6 — Supplementary Material 6 [file 41598_2025_26400_MOESM6_ESM.zip › Supplementary file 1/plots/Carissa_spinarum_Ptn_only.png]

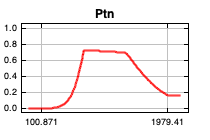

Supplement: Supplementary file 6 — Supplementary Material 6 [file 41598_2025_26400_MOESM6_ESM.zip › Supplementary file 1/plots/Carissa_spinarum_Ptn_only_thumb.png]

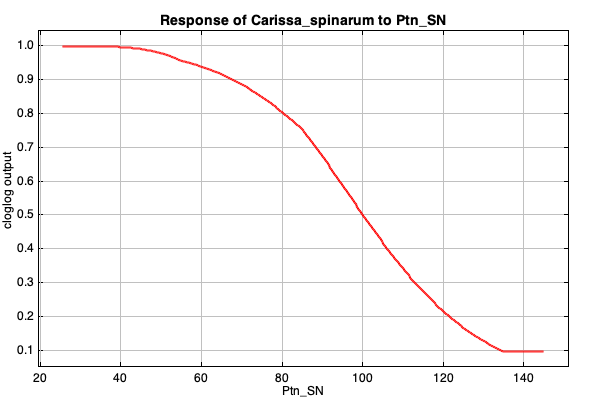

Supplement: Supplementary file 6 — Supplementary Material 6 [file 41598_2025_26400_MOESM6_ESM.zip › Supplementary file 1/plots/Carissa_spinarum_Ptn_SN.png]

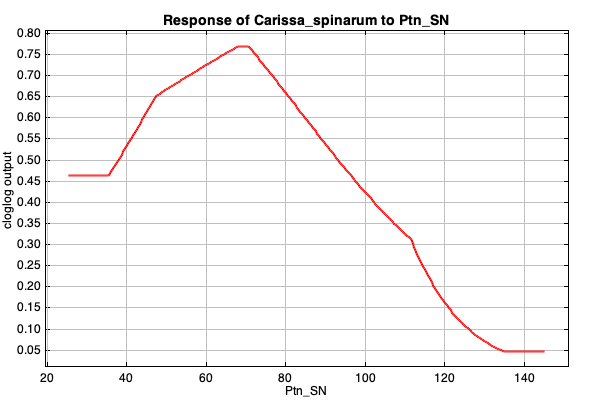

Supplement: Supplementary file 6 — Supplementary Material 6 [file 41598_2025_26400_MOESM6_ESM.zip › Supplementary file 1/plots/Carissa_spinarum_Ptn_SN_only.png]

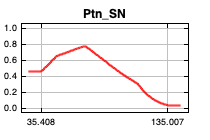

Supplement: Supplementary file 6 — Supplementary Material 6 [file 41598_2025_26400_MOESM6_ESM.zip › Supplementary file 1/plots/Carissa_spinarum_Ptn_SN_only_thumb.png]

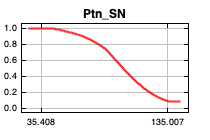

Supplement: Supplementary file 6 — Supplementary Material 6 [file 41598_2025_26400_MOESM6_ESM.zip › Supplementary file 1/plots/Carissa_spinarum_Ptn_SN_thumb.png]

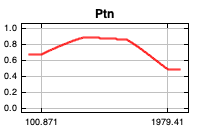

Supplement: Supplementary file 6 — Supplementary Material 6 [file 41598_2025_26400_MOESM6_ESM.zip › Supplementary file 1/plots/Carissa_spinarum_Ptn_thumb.png]

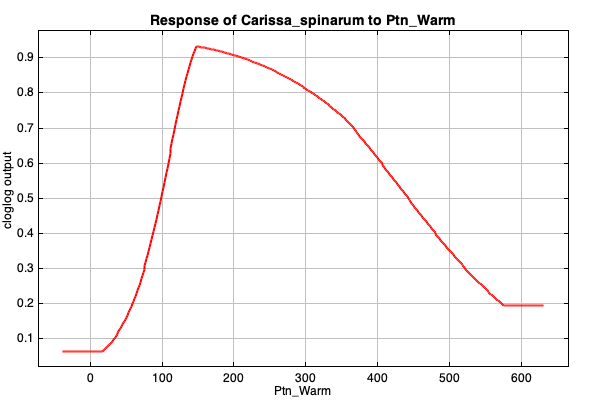

Supplement: Supplementary file 6 — Supplementary Material 6 [file 41598_2025_26400_MOESM6_ESM.zip › Supplementary file 1/plots/Carissa_spinarum_Ptn_Warm.png]

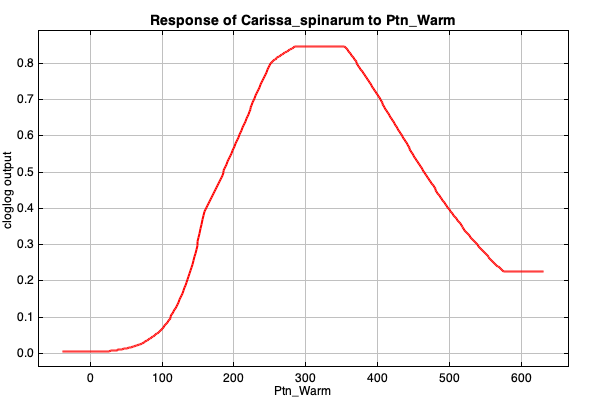

Supplement: Supplementary file 6 — Supplementary Material 6 [file 41598_2025_26400_MOESM6_ESM.zip › Supplementary file 1/plots/Carissa_spinarum_Ptn_Warm_only.png]

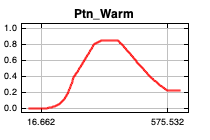

Supplement: Supplementary file 6 — Supplementary Material 6 [file 41598_2025_26400_MOESM6_ESM.zip › Supplementary file 1/plots/Carissa_spinarum_Ptn_Warm_only_thumb.png]

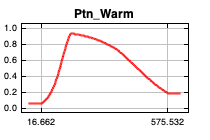

Supplement: Supplementary file 6 — Supplementary Material 6 [file 41598_2025_26400_MOESM6_ESM.zip › Supplementary file 1/plots/Carissa_spinarum_Ptn_Warm_thumb.png]

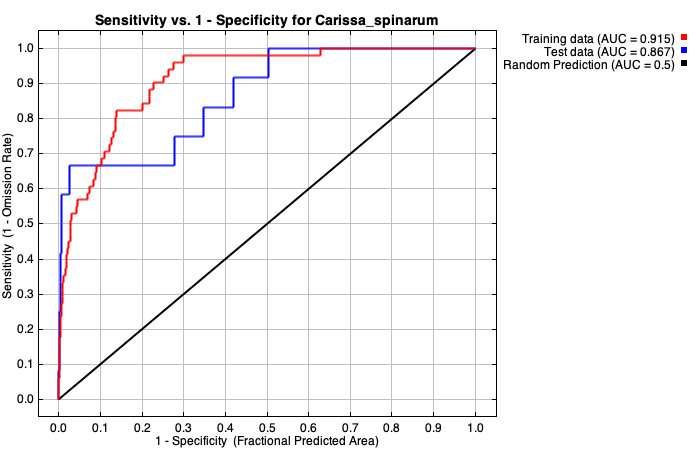

Supplement: Supplementary file 6 — Supplementary Material 6 [file 41598_2025_26400_MOESM6_ESM.zip › Supplementary file 1/plots/Carissa_spinarum_roc.png]

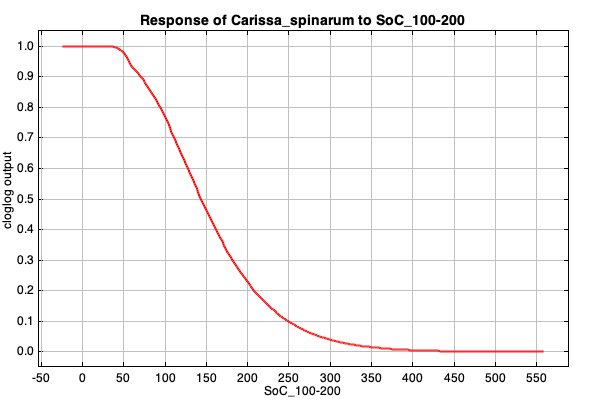

Supplement: Supplementary file 6 — Supplementary Material 6 [file 41598_2025_26400_MOESM6_ESM.zip › Supplementary file 1/plots/Carissa_spinarum_SoC_100-200.png]

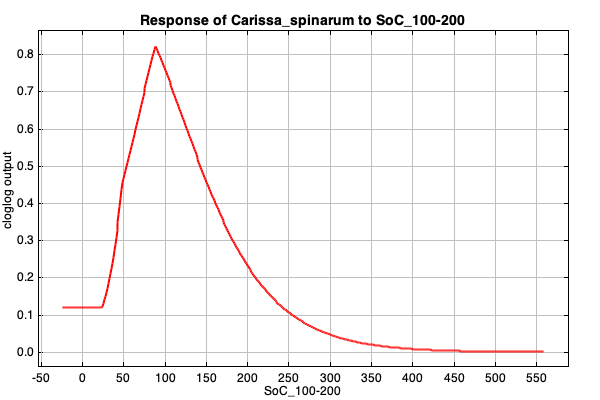

Supplement: Supplementary file 6 — Supplementary Material 6 [file 41598_2025_26400_MOESM6_ESM.zip › Supplementary file 1/plots/Carissa_spinarum_SoC_100-200_only.png]

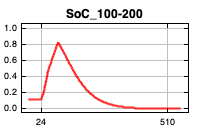

Supplement: Supplementary file 6 — Supplementary Material 6 [file 41598_2025_26400_MOESM6_ESM.zip › Supplementary file 1/plots/Carissa_spinarum_SoC_100-200_only_thumb.png]

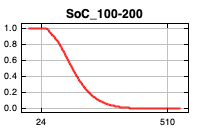

Supplement: Supplementary file 6 — Supplementary Material 6 [file 41598_2025_26400_MOESM6_ESM.zip › Supplementary file 1/plots/Carissa_spinarum_SoC_100-200_thumb.png]

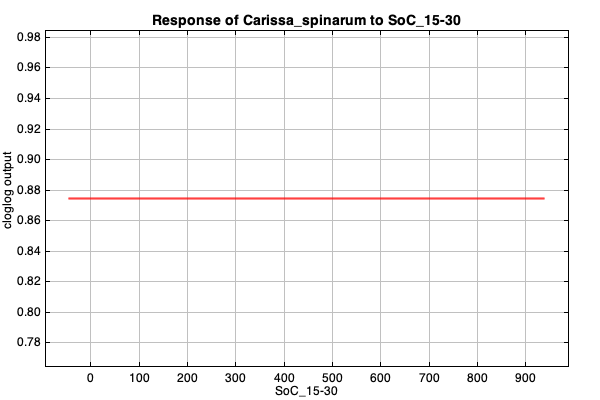

Supplement: Supplementary file 6 — Supplementary Material 6 [file 41598_2025_26400_MOESM6_ESM.zip › Supplementary file 1/plots/Carissa_spinarum_SoC_15-30.png]

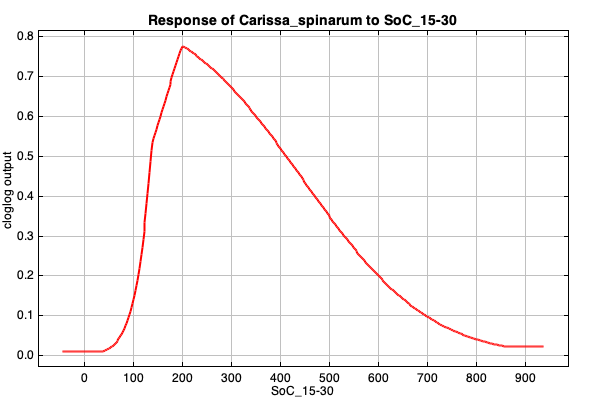

Supplement: Supplementary file 6 — Supplementary Material 6 [file 41598_2025_26400_MOESM6_ESM.zip › Supplementary file 1/plots/Carissa_spinarum_SoC_15-30_only.png]

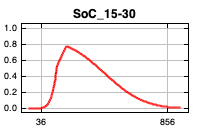

Supplement: Supplementary file 6 — Supplementary Material 6 [file 41598_2025_26400_MOESM6_ESM.zip › Supplementary file 1/plots/Carissa_spinarum_SoC_15-30_only_thumb.png]

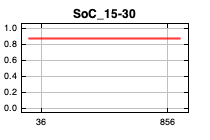

Supplement: Supplementary file 6 — Supplementary Material 6 [file 41598_2025_26400_MOESM6_ESM.zip › Supplementary file 1/plots/Carissa_spinarum_SoC_15-30_thumb.png]

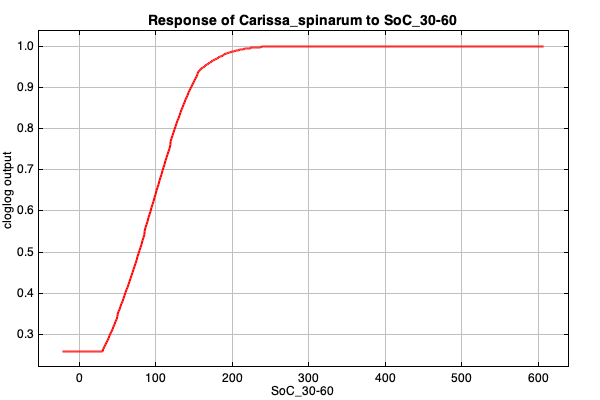

Supplement: Supplementary file 6 — Supplementary Material 6 [file 41598_2025_26400_MOESM6_ESM.zip › Supplementary file 1/plots/Carissa_spinarum_SoC_30-60.png]

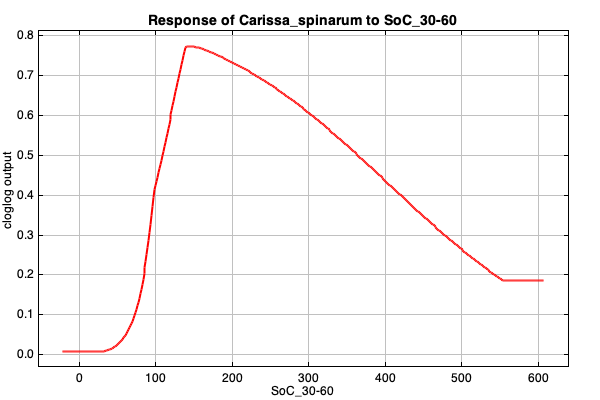

Supplement: Supplementary file 6 — Supplementary Material 6 [file 41598_2025_26400_MOESM6_ESM.zip › Supplementary file 1/plots/Carissa_spinarum_SoC_30-60_only.png]

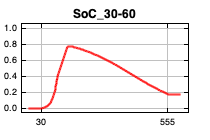

Supplement: Supplementary file 6 — Supplementary Material 6 [file 41598_2025_26400_MOESM6_ESM.zip › Supplementary file 1/plots/Carissa_spinarum_SoC_30-60_only_thumb.png]

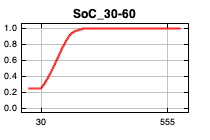

Supplement: Supplementary file 6 — Supplementary Material 6 [file 41598_2025_26400_MOESM6_ESM.zip › Supplementary file 1/plots/Carissa_spinarum_SoC_30-60_thumb.png]

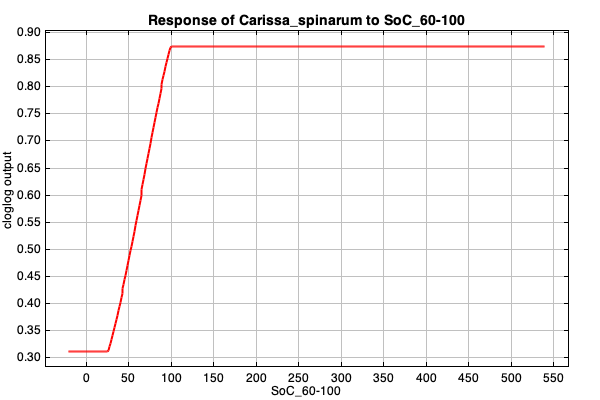

Supplement: Supplementary file 6 — Supplementary Material 6 [file 41598_2025_26400_MOESM6_ESM.zip › Supplementary file 1/plots/Carissa_spinarum_SoC_60-100.png]

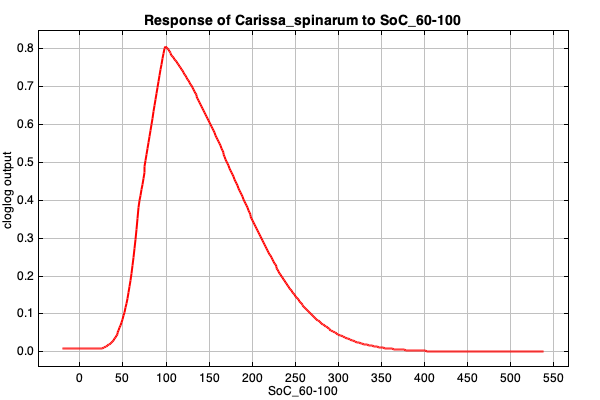

Supplement: Supplementary file 6 — Supplementary Material 6 [file 41598_2025_26400_MOESM6_ESM.zip › Supplementary file 1/plots/Carissa_spinarum_SoC_60-100_only.png]

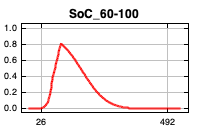

Supplement: Supplementary file 6 — Supplementary Material 6 [file 41598_2025_26400_MOESM6_ESM.zip › Supplementary file 1/plots/Carissa_spinarum_SoC_60-100_only_thumb.png]

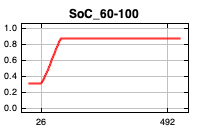

Supplement: Supplementary file 6 — Supplementary Material 6 [file 41598_2025_26400_MOESM6_ESM.zip › Supplementary file 1/plots/Carissa_spinarum_SoC_60-100_thumb.png]

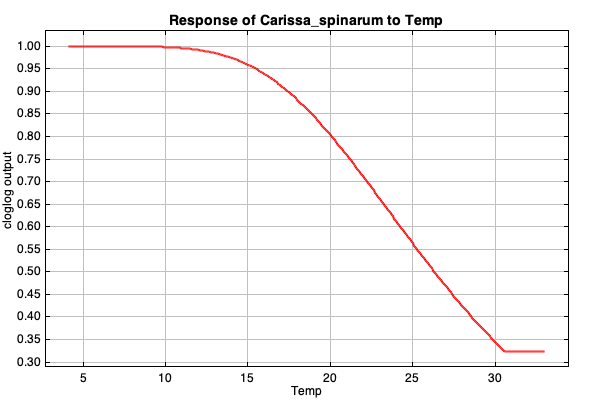

Supplement: Supplementary file 6 — Supplementary Material 6 [file 41598_2025_26400_MOESM6_ESM.zip › Supplementary file 1/plots/Carissa_spinarum_Temp.png]

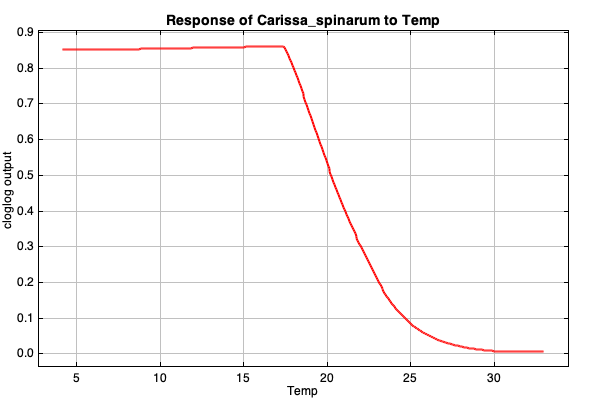

Supplement: Supplementary file 6 — Supplementary Material 6 [file 41598_2025_26400_MOESM6_ESM.zip › Supplementary file 1/plots/Carissa_spinarum_Temp_only.png]

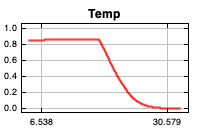

Supplement: Supplementary file 6 — Supplementary Material 6 [file 41598_2025_26400_MOESM6_ESM.zip › Supplementary file 1/plots/Carissa_spinarum_Temp_only_thumb.png]

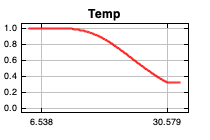

Supplement: Supplementary file 6 — Supplementary Material 6 [file 41598_2025_26400_MOESM6_ESM.zip › Supplementary file 1/plots/Carissa_spinarum_Temp_thumb.png]

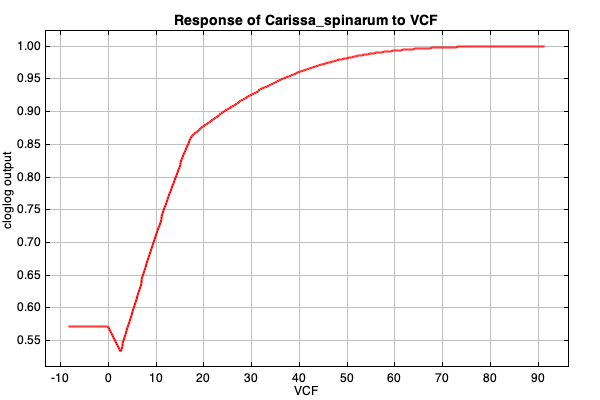

Supplement: Supplementary file 6 — Supplementary Material 6 [file 41598_2025_26400_MOESM6_ESM.zip › Supplementary file 1/plots/Carissa_spinarum_VCF.png]

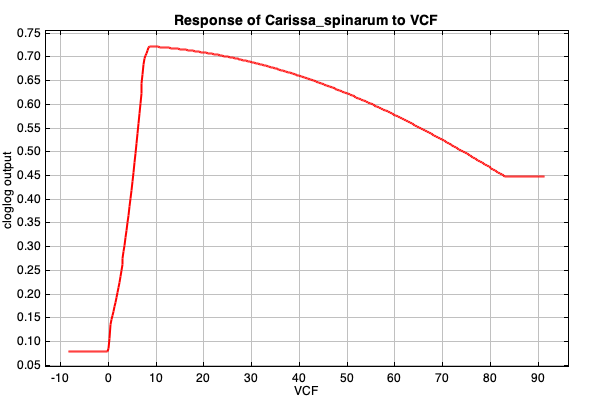

Supplement: Supplementary file 6 — Supplementary Material 6 [file 41598_2025_26400_MOESM6_ESM.zip › Supplementary file 1/plots/Carissa_spinarum_VCF_only.png]

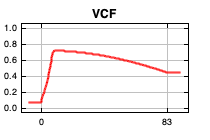

Supplement: Supplementary file 6 — Supplementary Material 6 [file 41598_2025_26400_MOESM6_ESM.zip › Supplementary file 1/plots/Carissa_spinarum_VCF_only_thumb.png]

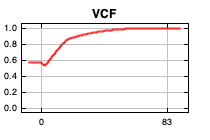

Supplement: Supplementary file 6 — Supplementary Material 6 [file 41598_2025_26400_MOESM6_ESM.zip › Supplementary file 1/plots/Carissa_spinarum_VCF_thumb.png]

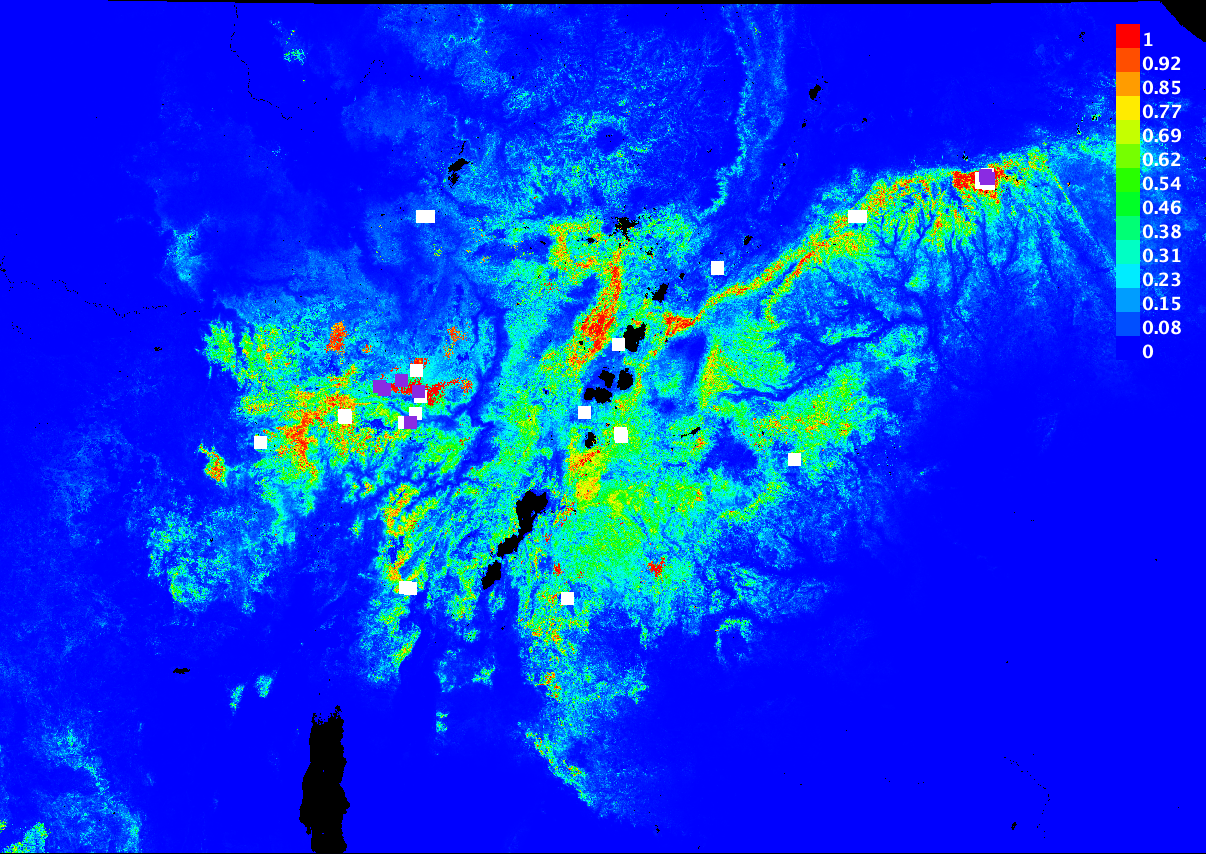

Supplement: Supplementary file 6 — Supplementary Material 6 [file 41598_2025_26400_MOESM6_ESM.zip › Supplementary file 1/plots/Cordia_africana.png]

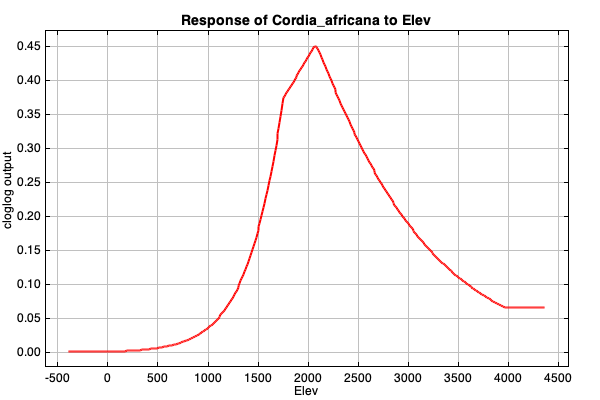

Supplement: Supplementary file 6 — Supplementary Material 6 [file 41598_2025_26400_MOESM6_ESM.zip › Supplementary file 1/plots/Cordia_africana_Elev.png]

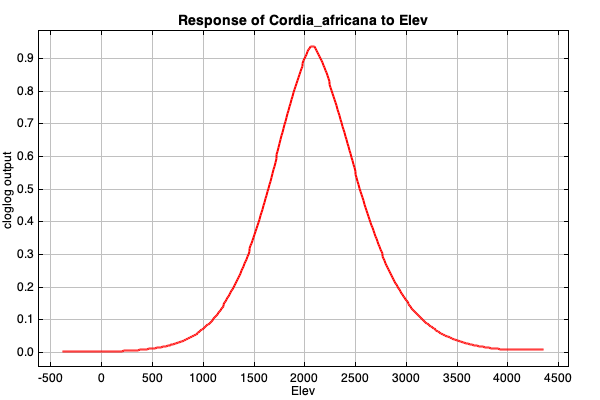

Supplement: Supplementary file 6 — Supplementary Material 6 [file 41598_2025_26400_MOESM6_ESM.zip › Supplementary file 1/plots/Cordia_africana_Elev_only.png]

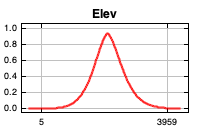

Supplement: Supplementary file 6 — Supplementary Material 6 [file 41598_2025_26400_MOESM6_ESM.zip › Supplementary file 1/plots/Cordia_africana_Elev_only_thumb.png]

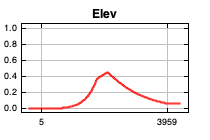

Supplement: Supplementary file 6 — Supplementary Material 6 [file 41598_2025_26400_MOESM6_ESM.zip › Supplementary file 1/plots/Cordia_africana_Elev_thumb.png]

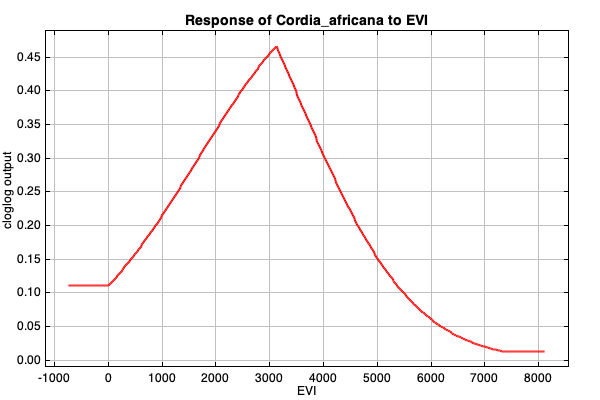

Supplement: Supplementary file 6 — Supplementary Material 6 [file 41598_2025_26400_MOESM6_ESM.zip › Supplementary file 1/plots/Cordia_africana_EVI.png]

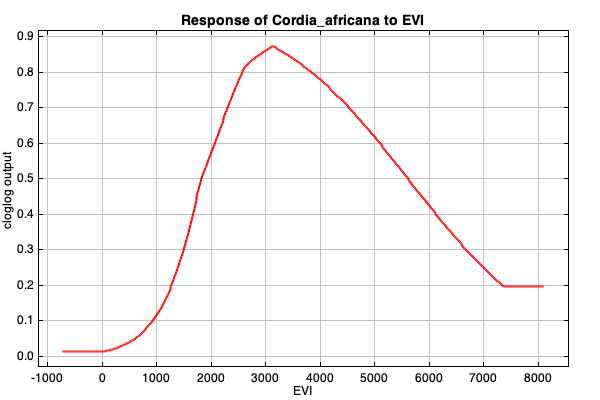

Supplement: Supplementary file 6 — Supplementary Material 6 [file 41598_2025_26400_MOESM6_ESM.zip › Supplementary file 1/plots/Cordia_africana_EVI_only.png]

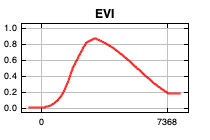

Supplement: Supplementary file 6 — Supplementary Material 6 [file 41598_2025_26400_MOESM6_ESM.zip › Supplementary file 1/plots/Cordia_africana_EVI_only_thumb.png]

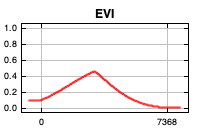

Supplement: Supplementary file 6 — Supplementary Material 6 [file 41598_2025_26400_MOESM6_ESM.zip › Supplementary file 1/plots/Cordia_africana_EVI_thumb.png]

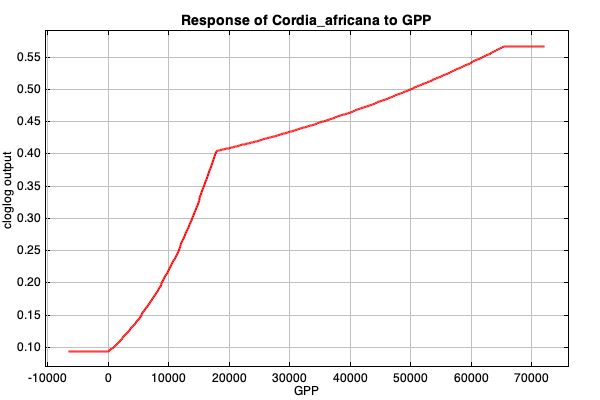

Supplement: Supplementary file 6 — Supplementary Material 6 [file 41598_2025_26400_MOESM6_ESM.zip › Supplementary file 1/plots/Cordia_africana_GPP.png]

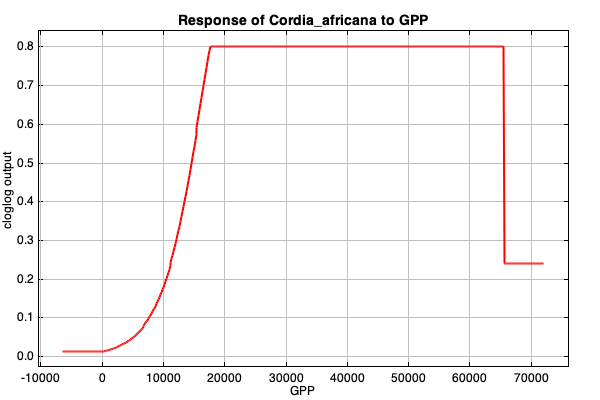

Supplement: Supplementary file 6 — Supplementary Material 6 [file 41598_2025_26400_MOESM6_ESM.zip › Supplementary file 1/plots/Cordia_africana_GPP_only.png]

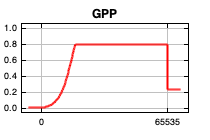

Supplement: Supplementary file 6 — Supplementary Material 6 [file 41598_2025_26400_MOESM6_ESM.zip › Supplementary file 1/plots/Cordia_africana_GPP_only_thumb.png]

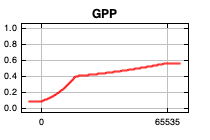

Supplement: Supplementary file 6 — Supplementary Material 6 [file 41598_2025_26400_MOESM6_ESM.zip › Supplementary file 1/plots/Cordia_africana_GPP_thumb.png]

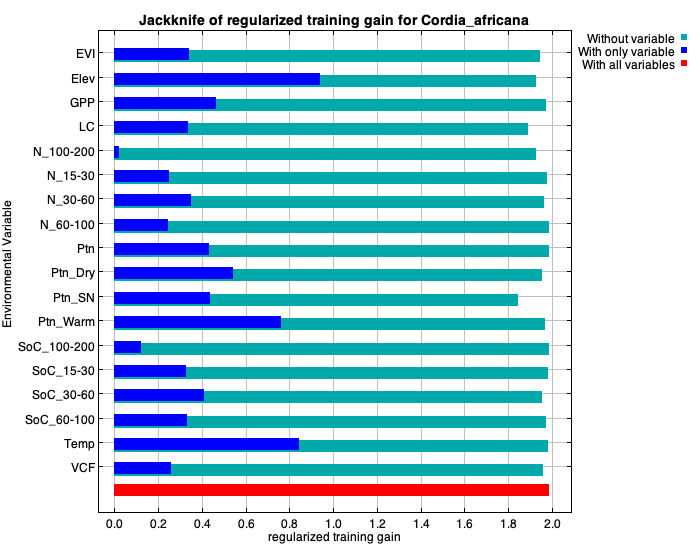

Supplement: Supplementary file 6 — Supplementary Material 6 [file 41598_2025_26400_MOESM6_ESM.zip › Supplementary file 1/plots/Cordia_africana_jacknife.png]

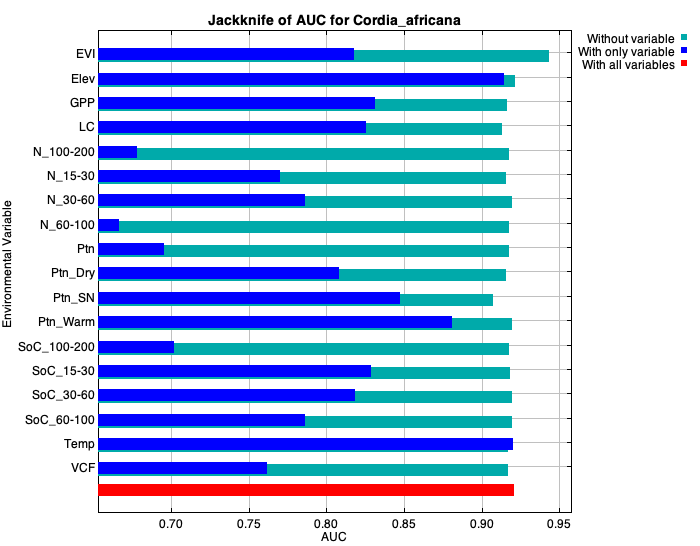

Supplement: Supplementary file 6 — Supplementary Material 6 [file 41598_2025_26400_MOESM6_ESM.zip › Supplementary file 1/plots/Cordia_africana_jacknife_auc.png]

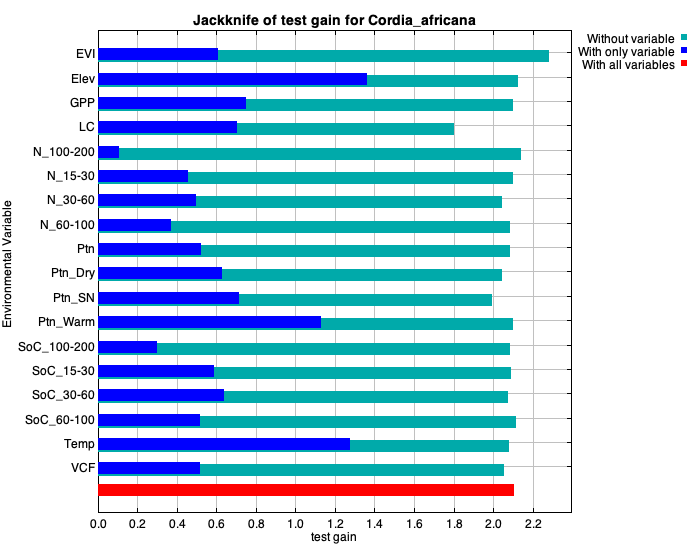

Supplement: Supplementary file 6 — Supplementary Material 6 [file 41598_2025_26400_MOESM6_ESM.zip › Supplementary file 1/plots/Cordia_africana_jacknife_test.png]

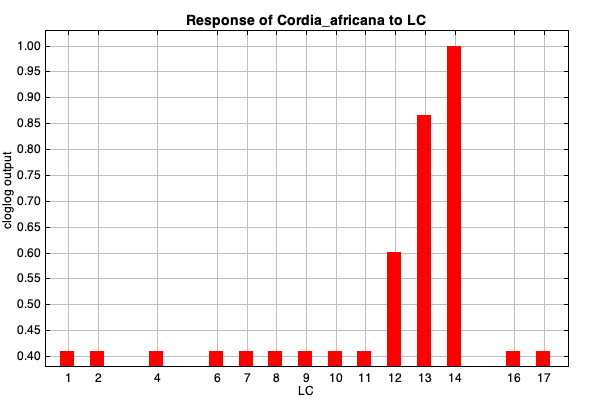

Supplement: Supplementary file 6 — Supplementary Material 6 [file 41598_2025_26400_MOESM6_ESM.zip › Supplementary file 1/plots/Cordia_africana_LC.png]

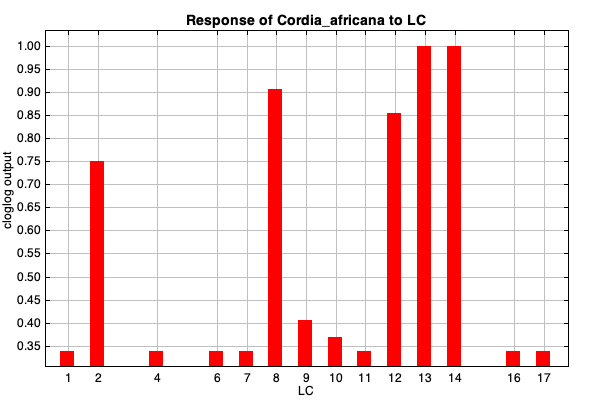

Supplement: Supplementary file 6 — Supplementary Material 6 [file 41598_2025_26400_MOESM6_ESM.zip › Supplementary file 1/plots/Cordia_africana_LC_only.png]

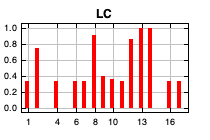

Supplement: Supplementary file 6 — Supplementary Material 6 [file 41598_2025_26400_MOESM6_ESM.zip › Supplementary file 1/plots/Cordia_africana_LC_only_thumb.png]

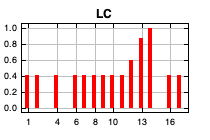

Supplement: Supplementary file 6 — Supplementary Material 6 [file 41598_2025_26400_MOESM6_ESM.zip › Supplementary file 1/plots/Cordia_africana_LC_thumb.png]
