# Supplementary figures and images for: Elemental concentration and spatial distribution of wild edible fruits and implications for dietary mineral intake in Ethiopia (part 2 of 2)
Source: Sci Rep. 2025 Nov 27;15:42307. doi: 10.1038/s41598-025-26400-7 (PMC12661052; doi:10.1038/s41598-025-26400-7)

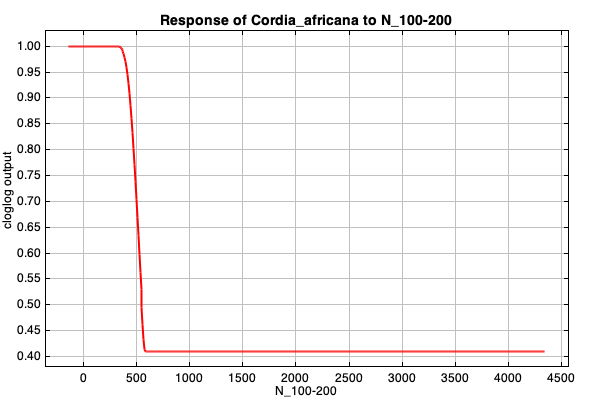

Supplement: Supplementary file 6 — Supplementary Material 6 [file 41598_2025_26400_MOESM6_ESM.zip › Supplementary file 1/plots/Cordia_africana_N_100-200.png]

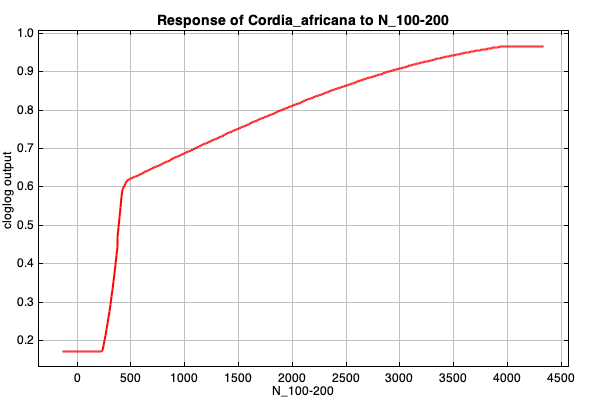

Supplement: Supplementary file 6 — Supplementary Material 6 [file 41598_2025_26400_MOESM6_ESM.zip › Supplementary file 1/plots/Cordia_africana_N_100-200_only.png]

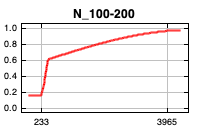

Supplement: Supplementary file 6 — Supplementary Material 6 [file 41598_2025_26400_MOESM6_ESM.zip › Supplementary file 1/plots/Cordia_africana_N_100-200_only_thumb.png]

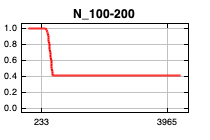

Supplement: Supplementary file 6 — Supplementary Material 6 [file 41598_2025_26400_MOESM6_ESM.zip › Supplementary file 1/plots/Cordia_africana_N_100-200_thumb.png]

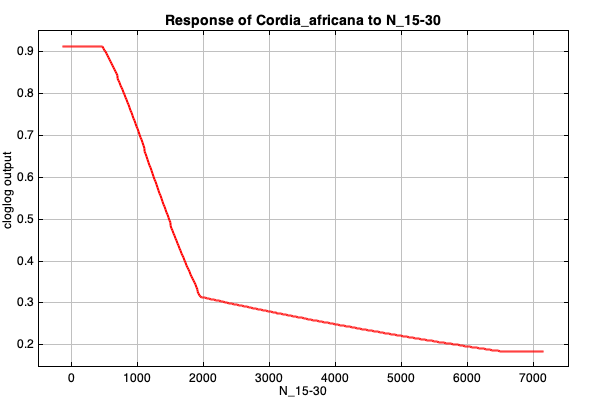

Supplement: Supplementary file 6 — Supplementary Material 6 [file 41598_2025_26400_MOESM6_ESM.zip › Supplementary file 1/plots/Cordia_africana_N_15-30.png]

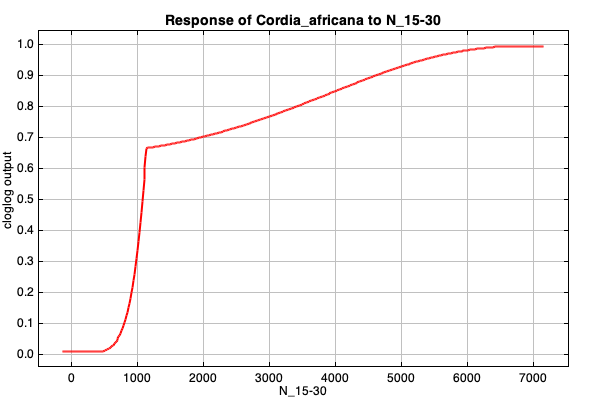

Supplement: Supplementary file 6 — Supplementary Material 6 [file 41598_2025_26400_MOESM6_ESM.zip › Supplementary file 1/plots/Cordia_africana_N_15-30_only.png]

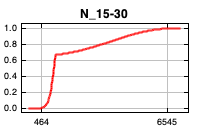

Supplement: Supplementary file 6 — Supplementary Material 6 [file 41598_2025_26400_MOESM6_ESM.zip › Supplementary file 1/plots/Cordia_africana_N_15-30_only_thumb.png]

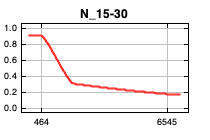

Supplement: Supplementary file 6 — Supplementary Material 6 [file 41598_2025_26400_MOESM6_ESM.zip › Supplementary file 1/plots/Cordia_africana_N_15-30_thumb.png]

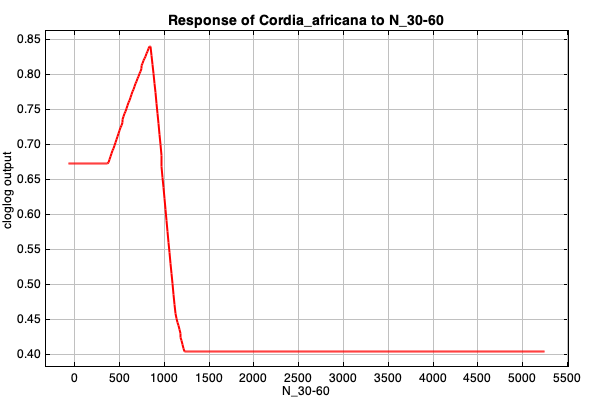

Supplement: Supplementary file 6 — Supplementary Material 6 [file 41598_2025_26400_MOESM6_ESM.zip › Supplementary file 1/plots/Cordia_africana_N_30-60.png]

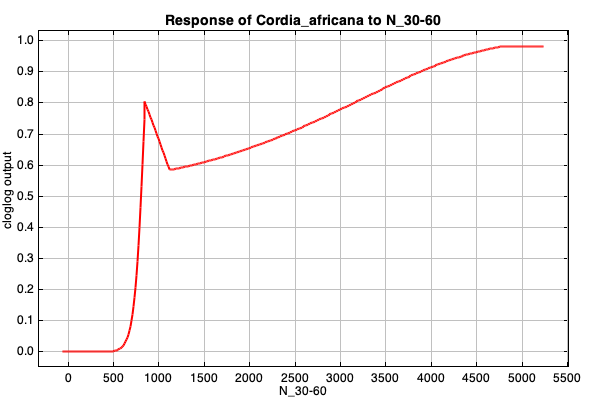

Supplement: Supplementary file 6 — Supplementary Material 6 [file 41598_2025_26400_MOESM6_ESM.zip › Supplementary file 1/plots/Cordia_africana_N_30-60_only.png]

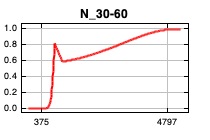

Supplement: Supplementary file 6 — Supplementary Material 6 [file 41598_2025_26400_MOESM6_ESM.zip › Supplementary file 1/plots/Cordia_africana_N_30-60_only_thumb.png]

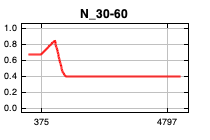

Supplement: Supplementary file 6 — Supplementary Material 6 [file 41598_2025_26400_MOESM6_ESM.zip › Supplementary file 1/plots/Cordia_africana_N_30-60_thumb.png]

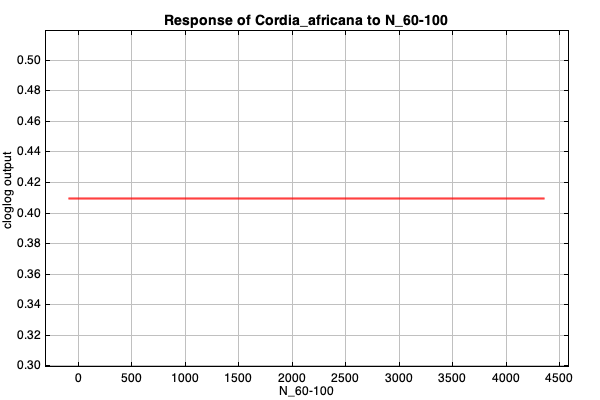

Supplement: Supplementary file 6 — Supplementary Material 6 [file 41598_2025_26400_MOESM6_ESM.zip › Supplementary file 1/plots/Cordia_africana_N_60-100.png]

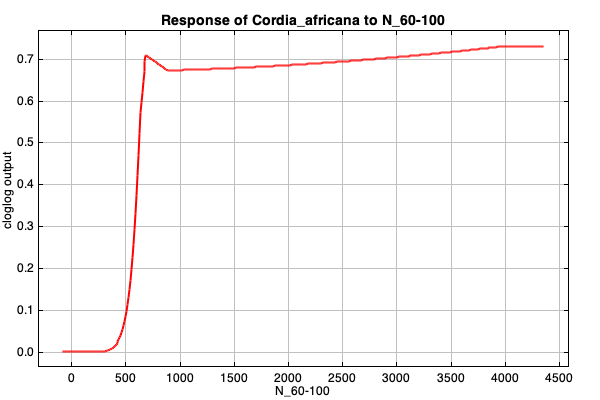

Supplement: Supplementary file 6 — Supplementary Material 6 [file 41598_2025_26400_MOESM6_ESM.zip › Supplementary file 1/plots/Cordia_africana_N_60-100_only.png]

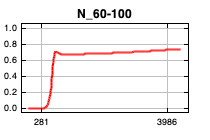

Supplement: Supplementary file 6 — Supplementary Material 6 [file 41598_2025_26400_MOESM6_ESM.zip › Supplementary file 1/plots/Cordia_africana_N_60-100_only_thumb.png]

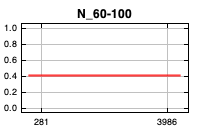

Supplement: Supplementary file 6 — Supplementary Material 6 [file 41598_2025_26400_MOESM6_ESM.zip › Supplementary file 1/plots/Cordia_africana_N_60-100_thumb.png]

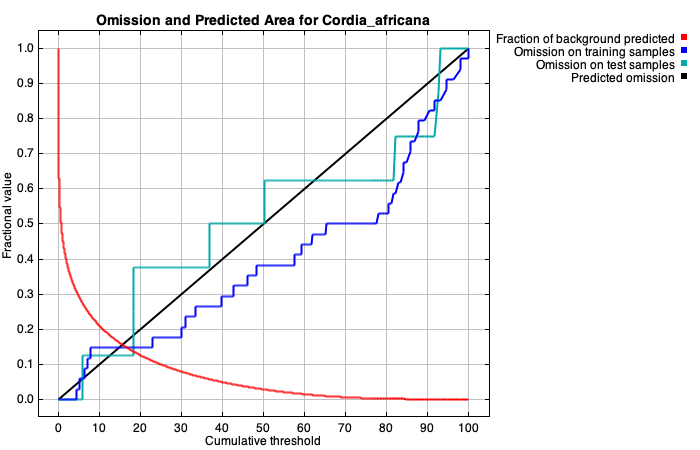

Supplement: Supplementary file 6 — Supplementary Material 6 [file 41598_2025_26400_MOESM6_ESM.zip › Supplementary file 1/plots/Cordia_africana_omission.png]

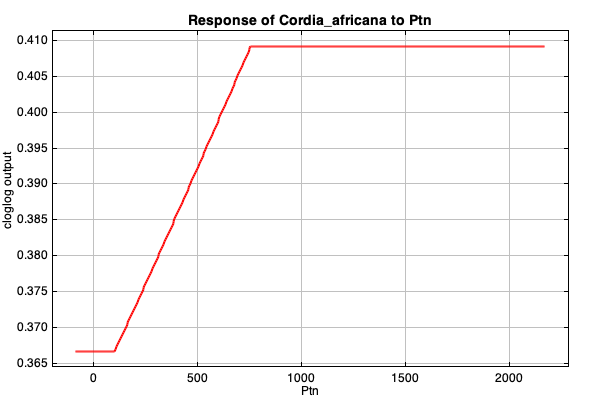

Supplement: Supplementary file 6 — Supplementary Material 6 [file 41598_2025_26400_MOESM6_ESM.zip › Supplementary file 1/plots/Cordia_africana_Ptn.png]

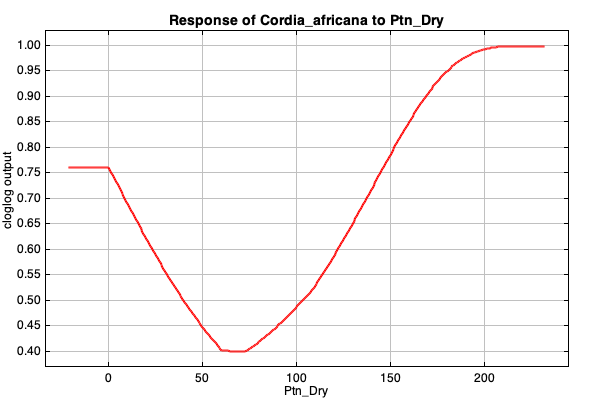

Supplement: Supplementary file 6 — Supplementary Material 6 [file 41598_2025_26400_MOESM6_ESM.zip › Supplementary file 1/plots/Cordia_africana_Ptn_Dry.png]

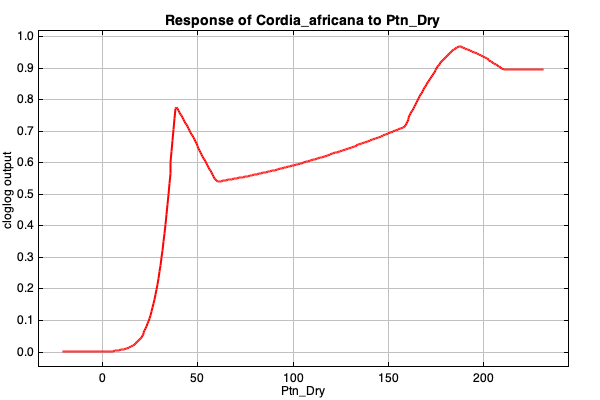

Supplement: Supplementary file 6 — Supplementary Material 6 [file 41598_2025_26400_MOESM6_ESM.zip › Supplementary file 1/plots/Cordia_africana_Ptn_Dry_only.png]

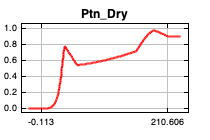

Supplement: Supplementary file 6 — Supplementary Material 6 [file 41598_2025_26400_MOESM6_ESM.zip › Supplementary file 1/plots/Cordia_africana_Ptn_Dry_only_thumb.png]

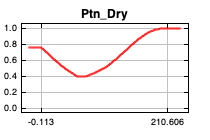

Supplement: Supplementary file 6 — Supplementary Material 6 [file 41598_2025_26400_MOESM6_ESM.zip › Supplementary file 1/plots/Cordia_africana_Ptn_Dry_thumb.png]

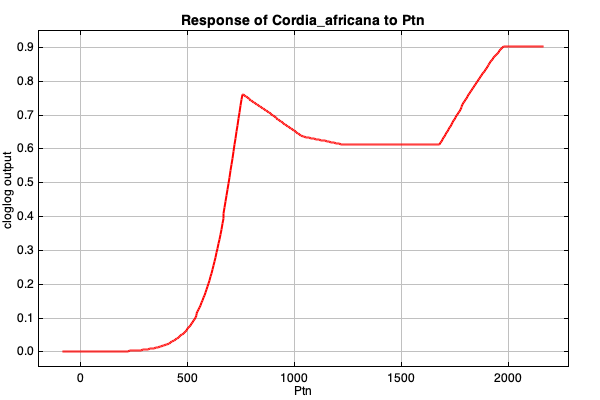

Supplement: Supplementary file 6 — Supplementary Material 6 [file 41598_2025_26400_MOESM6_ESM.zip › Supplementary file 1/plots/Cordia_africana_Ptn_only.png]

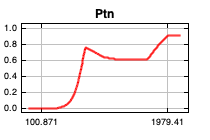

Supplement: Supplementary file 6 — Supplementary Material 6 [file 41598_2025_26400_MOESM6_ESM.zip › Supplementary file 1/plots/Cordia_africana_Ptn_only_thumb.png]

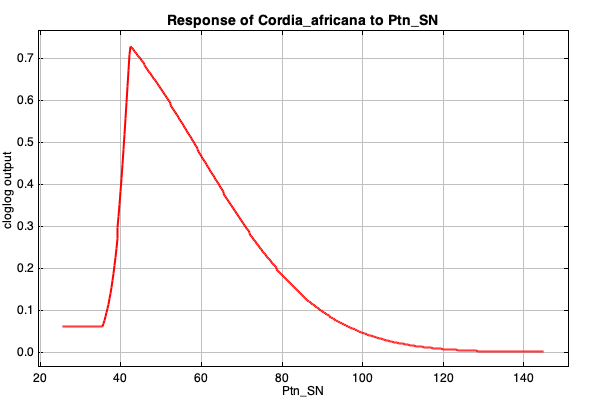

Supplement: Supplementary file 6 — Supplementary Material 6 [file 41598_2025_26400_MOESM6_ESM.zip › Supplementary file 1/plots/Cordia_africana_Ptn_SN.png]

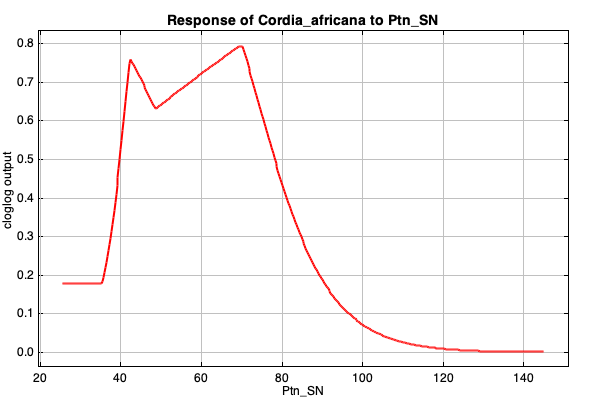

Supplement: Supplementary file 6 — Supplementary Material 6 [file 41598_2025_26400_MOESM6_ESM.zip › Supplementary file 1/plots/Cordia_africana_Ptn_SN_only.png]

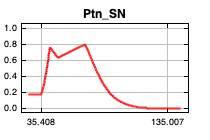

Supplement: Supplementary file 6 — Supplementary Material 6 [file 41598_2025_26400_MOESM6_ESM.zip › Supplementary file 1/plots/Cordia_africana_Ptn_SN_only_thumb.png]

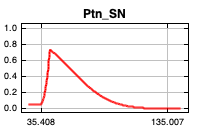

Supplement: Supplementary file 6 — Supplementary Material 6 [file 41598_2025_26400_MOESM6_ESM.zip › Supplementary file 1/plots/Cordia_africana_Ptn_SN_thumb.png]

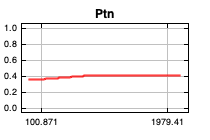

Supplement: Supplementary file 6 — Supplementary Material 6 [file 41598_2025_26400_MOESM6_ESM.zip › Supplementary file 1/plots/Cordia_africana_Ptn_thumb.png]

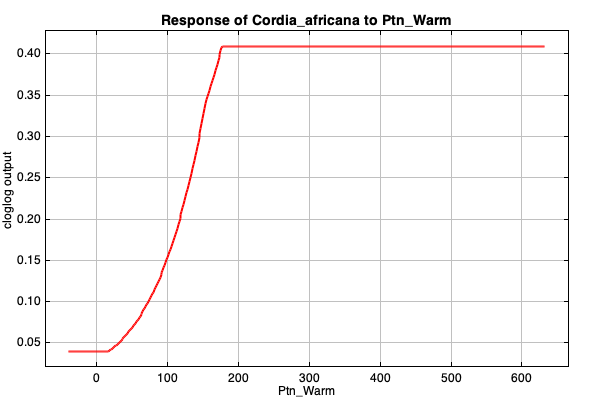

Supplement: Supplementary file 6 — Supplementary Material 6 [file 41598_2025_26400_MOESM6_ESM.zip › Supplementary file 1/plots/Cordia_africana_Ptn_Warm.png]

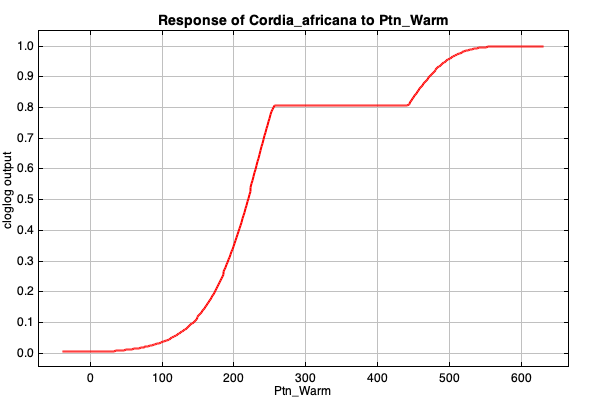

Supplement: Supplementary file 6 — Supplementary Material 6 [file 41598_2025_26400_MOESM6_ESM.zip › Supplementary file 1/plots/Cordia_africana_Ptn_Warm_only.png]

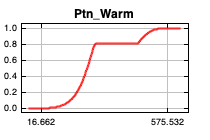

Supplement: Supplementary file 6 — Supplementary Material 6 [file 41598_2025_26400_MOESM6_ESM.zip › Supplementary file 1/plots/Cordia_africana_Ptn_Warm_only_thumb.png]

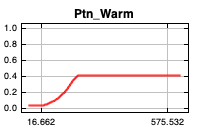

Supplement: Supplementary file 6 — Supplementary Material 6 [file 41598_2025_26400_MOESM6_ESM.zip › Supplementary file 1/plots/Cordia_africana_Ptn_Warm_thumb.png]

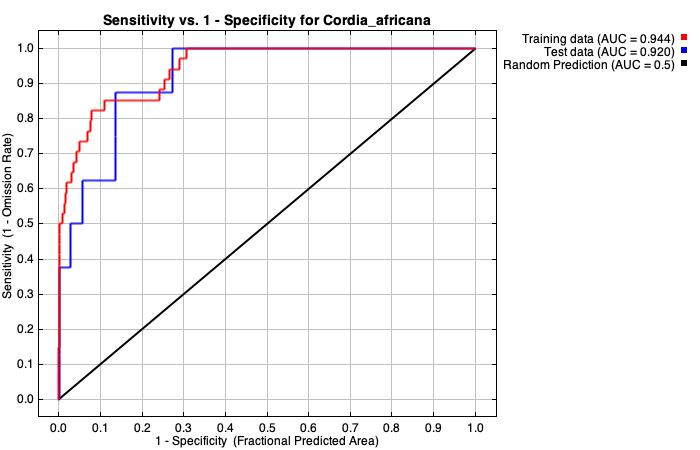

Supplement: Supplementary file 6 — Supplementary Material 6 [file 41598_2025_26400_MOESM6_ESM.zip › Supplementary file 1/plots/Cordia_africana_roc.png]

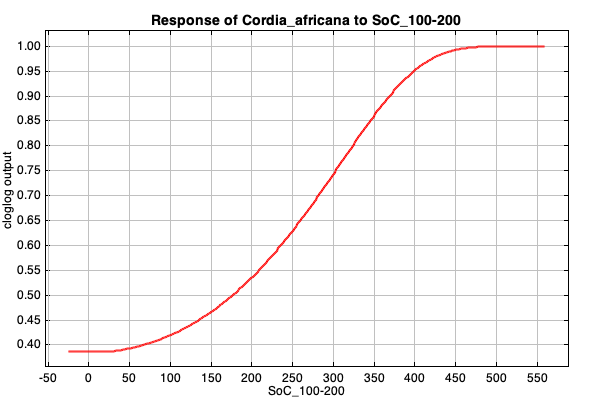

Supplement: Supplementary file 6 — Supplementary Material 6 [file 41598_2025_26400_MOESM6_ESM.zip › Supplementary file 1/plots/Cordia_africana_SoC_100-200.png]

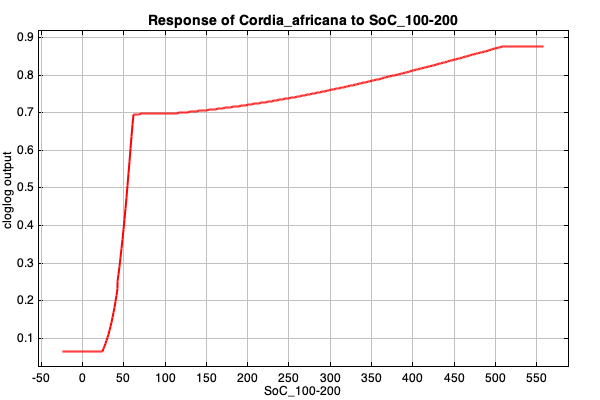

Supplement: Supplementary file 6 — Supplementary Material 6 [file 41598_2025_26400_MOESM6_ESM.zip › Supplementary file 1/plots/Cordia_africana_SoC_100-200_only.png]

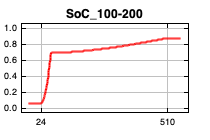

Supplement: Supplementary file 6 — Supplementary Material 6 [file 41598_2025_26400_MOESM6_ESM.zip › Supplementary file 1/plots/Cordia_africana_SoC_100-200_only_thumb.png]

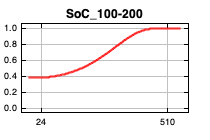

Supplement: Supplementary file 6 — Supplementary Material 6 [file 41598_2025_26400_MOESM6_ESM.zip › Supplementary file 1/plots/Cordia_africana_SoC_100-200_thumb.png]

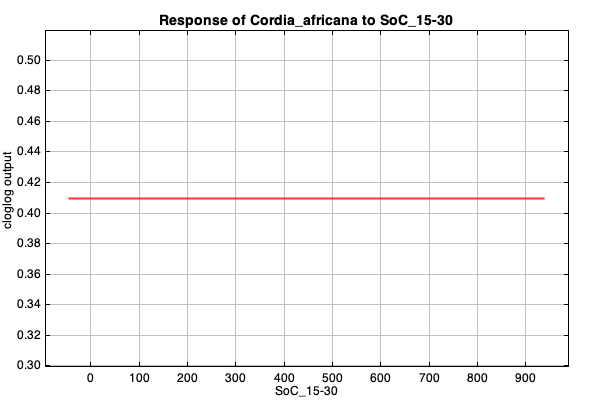

Supplement: Supplementary file 6 — Supplementary Material 6 [file 41598_2025_26400_MOESM6_ESM.zip › Supplementary file 1/plots/Cordia_africana_SoC_15-30.png]

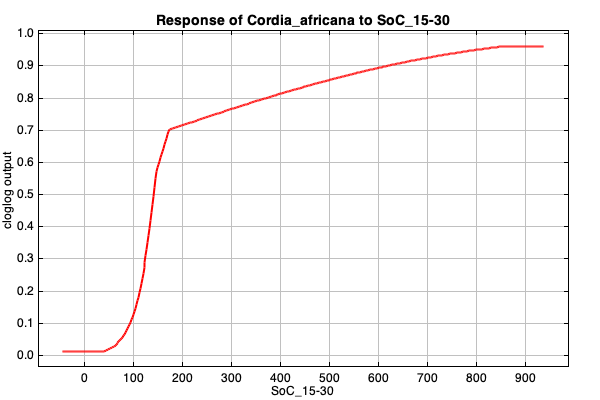

Supplement: Supplementary file 6 — Supplementary Material 6 [file 41598_2025_26400_MOESM6_ESM.zip › Supplementary file 1/plots/Cordia_africana_SoC_15-30_only.png]

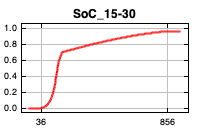

Supplement: Supplementary file 6 — Supplementary Material 6 [file 41598_2025_26400_MOESM6_ESM.zip › Supplementary file 1/plots/Cordia_africana_SoC_15-30_only_thumb.png]

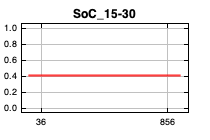

Supplement: Supplementary file 6 — Supplementary Material 6 [file 41598_2025_26400_MOESM6_ESM.zip › Supplementary file 1/plots/Cordia_africana_SoC_15-30_thumb.png]

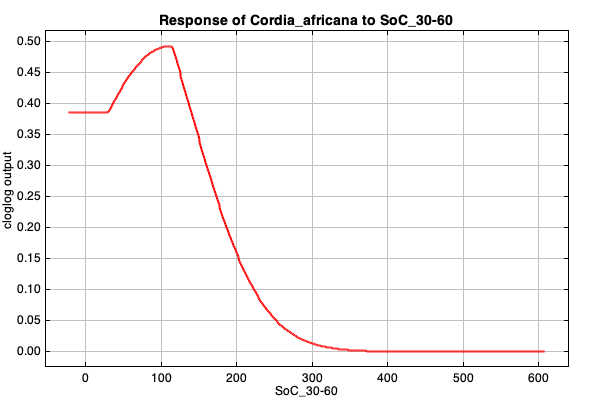

Supplement: Supplementary file 6 — Supplementary Material 6 [file 41598_2025_26400_MOESM6_ESM.zip › Supplementary file 1/plots/Cordia_africana_SoC_30-60.png]

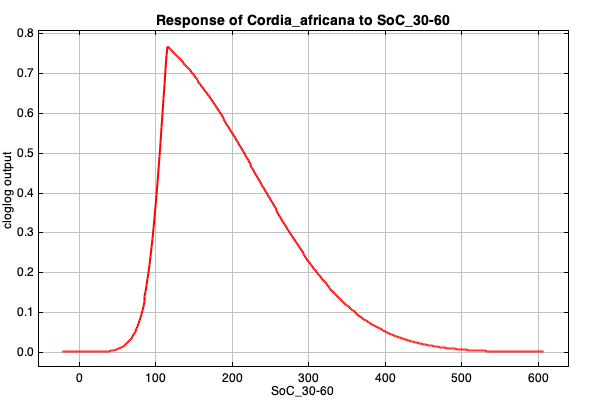

Supplement: Supplementary file 6 — Supplementary Material 6 [file 41598_2025_26400_MOESM6_ESM.zip › Supplementary file 1/plots/Cordia_africana_SoC_30-60_only.png]
