# Supplementary material for: Elemental concentration and spatial distribution of wild edible fruits and implications for dietary mineral intake in Ethiopia
Source: Sci Rep. 2025 Nov 27;15:42307. doi: 10.1038/s41598-025-26400-7 (PMC12661052; doi:10.1038/s41598-025-26400-7)
Supplement: Supplementary file 11 — Supplementary Material 11 [file 41598_2025_26400_MOESM11_ESM.docx]

# Supplements

Supplementary Table 1. Data on elemental concentrations in fruit and soil, with additional relevant sample details.

Supplementary Table 2. Fruit elemental detection limits and analyte recovery rates from certified reference materials.

Supplementary Table 3. Descriptive statistics on fruit elemental concentration, 100 g fruit consumption's contribution to adolescent male reference nutrient intake (RNI) for selected elements, and corresponding soil elemental concentration.

Supplementary Table 4. Welch’s ANOVA on elemental concentration per 100 g fresh weight across different fruit species. This analysis was conducted solely on species with at least three replicates and essential elements for human nutrition. The sheets within display the outcomes of ANOVA and the post-hoc analysis for each element, denoted by specific symbols. For post-hoc sheets, the initial two columns list the species being compared, using their abbreviated names. The subsequent columns provide information on the mean difference (diff) and the p-value (pval). Instances of significant disparities between species are indicated with a light red highlight in the relevant cells.

Fig. Sup. 1. Species point occurrence data used in Maxent modelling. The species included were *C. spinarum, C. africana, D. caffra, F. sur, F. sycomorus, M. kummel, P. peruviana, R. apetalus, R. steudneri, S. guineense* and *Z. spina-christi* across the Oromia and SNNP regions of Ethiopia. The number of occurrence data points for each species also presented in a table.

Fig. Sup. 2. Box plots showing elemental concentrations in 27 different edible fruit species. The left panel presents concentrations based on 100 g of fresh weight, while the right panel displays concentrations per kg of dry matter for each element. The box plots are ordered by the median concentration values on a fresh weight basis, indicated by orange lines. Data outliers are indicated as "o". Where data is available, elemental concentrations in strawberries are provided as a reference (vertical red dashed lines).

Supplementary file 1. Log file for the Maxnet model included in the .html files per species, detailing the used model parameters, rates of omission and commission, Area Under the Receiver Operating Characteristic Curve (AUC) values, images displaying model training and testing points, percentage contributions and permutation significance of environmental variables, and the Jackknife test of environmental variables. In the log folder, the model's predicted probability of species presence is contained in the .asc files and plots are found in the plots folder.
